# Escaping the OR: a pilot study of a Jigsaw-based workshop to teach preoperative assessment in internal medicine residency

**DOI:** 10.1186/s12909-026-09419-w

**Published:** 2026-05-16

**Authors:** Ruth Abeles, Laura Eger, Tanner Slayden, Stacy Charat, Saloni Kumar Maharaj

**Affiliations:** 1 University of California, San Diego, USA; 2 School of Medicine, Stanford University, Stanford, USA

**Keywords:** Perioperative Risk Assessment, Gamification, Resident Education

## Abstract

**Background:**

Preoperative assessment is a key but inconsistently taught skill in internal medicine residency, particularly in the ambulatory setting. Many residents report low confidence and limited formal training.

**Objective:**

To evaluate the impact of a structured, gamified Jigsaw-based workshop on internal medicine residents’ knowledge acquisition and self-reported confidence in preoperative assessment.

**Methods:**

In 2025, we conducted a quasi-experimental pilot study at the University of California, San Diego. Primary Care Track residents (intervention group) completed a three-hour interactive Jigsaw workshop on four domains of preoperative management: cardiovascular risk, medication management, anticoagulation, and considerations for special populations. Residents in the control group received standard residency training in preoperative medicine through routine clinical exposure during inpatient rotations (wards, intensive care unit, and consult services) and outpatient settings, including continuity clinic and ambulatory electives. Additional instruction occurred through the residency’s protected didactic curriculum, including Wednesday School and Friday School, four-hour conference blocks covering rotating internal medicine topics. Preoperative assessment was addressed within this broader curriculum but not through a dedicated standardized program. Pre- and post-surveys assessed knowledge (multiple-choice) and confidence (Likert scale). Due to the small sample size, formal hypothesis testing with p-values was not performed. Instead, effect sizes were calculated to estimate the magnitude of differences between the intervention and control groups.

**Results:**

A needs assessment of 159 residents (12% response) revealed low confidence in perioperative care; over half reported no structured training. In the control group (*n* = 11 pre, *n* = 10 post), notable gains are observed in special population (40% to 49%, effect size = 0.45) and anticoagulation (41.1% to 48.50%, effect size = 2.00). Cardiovascular risk and medical management remain stable (45.40% to 39.40%, effect size = 0.18, and 60.60% to 56.1%, effect size = 0.04, respectively). In the intervention group (*n* = 11, *n* = 10 post), all categories show notable gains, with special populations and cardiovascular risk reaching statistical significance: cardiovascular risk group (37.50% to 66.70%, effect size = 4.04), medication management (64.5% to 75%, effect size = 0.45), special population (45% to 95%, effect size = 4.26), and anticoagulation (45.80% to 58.30%, effect size = 0.42). In the control group (*n* = 11 pre, *n* = 10 post), mean confidence scores remained largely unchanged, with minimal effect (2.35 vs 2.38; effect size = 0.10). In contrast, the intervention group (*n* = 8 pre, *n* = 8 post) demonstrated substantial improvements in confidence across all domains following the workshop, with mean Likert scores increasing from 1.86 to 3.18 (effect size = 2.48). Qualitative feedback showed that residents valued the interactive, case-based format and the gamified “Escape the OR” activity.

**Conclusions:**

A single Jigsaw-based workshop integrated into a primary care residency curriculum was observed to be associated with improvements in resident knowledge and confidence in perioperative assessment. These early findings suggest that structured, interactive workshops may help address gaps in perioperative education and support further evaluation of learner-centered instructional strategies in larger training settings.

**Supplementary Information:**

The online version contains supplementary material available at 10.1186/s12909-026-09419-w.

## Background

Preoperative assessment is a core competency for internal medicine residents, especially in ambulatory settings where surgical clearance is frequently requested. Despite its importance, many residency programs lack a structured outpatient preoperative curriculum, resulting in inconsistent trainee exposure and limited confidence in managing such assessments [1, 2]. Traditional didactic models may not meet the needs of millennial and Gen Z learners, who benefit from more active, collaborative, and learner-centered approaches to education [3, 4].

To better define local learning gaps, we conducted a needs assessment of internal medicine residents at the University of California, San Diego. Among 19 respondents, cardiac risk assessment and anticoagulation emerged as the most desired learning topics (mean interest scores 4.2 and 4.16 out of 5, respectively), while medication management (63%) and anticoagulation (58%) were rated most challenging. Many residents reported limited or no structured training in preoperative care, particularly for inpatient evaluations. These findings underscored both a strong interest and a critical educational gap, supporting the need for a formalized curriculum.

The Jigsaw Teaching Method is a cooperative learning strategy in which learners become “experts” on a topic and then teach their peers in small groups [5]. This method has demonstrated effectiveness in promoting engagement, retention, and communication skills across health professions education—including pharmacy [6], dentistry [7], nursing [8], and internal medicine [9, 10]. It has also shown promise in teaching complex concepts, such as patient safety and women's health in internal medicine residency programs [9, 10]. Furthermore, the Jigsaw format has proven adaptable to distance learning during the COVID-19 pandemic [11].

Despite its growing use, the Jigsaw method has not been formally applied to preoperative assessment education in internal medicine nor integrated into a journal club–style format. To address this gap, we developed and piloted a workshop using the Jigsaw teaching method to teach four core domains of outpatient preoperative medicine: cardiovascular risk assessment, med management, antiplatelet and anticoagulation, as well as considerations for special population. The study evaluated the early impact of this interactive educational intervention on resident knowledge and confidence in preoperative assessment among a small group of primary care residents.

## Methods

### Study design and setting

We conducted a quasi-experimental pilot study at the University of California, San Diego (UCSD) Internal Medicine Residency Program between April and May of 2025. The purpose of this pilot study was to examine the educational impact of a single structured, interactive workshop using the Jigsaw Teaching Method on resident knowledge and confidence in preoperative assessment compared with standard residency training. The intervention was delivered within the ambulatory curriculum of the Primary Care Track over a three-hour workshop.

### Participants and group assignment

A total of 159 internal medicine residents were eligible to participate in the study. In April 2025, a voluntary needs assessment survey was distributed to all residents to assess educational gaps and guide the feasible design of a structured preoperative assessment workshop. All 8 currently active residents in the Primary Care Track were pre-selected to serve as the pilot intervention group based on curriculum timing and ambulatory availability. The intervention group participated in the Jigsaw-based three-hour workshop in May 2025. This cohort was selected because it provided a controlled educational environment with protected didactic time and curricular flexibility, allowing for a mid-year integration of the intervention.

The remaining respondents from the categorical internal medicine residency were invited to voluntarily join as the control group. Residents in the control group received standard residency training in preoperative medicine through routine clinical experiences within the Internal Medicine residency curriculum. This included exposure during inpatient rotations (general medicine wards, intensive care units, and inpatient consult services) where residents participate in perioperative evaluation and management of hospitalized patients. Additional exposure occurred through outpatient clinical settings, including continuity clinic and ambulatory elective rotations, where residents may encounter patients requiring preoperative assessment. In addition to clinical exposure, all residents participated in the program’s established didactic curriculum, which includes structured educational conferences known as Wednesday School and Friday School. These weekly sessions, occurring year long, consist of protected didactic blocks totaling approximately four hours and cover rotating topics across the breadth of internal medicine. Preoperative assessment content may be addressed during these sessions as part of the broader curriculum but is not delivered through a dedicated standardized curriculum.

### Data collection and measures

Participants were invited to participate in voluntary pre- and post-intervention surveys that included:

- 15 multiple-choice knowledge questions
- 8 questions utilizing Likert-scale confidence ratings across core domains of preoperative care
- Demographics and self-reported sources of prior training

The pre- and post-intervention surveys were developed by the study authors to assess the knowledge and confidence across the four domains (Supplementary File 1). Survey data were collected anonymously using Qualtrics. Individual responses were not linked, so survey responses were analyzed at the group level. Knowledge scores across the four domains were summarized for pre- and post-intervention surveys. Because individual responses could not be paired, group-level mean differences and effect sizes (Cohen’s d) were calculated to estimate the magnitude of change. Confidence ratings on Likert scales were analyzed similarly.

### Educational intervention

The educational intervention was a three-hour workshop built around the Jigsaw Teaching Method and active, case-based learning. It focused on four key domains of outpatient preoperative care:

1. Cardiovascular risk assessment
2. Medication management
3. Anticoagulation strategies
4. Considerations for Special populations (e.g., bariatric surgery, pulmonary/kidney/liver considerations, venous thromboembolism (VTE) risk assessments, immunosuppressants)

Learners were first assigned to “expert groups” and provided with a guided PowerPoint, a structured worksheet, and selected readings drawn from the most current literature on preoperative management. These readings—focused on cardiovascular risk, medication management, anticoagulation, and special populations—formed the foundation for a journal club–style review within each expert group. After this self-directed preparation, residents joined “home groups” composed of one expert from each domain. Within these groups, learners applied their collective knowledge to collaboratively solve two complex, integrative patient cases simulating real-world preoperative scenarios.

### Expert group materials and journal club format

All expert learning materials were developed using the updated perioperative medicine guidelines [1, 2, 12–20] as focused “expert slices” designed to be efficiently digestible within a short preparation period. Each domain—cardiovascular risk, medication management, anticoagulation, and special populations—was accompanied by a concise expert packet that included a guided PowerPoint presentation, a structured worksheet, and distilled primary literature or guideline excerpts. These materials functioned as a mini-journal club, enabling each learner to gain foundational knowledge and key takeaways within their assigned topic area. The goal was to equip learners with enough clarity and confidence to serve as peer educators during the subsequent home group case-solving session, where they applied their domain knowledge collaboratively to manage complex preoperative scenarios.

### Debrief and game

Following the case discussions, all participants reconvened for a faculty-led debrief that reinforced key concepts and provided clarification on challenging points. The session concluded with a gamified team activity, “Escape the OR,” in which learners worked in small groups to solve five rounds of clinical puzzles. Each completed round yielded a virtual “key” that unlocked progress toward escaping the operating room—designed to reinforce concepts and promote engagement through playful competition.

### Data analysis

Because surveys were administered anonymously and participants were not asked to generate a unique study identifier, pre- and post-workshop responses could not be reliably paired at the individual level. As a result, analyses were conducted using aggregated group-level data, with mean scores calculated based on response frequencies for the control and intervention groups. This limitation will be addressed in future studies by incorporating unique participant identifiers to enable paired analyses of individual-level changes over time.

We evaluated knowledge acquisition across four key domains—cardiovascular risk assessment, medication management, special populations, and anticoagulation—by calculating the mean percentage of correct responses before and after the intervention. For each group (control and intervention), the percentage of correct answers was derived by dividing the number of correct responses by the total number of responses for each question. Pre- and post-survey responses were analyzed to assess changes in self-reported confidence across seven domains of preoperative assessment. Confidence was rated on a 5-point Likert scale (1 = Not at all confident to 5 = Extremely confident). Given the small sample size typical of pilot studies, effect sizes (Cohen’s d) were calculated to estimate the magnitude of the intervention’s educational impact. We categorized effect sizes as small (0.2), medium (0.5), large (0.8), and very large (≥ 1.3) [21].

### Ethical considerations

This study was reviewed and approved by the University of California, San Diego Institutional Review Board (Protocol #810,740) and was conducted in accordance with the principles of the Declaration of Helsinki. All participants provided written informed consent prior to participation.

## Results

### Needs assessment findings

In April 2025, a voluntary needs assessment was distributed to all 159 internal medicine residents at our institution. A total of 19 residents (12% response rate) responded, with an even distribution across PGY-1 (37%), PGY-2 (37%), and PGY-3 (26%). Most respondents reported only moderate confidence in key areas of preoperative assessment, including cardiac risk stratification (74% moderately confident) and applying ACC/AHA guidelines (47% moderately confident). Confidence was lower in medication management and anticoagulation, with nearly half of respondents identifying as only slightly confident in each domain. Notably, over half (53%) reported receiving no formal education in perioperative medicine during residency. The most frequently cited challenges were medication management (63%), anticoagulation (58%), and optimization of patients with complex comorbidities, such as bariatric or transplant candidates (53%). Interest in structured education was high, with average Likert scores ≥ 3.9 across all domains, and highest for cardiac risk assessment (4.21) and anticoagulation management (4.16). Preferred learning formats included interactive workshops (74%), in-person lectures (63%), and case-based discussions (63%).

### Knowledge scores

In the control group (*n* = 11 pre, *n* = 10 post), notable gains were observed in special population (40% to 49%, effect size = 0.45) and anticoagulation (41.1% to 48.50%, effect size = 2.00,). Cardiovascular risk and medical management remain stable (45.40% to 39.40%, effect size = 0.18, and 60.60% to 56.1%, effect size = 0.04 respectively).

In the intervention group (*n* = 11, *n* = 10 post), all categories showed notable gains: cardiovascular risk group (37.50% to 66.70%, effect size = 4.04), medication management (64.5% to 75%, effect size = 0.45), special population (45% to 95%, effect size = 4.26,), and anticoagulation (45.80% to 58.30%, effect size = 0.42).

**Fig. 1:**
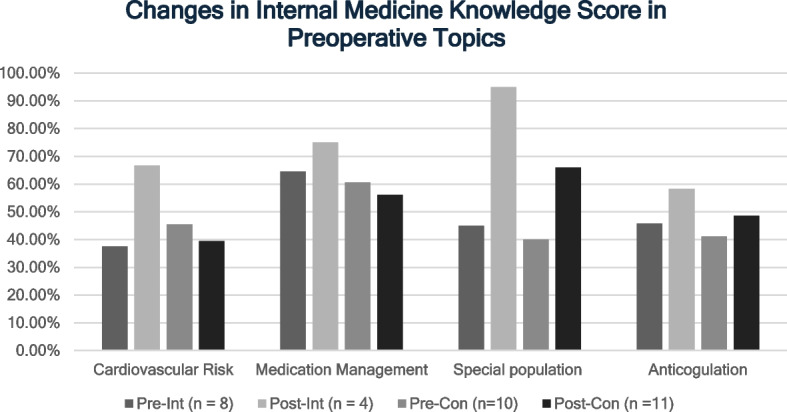
In the control group (*n* = 11 pre, *n* = 10 post), notable gains are observed in special population (40% to 49%) and anticoagulation (41.1% to 48.50%). Cardiovascular risk and medical management remain stable (45.40% to 39.40% and 60.60% to 56.1% respectively). In the intervention group (*n* = 11, *n* = 10 post), all categories show notable gains: cardiovascular risk group (37.50% to 66.70%), medication management (64.5% to 75%), special population (45% to 95%), and anticoagulation (45.80% to 58.30%)

Summary of Knowledge Score is presented in Fig. 1.

### Confidence scores

In the control group (*n* = 11 pre, *n* = 10 post), changes in mean confidence scores were generally small and inconsistent. In over half of the categories, post-test confidence scores were lower than pre-test scores, with a mean Likert scale score of 2.35 remaining stable in the post-test at 2.38, (effect size = 0.10). In the intervention group (*n* = 8 pre, *n* = 8 post) mean confidence scores showed consistent significant gains in confidence across all domains following the educational session, with Likert scale scores increasing across all domains from a mean of 1.86 to 3.18 (effect size = 2.48).

**Fig. 2:**
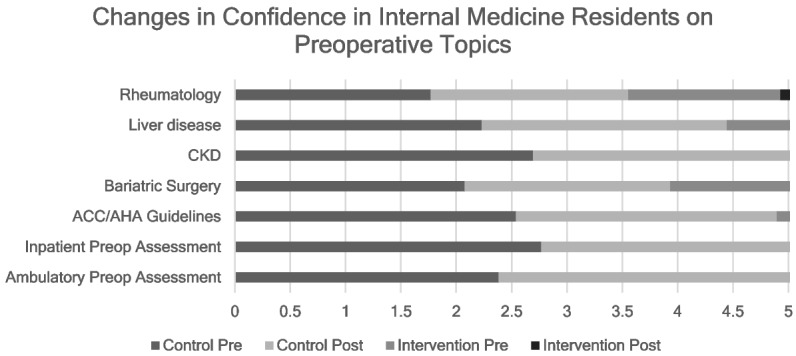
In the control group (*n* = 11 pre, *n* = 10 post), changes in mean confidence scores were generally small and less consistent. In over half of the categories, post-test confidence scores were lower than pre-test scores. In the intervention group (*n* = 8 pre, *n* = 8 post) mean confidence scores showed consistent gains in confidence across all domains following the educational session

Summary of finding is presented in Fig. 2.

### Training exposure

Analysis of prior exposure to preoperative training revealed that most residents in both groups had learned from clinical experience rather than structured curricula. When stratified by setting, a larger proportion of intervention group residents reported prior ambulatory exposure, while inpatient training was more commonly cited by the control group. There was no reported training in medical school setting.

### Learner feedback and engagement

Residents in the intervention group expressed high levels of enthusiasm for the workshop format, as evidenced by informal feedback and discussions. Anonymous qualitative feedback through survey expressed that participants particularly valued the case-based learning approach, peer teaching structure, and the real-world applicability of the content. This positive response highlights the effectiveness of the workshop in fostering engagement among participants. Other feedback noted that the material was dense and suggested allocating more time in future sessions to better absorb the readings and expert group discussions. Qualitative feedback from residents indicated that the gamified “Escape the OR” activity was one of the most enjoyable components of the workshop.

## Discussion

Preoperative evaluation is a core competency for internal medicine physicians, yet formal training in this area remains variable across residency programs. As surgical patients become increasingly medically complex, internists are more frequently involved in perioperative risk stratification and medical optimization. Recognizing this need, several institutions have developed structured perioperative curricula within internal medicine training. For example, Raslau et al. described a perioperative curriculum embedded within a general internal medicine consult rotation to address ACGME and ABIM training requirements [22]. Prior educational interventions have shown that structured teaching can improve resident knowledge in perioperative medicine. Yang et al. demonstrated that an interactive educational module on perioperative cardiac risk assessment significantly improved resident knowledge scores in a community internal medicine residency program [23]. Similarly, Hudspeth et al. reported that a case-based preoperative assessment teaching session improved knowledge and was preferred by learners over traditional lecture-based instruction [24]. Educational gaps in perioperative training may also extend earlier in the training continuum; McDonald et al. demonstrated improved knowledge, clinical decision-making, and preparedness among medical students completing a perioperative management elective prior to residency [25].

Consistent with these prior studies, our pilot study demonstrated that a brief, structured Jigsaw-based workshop improved resident knowledge and confidence in preoperative assessment. Residents participating in the workshop demonstrated substantial knowledge improvements across multiple domains of perioperative evaluation, with particularly large gains observed in cardiovascular risk assessment and special populations as well. These findings suggest that interactive and collaborative learning approaches may be effective strategies for teaching complex perioperative concepts. Residents who participated in the Jigsaw-based workshop demonstrated substantial improvements in self-reported confidence across all domains of preoperative assessment, while confidence in the control group remained largely unchanged. The large effect size observed in the intervention group suggests that the structured, interactive format may enhance residents’ perceived readiness to approach perioperative decision-making. Although confidence does not directly equate to clinical competence, these findings suggest that interactive peer-teaching strategies may help strengthen trainee preparedness in preoperative medicine.

The Jigsaw teaching method emphasizes collaborative learning in which participants first develop expertise within a focused topic area before teaching their peers and applying collective knowledge to clinical cases. This method has demonstrated effectiveness in promoting engagement, knowledge retention, and communication skills across health professions education, including pharmacy [6], dentistry [7], nursing [8], and internal medicine [9, 10].

Importantly, the Jigsaw method has not previously been applied to preoperative assessment education in internal medicine residency training. In our workshop, this collaborative learning model was combined with case-based discussion and a gamified activity (“Escape the OR”) that required residents to apply newly learned concepts to solve clinical problems. This format provided multiple opportunities for active participation and clinical reasoning, which may help explain the observed improvements in knowledge and confidence.

### Limitations

As a pilot study, several limitations should be acknowledged. First, the study was conducted at a single institution within a primary care track of an internal medicine residency program, which may limit generalizability to other training environments. This cohort was intentionally selected because it provided a controlled educational setting with protected didactic time and curricular flexibility, allowing mid-year implementation of the intervention. Second, the sample size was small, which limits statistical power and precludes definitive conclusions regarding effectiveness. To address this limitation, effect sizes were calculated to provide additional context for the magnitude of observed educational changes; however, the findings should still be interpreted cautiously and considered hypothesis-generating rather than confirmatory.

Additionally, inclusion of the entire categorical internal medicine residency program could have improved sample size and statistical power, but this was not feasible due to scheduling constraints and the fixed structure of the academic year. Another limitation is the challenge of fully characterizing the learning exposure to preoperative medicine within the control group, as standard residency training occurs through a combination of inpatient consultations, continuity clinic experiences, and didactic sessions that vary in timing and content. As a result, the comparison group likely experienced heterogeneous educational exposure. Another limitation of this study is that the intervention was compared with standard residency training rather than another structured educational approach. As a result, the study cannot determine whether the Jigsaw method itself is superior to other learner-centered instructional strategies, such as case-based workshops or flipped classroom models. Future studies comparing different active learning methods would help clarify the relative educational benefit of the Jigsaw approach. The observed improvements in learner confidence and domain-specific knowledge suggest that this approach may have educational value and support further evaluation through larger, multi-institutional studies.

### Future directions and implications for broader clinical education contexts

Although conducted within a single U.S. residency track, the core elements of this pilot intervention have relevance across international clinical education settings. Preoperative assessment is a universally required competency, and the cognitive processes involved—risk stratification, medication management, and integrated clinical reasoning—are consistent across health systems, even when specific guidelines differ. Given the pilot design and limited sample size, these findings should be interpreted as evidence of feasibility, acceptability, and preliminary signal detection rather than comparative effectiveness. The intervention was successfully implemented within existing curricular constraints and was associated with improved learner confidence, suggesting that structured, case-integrated active learning formats can be incorporated without major curricular restructuring. Educators internationally may reasonably conclude that a modular, Jigsaw-based perioperative workshop is adaptable to local practice standards and warrants further multi-site evaluation to determine generalizability and educational impact.

Future directions include expanding the curriculum to larger cohorts across multiple residency programs to improve generalizability and statistical power. Future studies should also incorporate unique participant identifiers to allow pairing of individual pre- and post-survey responses, enabling more precise assessment of knowledge and confidence changes attributable to the intervention. Additional work should evaluate the durability of knowledge and confidence gains over time and explore downstream outcomes, such as improvements in clinical decision-making and patient safety.

## Conclusion

This pilot study demonstrates the effects of implementing a brief, interactive preoperative medicine workshop within a primary care residency curriculum. Although limited by small sample size, the intervention was associated with favorable trends in resident knowledge and significant improvements in learner confidence. Together, these findings suggest that a Jigsaw-based, active-learning approach holds promise for addressing gaps in perioperative education and merits further evaluation in larger, multi-site training settings.

## Supplementary Information

Supplementary Material 1.

## Data Availability

The datasets generated and/or analyzed during the current study are not publicly available due to institutional privacy policies and the presence of identifiable information within the survey responses. De-identified data may be available from the corresponding author upon reasonable request and with permission from the University of California, San Diego.
