# Supplementary material for: Escaping the OR: a pilot study of a Jigsaw-based workshop to teach preoperative assessment in internal medicine residency
Source: BMC Med Educ. 2026 May 16;26:1097. doi: 10.1186/s12909-026-09419-w (PMC13348809; doi:10.1186/s12909-026-09419-w)
Supplement: Supplementary file 1 — Supplementary Material 1. [file 12909_2026_9419_MOESM1_ESM.zip › Complete_with_Docusign_Authorshipform.pdf]

Docusign Envelope ID: 08AC93CF-74A7-4761-BC72-D71E01F28E71

SPRINGER NATURE

Change of authorship request form - Journals (pre-acceptance)

Important information. Please read.

- This form should be used by authors to request any change in authorship (adding/deleting authors) including changes in corresponding authors. This form should not be used for name changes. Please fully complete all sections. Use black ink and block capitals and provide each author's full name with the given name first followed by the family name.

- By signing this declaration, all authors guarantee that the order of the authors are in accordance with their scientific contribution, if applicable as different conventions apply per discipline, and that only authors have been added who made a meaningful contribution to the work.

- Please note, in author collaborations where there is formal agreement for representing the collaboration, it is sufficient for the representative or legal guarantor (usually the corresponding author) to complete and sign the Authorship Change Form on behalf of all authors, **next to the added/removed author(s)**. **(Complete Section 3, followed by Section 6.)**
  In author collaborations where there is no formal agreement for representing the collaboration and **there are more than 10 authors**, one may sign for all, provided the signer appends correspondence that attests that each of the authors have agreed to the change **and the added/removed authors sign the form**. **(Complete Section 3, followed by Section 6.)**

- Please note, we cannot investigate or mediate any authorship disputes. If you are unable to obtain agreement from all authors (including those who you wish to be removed) you must refer the matter to your institution(s) for investigation. Please inform us if you need to do this.

- If you are not able to return a fully completed form within **30 days** of the date that it was sent to the author requesting the change, we may have to withdraw your manuscript. We cannot publish manuscripts where authorship has not been agreed by all authors (including those who have been removed).

- Incomplete forms will be rejected.

- Please return/upload this form, fully completed, to the Journals Editorial Office. The Journal and/or Publisher will consider the information you have provided to decide whether to approve the proposed change in authorship. We may decide to contact your institution for more information or undertake a further investigation, if appropriate, before making a final decision.

SPRINGER NATURE Springer Nature is one of the world's leading global research, educational and professional publishers, created in May 2015 through the combination of Nature Publishing Group, Palgrave Macmillan, Macmillan Education and Springer Science+Business Media.

Docusign Envelope ID: 08AC93CF-74A7-4761-BC72-D71E01F28E71

SPRINGER NATURE

Change of authorship request form - Journals (pre-acceptance)

Section 1: Please provide the current title of manuscript

Manuscript ID no.: bbe75767-4b88-4f30-901c-d461565b8ea7 v4.0

Title: Escaping the OR: A Pilot Study of a Jigsaw-Based Workshop to Teach Preoperative Assessment in Internal Medicine Residency

Section 2: Please provide the previous authorship, in the order shown on the manuscript before the changes were introduced. Please indicate the corresponding author by adding (CA) behind the name.

	First name(s)	Family name	ORCID or SCOPUS id, if available
1st author	Ruth	Abeles	
2nd author	Laura	Eger	
3rd author	Saloni	Maharaj	
4th author			
5th author			
6th author			
7th author			
8th author			
9th author			
10th author			

Please use an additional sheet if there are more than 10 authors.

SPRINGER NATURE Springer Nature is one of the world's leading global research, educational and professional publishers, created in May 2015 through the combination of Nature Publishing Group, Palgrave Macmillan, Macmillan Education and Springer Science+Business Media.

Docusign Envelope ID: 08AC93CF-74A7-4761-BC72-D71E01F28E71

Change of authorship request form - Journals (pre-acceptance)

Section 3: Please provide a justification for change. Please use this section to explain your reasons for changing the authorship of your manuscript, e.g. what necessitated the change in authorship? Please refer to the (journal) policy pages for more information about authorship. Please explain why omitted authors were not originally included and/or why authors were removed on the submitted manuscript.

We would like to inform the editorial team that additional authors have been added to the revised manuscript. At the time of the original submission, the manuscript reflected the contributions of the core pilot study team who were directly involved in the initial workshop implementation and preliminary data analysis.
During the revision process, the scope of the manuscript evolved substantially in response to reviewer feedback. The revisions required expanded methodological clarification, additional statistical interpretation (including effect size analysis), restructuring of the discussion to better address feasibility and generalizability, and refinement of the educational framework to enhance applicability beyond the pilot cohort.
The newly added authors made meaningful intellectual contributions to these revisions, including substantial input into data interpretation, manuscript restructuring, response-to-reviewer development, and critical revision of the content for important intellectual content. Their contributions meet standard authorship criteria, and all authors have reviewed and approved the final version of the manuscript.
We believe this expanded authorship appropriately reflects the collaborative effort required to strengthen and refine the manuscript during the revision process.

Section 4: Proposed new authorship. Please provide your new authorship list in the order you would like it to appear on the manuscript. Please indicate the corresponding author by adding (CA) behind the name. If the Corresponding Author has changed, please indicate the reason under section 3.

	First name(s)	Family name (this name will appear in full on the final publication and will be searchable in various abstract and indexing databases)	Text<br>Affiliated institute	E-mail address
1st author	Ruth	Abeles	University of California, San Diego	rabeles@health.ucsd.edu
2nd author	Laura	Eger	University of Califorinia, San Diego	lroudebush@health.ucsd.edu
3rd author	Tanner	Slayden	University of California, San Diego	tslayden@health.ucsd.edu
4th author	Stacy	Charat	University of California, San Diego	scharat@health.ucsd.edu
5th author	Saloni	Maharaj	Stanford University, School of Medicine	salonikumar@stanford.edu
6th author				
7th author				
8th author				
9th author				
10th author				

Please use an additional sheet if there are more than 10 authors.

Docusign Envelope ID: 08AC93CF-74A7-4761-BC72-D71E01F28E71

Change of authorship request form - Journals (pre-acceptance)

Section 5: Author contribution, Acknowledgement and Disclosures. Please use this section to provide a new disclosure statement and, if appropriate, acknowledge any contributors who have been removed as authors and ensure you state what contribution any new authors made (if applicable per the journal or book (series) policy). Please ensure these are updated in your manuscript - after approval of the change(s) - as our production department will not transfer the information in this form to your manuscript.

New acknowledgements:

Same as before

New Disclosures (financial and non-financial interests, funding):

none

New Author Contributions statement (if applicable per the journal policy):

RA conceived and designed the study, developed the educational intervention, facilitated the educational workshop, collected and analyzed the data, and drafted and revised the manuscript. LE performed the data analysis. SKM contributed to educational learning materials and manuscript revision. SC contributed to the organization and coordination of the primary care resident small groups, ensuring effective and efficient implementation of the preoperative Jigsaw pilot workshop. SC also assisted with manuscript revision. TS served as a faculty facilitator during the workshop and contributed to manuscript revision. All authors read and approved the final manuscript.

State ‘Not applicable’ if there are no new authors.

SPRINGER NATURE Springer Nature is one of the world’s leading global research, educational and professional publishers, created in May 2015 through the combination of Nature Publishing Group, Palgrave Macmillan, Macmillan Education and Springer Science+Business Media.

Docusign Envelope ID: 08AC93CF-74A7-4761-BC72-D71E01F28E71

SPRINGER NATURE

Change of authorship request form - Journals (pre-acceptance)

Section 6: Declaration of agreement. *All* authors, unchanged, new and removed *must* sign this declaration.
(NB: Please print the form, (docu)-sign and return/upload a scanned copy. Please note that signatures that have been inserted as an image file are acceptable as long as it is handwritten. Typed names in the signature box are unacceptable.) *** Please delete as appropriate. Delete all of the bold if you were on the original authorship list and are remaining as an author.**

	First name	Family name		Signature	Date
1st author	Ruth	Abeles	I agree to the proposed new authorship shown in section 4 **/and the addition/removal*of my name to the authorship list** /and the proposed change in corresponding author		2/17/2026
2nd author	Laura Eger		I agree to the proposed new authorship shown in section 4 **/and the addition/removal*of my name to the authorship list** /and the proposed change in corresponding author	Signed by: Laura Eger 0D15BFFF5072448...	2/18/2026
3rd author	Tanner Slayden		I agree to the proposed new authorship shown in section 4 **/and the addition/removal*of my name to the authorship list** /and the proposed change in corresponding author	Signed by: Tanner Slayden ED152EF02482492...	2/18/2026
4th authors	Stacy Charat		I agree to the proposed new authorship shown in section 4 **/and the addition/removal*of my** Text **name to the authorship list** /and the proposed change in corresponding author	Signed by: F412F6794BC94DD...	2/18/2026
5th author	Saloni Kumar Maharaj		I agree to the proposed new authorship shown in section 4 **/and the addition/removal*of my name to the authorship list** /and the proposed change in corresponding author	Signed by: Saloni Kumar Maharaj 50E4B2C9190341E...	2/18/2026
6th author			I agree to the proposed new authorship shown in section 4 **/and the addition/removal*of my name to the authorship list** /and the proposed change in corresponding author		
7th author			I agree to the proposed new authorship shown in section 4 **/and the addition/removal*of my name to the authorship list** /and the proposed change in corresponding author		

SPRINGER NATURE Springer Nature is one of the world's leading global research, educational and professional publishers, created in May 2015 through the combination of Nature Publishing Group, Palgrave Macmillan, Macmillan Education and Springer Science+Business Media.

Docusign Envelope ID: 08AC93CF-74A7-4761-BC72-D71E01F28E71

SPRINGER NATURE

Change of authorship request form - Journals (pre-acceptance)

	First name	Family name		Signature	Date
8th author			I agree to the proposed new authorship shown in section 4 /**and the addition/removal*of my name to the authorship list** /and the proposed change in corresponding author		
9th author			I agree to the proposed new authorship shown in section 4 /**and the addition/removal*of my name to the authorship list** /and the proposed change in corresponding author		
10th author			I agree to the proposed new authorship shown in section 4 /**and the addition/removal*of my name to the authorship list** /and the proposed change in corresponding author		

Please use an additional sheet if there are more than 10 authors.

In case of author collaborations with formal agreement:

	Name of consortium/consortia	First name	Family name		Signature	Date
Representative/legal guarantor				I agree to the proposed new authorship shown in section 4 /**and the addition/removal*of my name to the authorship list** /and the proposed change in corresponding author		

Both added/removed authors should complete the information in the first table under Section 6.

---- End of form ----

SPRINGER NATURE Springer Nature is one of the world's leading global research, educational and professional publishers, created in May 2015 through the combination of Nature Publishing Group, Palgrave Macmillan, Macmillan Education and Springer Science+Business Media.
