# Supplementary material for: Escaping the OR: a pilot study of a Jigsaw-based workshop to teach preoperative assessment in internal medicine residency
Source: BMC Med Educ. 2026 May 16;26:1097. doi: 10.1186/s12909-026-09419-w (PMC13348809; doi:10.1186/s12909-026-09419-w)
Supplement: Supplementary file 1 — Supplementary Material 1. [file 12909_2026_9419_MOESM1_ESM.zip › Manuscript Figures Final 2.0.docx]

Figure 1: In the control group (n=11 pre, n=10 post), notable gains are observed in special population (40% to 49%) and anticoagulation (41.1% to 48.50%). Cardiovascular risk and medical management remain stable (45.40% to 39.40% and 60.60% to 56.1% respectively). In the intervention group (n=11, n=10 post), all categories show notable gains: cardiovascular risk group (37.50% to 66.70%), medication management (64.5% to 75%), special population (45% to 95%), and anticoagulation (45.80% to 58.30%).

Figure 2: In the control group (n=11 pre, n = 10 post), changes in mean confidence scores were generally small and less consistent. In over half of the categories, post-test confidence scores were lower than pre-test scores. In the intervention group (n=8 pre, n=8 post) mean confidence scores showed consistent gains in confidence across all domains following the educational session.
