# Supplementary material for: Escaping the OR: a pilot study of a Jigsaw-based workshop to teach preoperative assessment in internal medicine residency
Source: BMC Med Educ. 2026 May 16;26:1097. doi: 10.1186/s12909-026-09419-w (PMC13348809; doi:10.1186/s12909-026-09419-w)
Supplement: Supplementary file 1 — Supplementary Material 1. [file 12909_2026_9419_MOESM1_ESM.zip › Post control.pdf]

# POST Control Pre Op Group / Page 1

**Responses:** 14

Age: 14

**Age:**

30

28

31

28

28

29

29

30

29

Pooja Pandita

31

29

28

## Gender 14

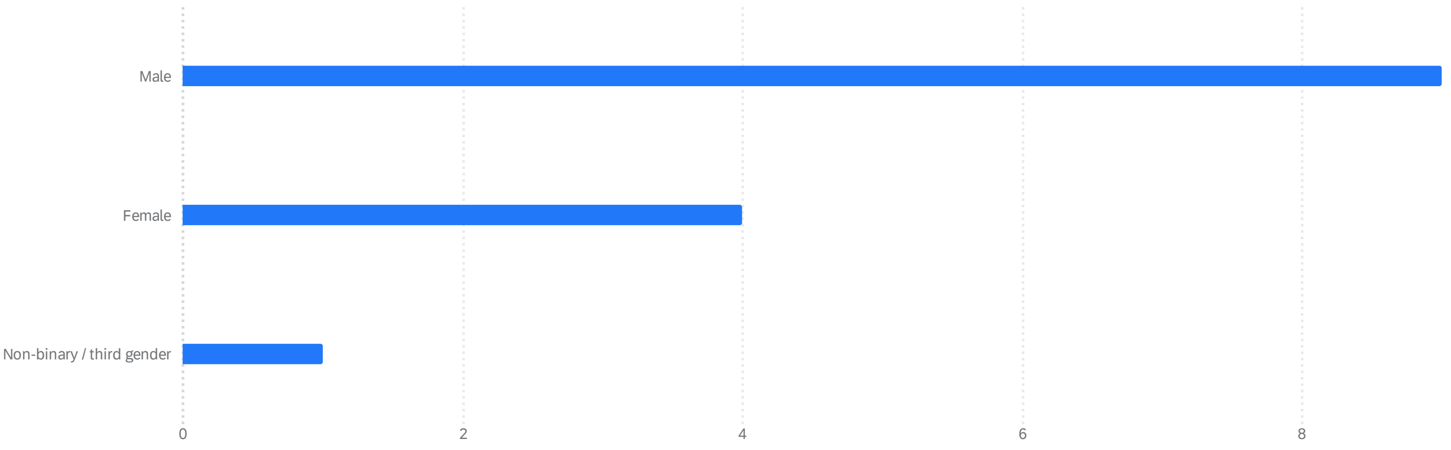


## Gender 14

| Q2 - Gender | Count | Count |
|---|---|---|
| Male | 64% | 9 |
| Female | 29% | 4 |
| Non-binary / third gender | 7% | 1 |

## Gender 14

| Q2 - Gender | Average (Q2 - Gender) | Minimum (Q2 - Gender) | Maximum (Q2 - Gender) | Count |
|---|---|---|---|---|
| Female | 2.00 | 2.00 | 2.00 | 4 |
| Male | 1.00 | 1.00 | 1.00 | 9 |
| Non-binary / third gender | 3.00 | 3.00 | 3.00 | 1 |

## Ethnicity (Check all that apply): 14

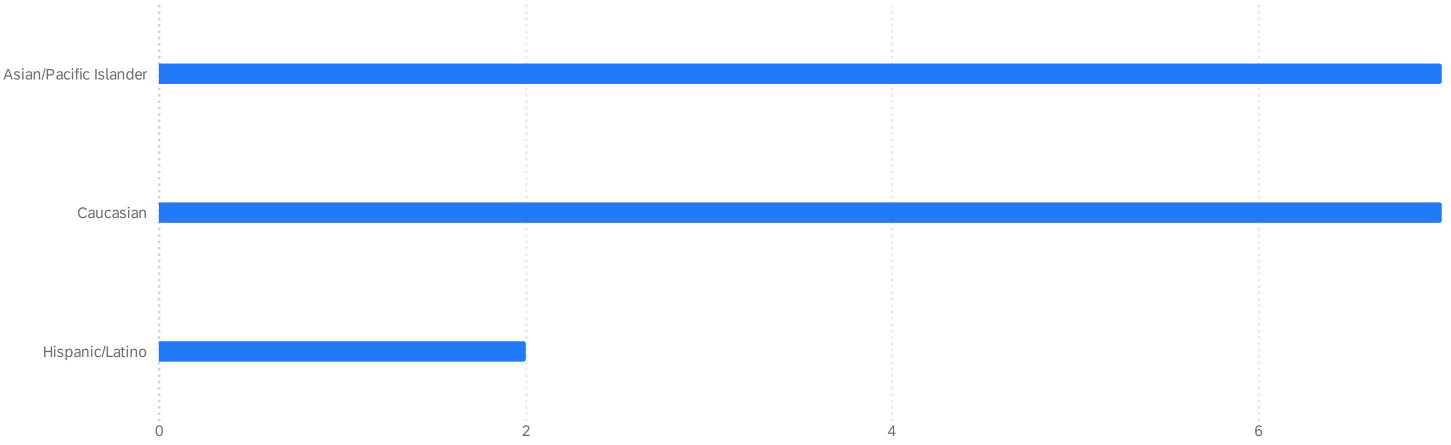

Ethnicity (Check all that apply): 14

| Q3 - Ethnicity (Check all that apply): | Count | Count |
|---|---|---|
| Asian/Pacific Islander | 50% | 7 |
| Caucasian | 50% | 7 |
| Hispanic/Latino | 14% | 2 |

Please indicate your PGY year: 14

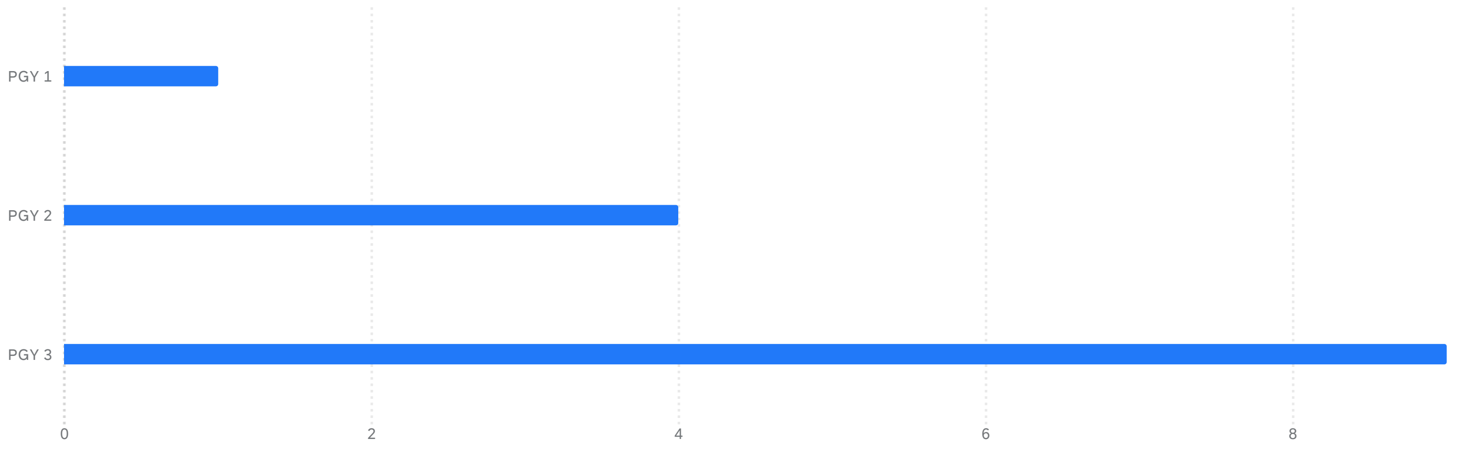


Please indicate your PGY year: 14

| Q4 - Please indicate your PGY year: | Count | Count |
|---|---|---|
| PGY 1 | 7% | 1 |
| PGY 2 | 29% | 4 |
| PGY 3 | 64% | 9 |

Please indicate your PGY year: 14

| Q4 - Please indicate your PGY year: | Average (Q4 - Please indicate your PGY year:) | Minimum (Q4 - Please indicate your PGY year:) | Maximum (Q4 - Please indicate your PGY year:) | Count |
|---|---|---|---|---|
| PGY 1 | 1.00 | 1.00 | 1.00 | 1 |
| PGY 2 | 2.00 | 2.00 | 2.00 | 4 |
| PGY 3 | 3.00 | 3.00 | 3.00 | 9 |

Have you received training in ambulatory preoperative assessment in the following settings? 14

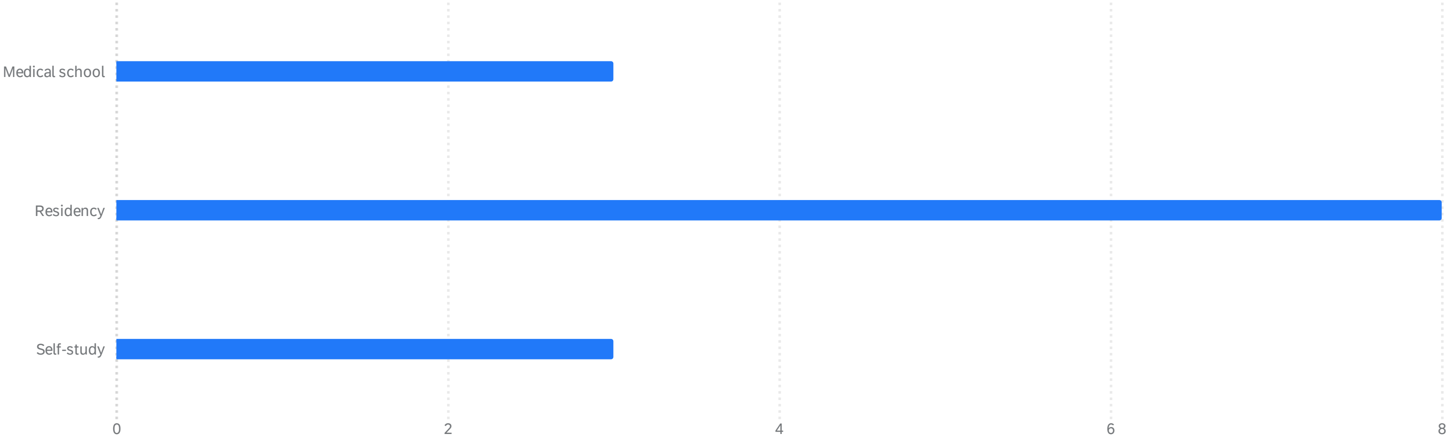


Have you received training in ambulatory preoperative assessment in the following settings? 14

| Q5 - Have you received training in ambulatory preoperative assessment in the following settings? | Count | Count |
|---|---|---|
| Medical school | 21% | 3 |
| Residency | 57% | 8 |
| Self-study | 21% | 3 |

Have you received training in ambulatory preoperative assessment in the following settings? 14

| Q5 - Have you received training in ambulatory preoperative assessment in the following settings? | Average (Q5 - Have you received training in ambulatory preoperative assessment in the following settings?) | Minimum (Q5 - Have you received training in ambulatory preoperative assessment in the following settings?) | Maximum (Q5 - Have you received training in ambulatory preoperative assessment in the following settings?) | Count |
|---|---|---|---|---|
| Medical school | 2.00 | 2.00 | 2.00 | 3 |
| Residency | 4.00 | 4.00 | 4.00 | 8 |
| Self-study | 5.00 | 5.00 | 5.00 | 3 |

Have you received training in inpatient preoperative assessment in the following settings? 14

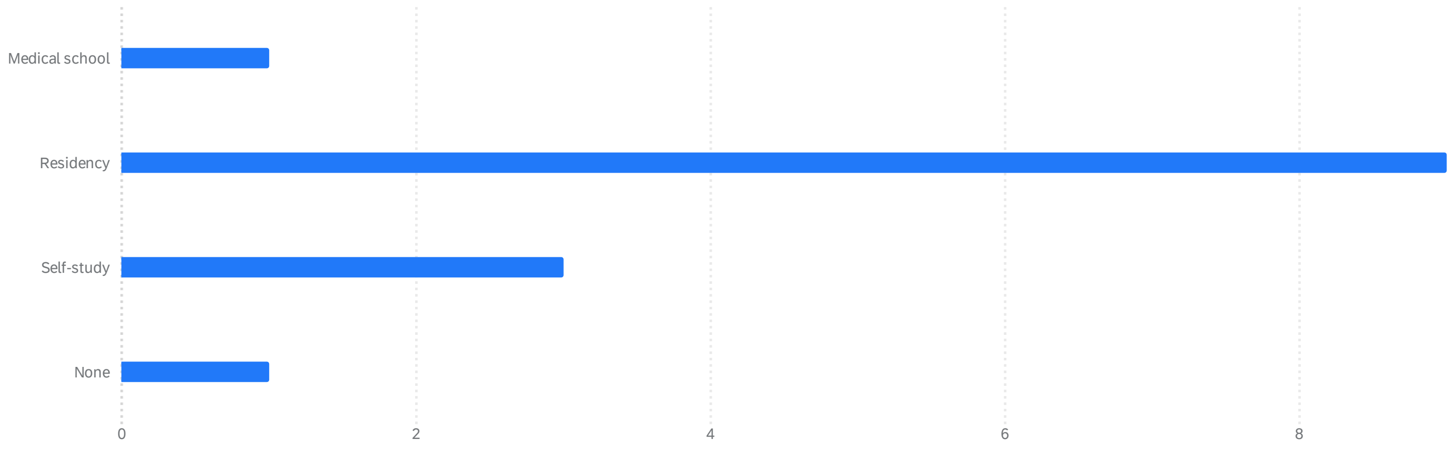


Have you received training in inpatient preoperative assessment in the following settings? 14

| Q6 - Have you received training in inpatient preoperative assessment in the following settings? | Count | Count |
|---|---|---|
| Medical school | 7% | 1 |
| Residency | 64% | 9 |
| Self-study | 21% | 3 |
| None | 7% | 1 |

Have you received training in inpatient preoperative assessment in the following settings? 14

| Q6 - Have you received training in inpatient preoperative assessment in the following settings? | Average (Q6 - Have you received training in inpatient preoperative assessment in the following settings?) | Minimum (Q6 - Have you received training in inpatient preoperative assessment in the following settings?) | Maximum (Q6 - Have you received training in inpatient preoperative assessment in the following settings?) | Count |
|---|---|---|---|---|
| Medical school | 2.00 | 2.00 | 2.00 | 1 |
| None | 6.00 | 6.00 | 6.00 | 1 |
| Residency | 4.00 | 4.00 | 4.00 | 9 |
| Self-study | 5.00 | 5.00 | 5.00 | 3 |

How confident are you in performing ambulatory preoperative assessment? 14

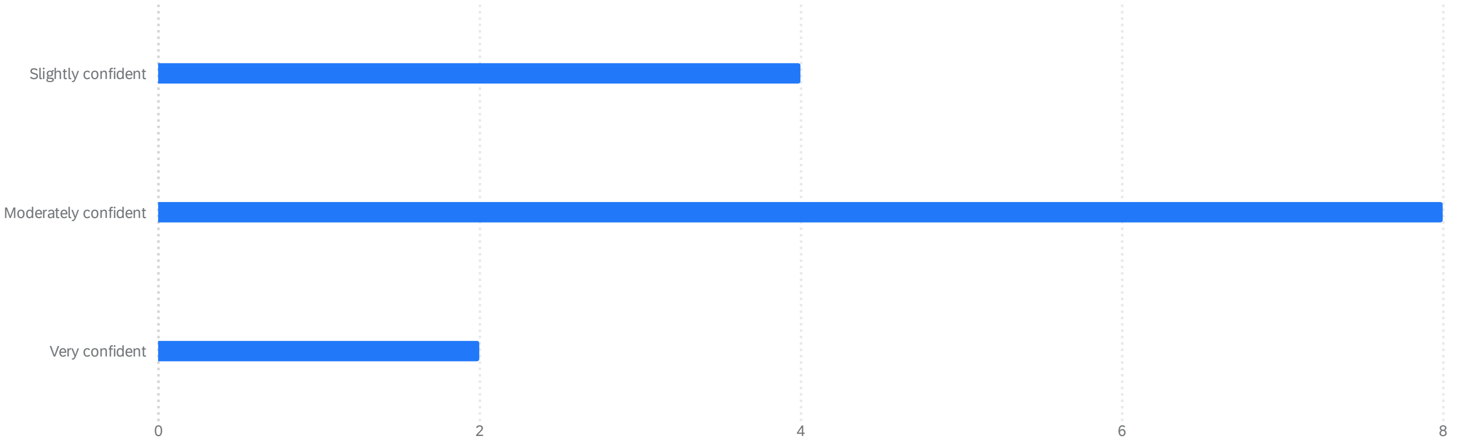

How confident are you in performing ambulatory preoperative assessment? 14

| Q7 - How confident are you in performing ambulatory preoperative assessment? | Count | Count |
| --- | --- | --- |
| Slightly confident | 29% | 4 |
| Moderately confident | 57% | 8 |
| Very confident | 14% | 2 |

How confident are you in performing ambulatory preoperative assessment? 14

| Q7 - How confident are you in performing ambulatory preoperative assessment? | Average (Q7 - How confident are you in performing ambulatory preoperative assessment?) | Minimum (Q7 - How confident are you in performing ambulatory preoperative assessment?) | Maximum (Q7 - How confident are you in performing ambulatory preoperative assessment?) | Count |
| --- | --- | --- | --- | --- |
| Moderately confident | 3.00 | 3.00 | 3.00 | 8 |
| Slightly confident | 2.00 | 2.00 | 2.00 | 4 |
| Very confident | 4.00 | 4.00 | 4.00 | 2 |

How confident are you in performing inpatient preoperative assessment? 14

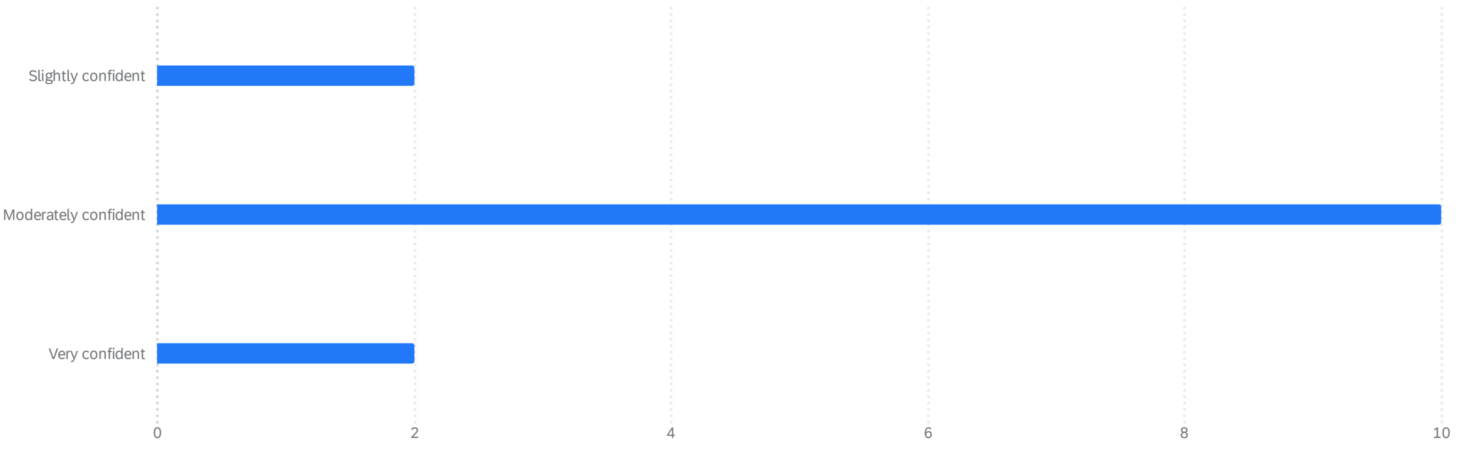


How confident are you in performing inpatient preoperative assessment? 14

| Q8 - How confident are you in performing inpatient preoperative assessment? | Count | Count |
| --- | --- | --- |
| Slightly confident | 14% | 2 |
| Moderately confident | 71% | 10 |
| Very confident | 14% | 2 |

How confident are you in performing inpatient preoperative assessment? 14

| Q8 - How confident are you in performing inpatient preoperative assessment? | Average (Q8 - How confident are you in performing inpatient preoperative assessment?) | Minimum (Q8 - How confident are you in performing inpatient preoperative assessment?) | Maximum (Q8 - How confident are you in performing inpatient preoperative assessment?) | Count |
|---|---|---|---|---|
| Moderately confident | 3.00 | 3.00 | 3.00 | 10 |
| Slightly confident | 2.00 | 2.00 | 2.00 | 2 |
| Very confident | 4.00 | 4.00 | 4.00 | 2 |

How confident are you in applying the ACC/AHA guidelines for perioperative cardiovascular evaluation? 14

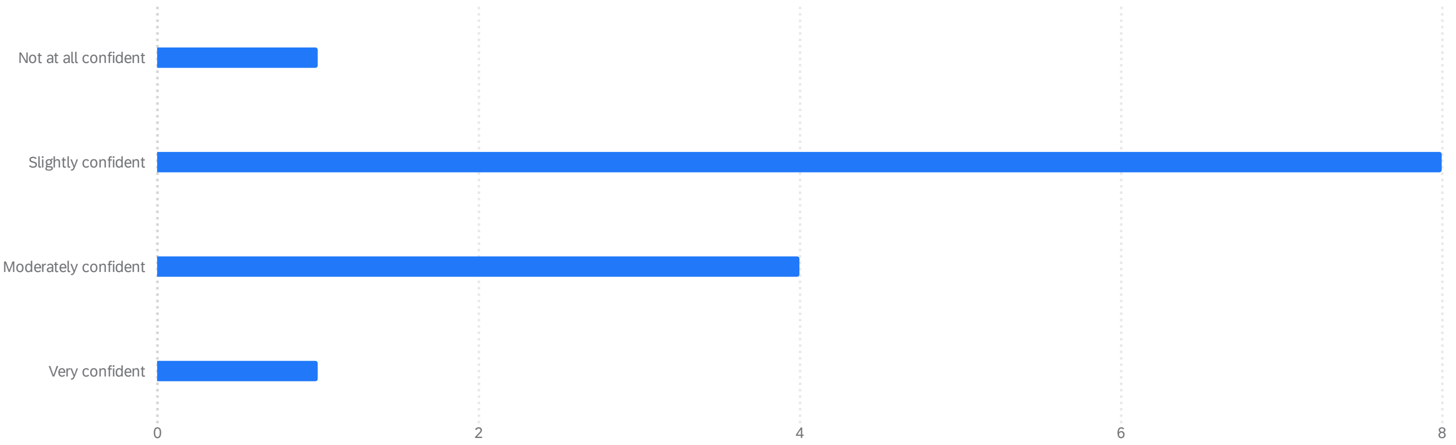


How confident are you in applying the ACC/AHA guidelines for perioperative cardiovascular evaluation? 14

| Q9 - How confident are you in applying the ACC/AHA guidelines for perioperative cardiovascular evaluation? | Count | Count |
|---|---|---|
| Not at all confident | 7% | 1 |
| Slightly confident | 57% | 8 |
| Moderately confident | 29% | 4 |
| Very confident | 7% | 1 |

How confident are you in applying the ACC/AHA guidelines for perioperative cardiovascular evaluation? 14

| Q9 - How confident are you in applying the ACC/AHA guidelines for perioperative cardiovascular evaluation? | Average (Q9 - How confident are you in applying the ACC/AHA guidelines for perioperative cardiovascular evaluation?) | Minimum (Q9 - How confident are you in applying the ACC/AHA guidelines for perioperative cardiovascular evaluation?) | Maximum (Q9 - How confident are you in applying the ACC/AHA guidelines for perioperative cardiovascular evaluation?) | Count |
|---|---|---|---|---|
| Moderately confident | 3.00 | 3.00 | 3.00 | 4 |
| Not at all confident | 1.00 | 1.00 | 1.00 | 1 |
| Slightly confident | 2.00 | 2.00 | 2.00 | 8 |
| Very confident | 4.00 | 4.00 | 4.00 | 1 |

How confident are you in managing patients undergoing bariatric surgery preoperatively? 14

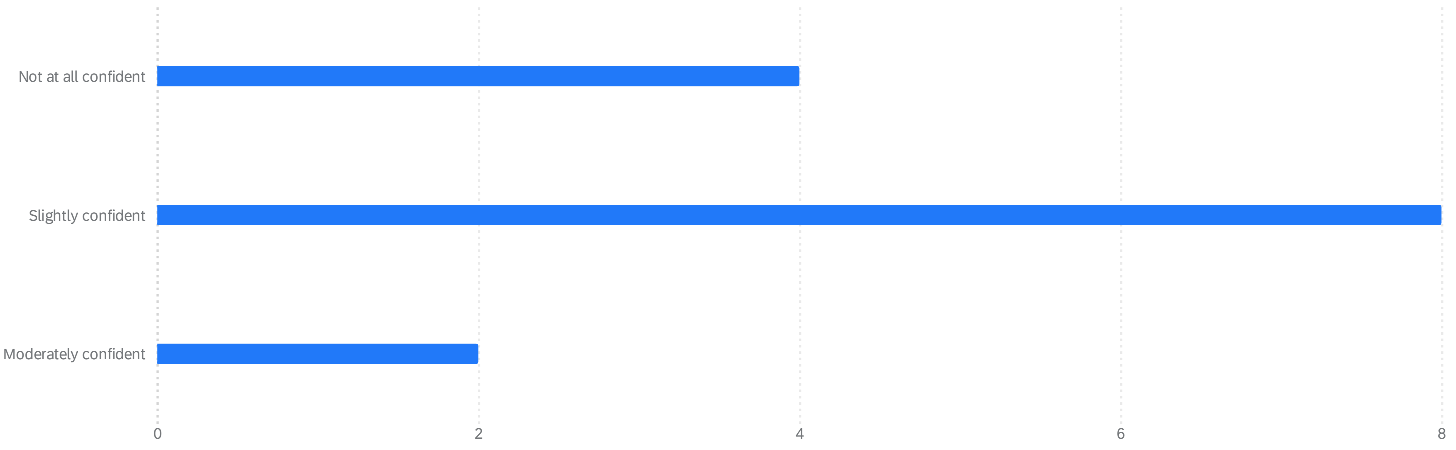


How confident are you in managing patients undergoing bariatric surgery preoperatively? 14

| Q10 - How confident are you in managing patients undergoing bariatric surgery preoperatively? | Count | Count |
|---|---|---|
| Not at all confident | 29% | 4 |
| Slightly confident | 57% | 8 |
| Moderately confident | 14% | 2 |

How confident are you in managing patients undergoing bariatric surgery preoperatively? 14

| Q10 - How confident are you in managing patients undergoing bariatric surgery preoperatively? | Average (Q10 - How confident are you in managing patients undergoing bariatric surgery preoperatively?) | Minimum (Q10 - How confident are you in managing patients undergoing bariatric surgery preoperatively?) | Maximum (Q10 - How confident are you in managing patients undergoing bariatric surgery preoperatively?) | Count |
|---|---|---|---|---|
| Moderately confident | 3.00 | 3.00 | 3.00 | 2 |
| Not at all confident | 1.00 | 1.00 | 1.00 | 4 |
| Slightly confident | 2.00 | 2.00 | 2.00 | 8 |

How confident are you in managing patients with chronic kidney disease preoperatively? 14

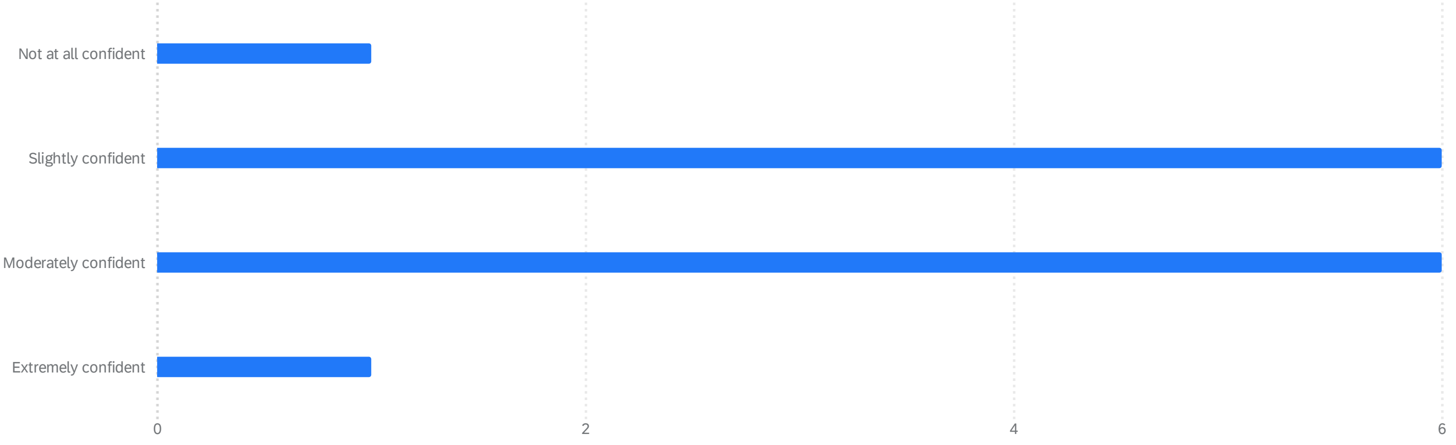


How confident are you in managing patients with chronic kidney disease preoperatively? 14

| Q11 - How confident are you in managing patients with chronic kidney disease preoperatively? | Count | Count |
|---|---|---|
| Not at all confident | 7% | 1 |
| Slightly confident | 43% | 6 |
| Moderately confident | 43% | 6 |
| Extremely confident | 7% | 1 |

How confident are you in managing patients with chronic kidney disease preoperatively? 14

| Q11 - How confident are you in managing patients with chronic kidney disease preoperatively? | Average (Q11 - How confident are you in managing patients with chronic kidney disease preoperatively?) | Minimum (Q11 - How confident are you in managing patients with chronic kidney disease preoperatively?) | Maximum (Q11 - How confident are you in managing patients with chronic kidney disease preoperatively?) | Count |
|---|---|---|---|---|
| Extremely confident | 5.00 | 5.00 | 5.00 | 1 |
| Moderately confident | 3.00 | 3.00 | 3.00 | 6 |
| Not at all confident | 1.00 | 1.00 | 1.00 | 1 |
| Slightly confident | 2.00 | 2.00 | 2.00 | 6 |

How confident are you in managing patients with liver disease preoperatively? 14

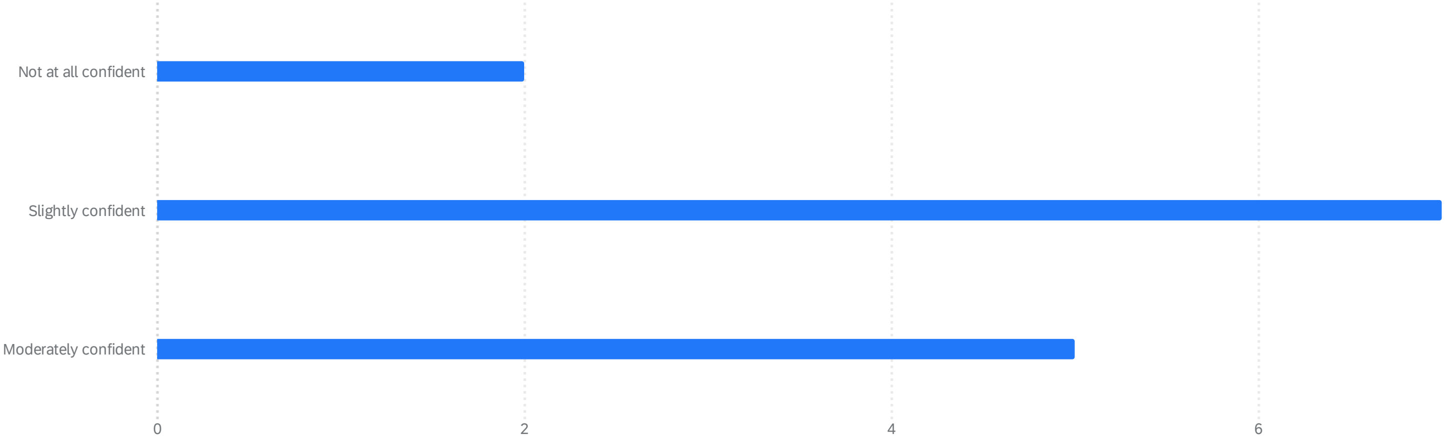

How confident are you in managing patients with liver disease preoperatively? 14

| Q12 - How confident are you in managing patients with liver disease preoperatively? | Count | Count |
|---|---|---|
| Not at all confident | 14% | 2 |
| Slightly confident | 50% | 7 |
| Moderately confident | 36% | 5 |

How confident are you in managing patients with liver disease preoperatively? 14

| Q12 - How confident are you in managing patients with liver disease preoperatively? | Average (Q12 - How confident are you in managing patients with liver disease preoperatively?) | Minimum (Q12 - How confident are you in managing patients with liver disease preoperatively?) | Maximum (Q12 - How confident are you in managing patients with liver disease preoperatively?) | Count |
|---|---|---|---|---|
| Moderately confident | 3.00 | 3.00 | 3.00 | 5 |
| Not at all confident | 1.00 | 1.00 | 1.00 | 2 |
| Slightly confident | 2.00 | 2.00 | 2.00 | 7 |

How confident are you in managing patients with rheumatologic conditions (e.g., RA) preoperatively? 14

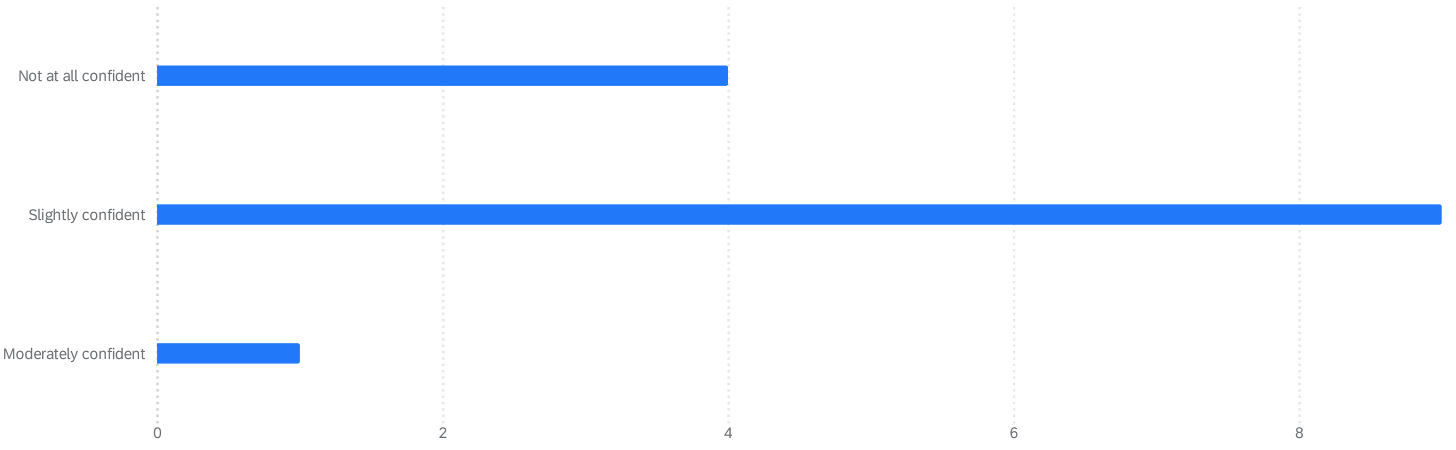


How confident are you in managing patients with rheumatologic conditions (e.g., RA) preoperatively? 14

| Q13 - How confident are you in managing patients with rheumatologic conditions (e.g., RA) preoperatively? | Count | Count |
|---|---|---|
| Not at all confident | 29% | 4 |
| Slightly confident | 64% | 9 |
| Moderately confident | 7% | 1 |

How confident are you in managing patients with rheumatologic conditions (e.g., RA) preoperatively? 14

| Q13 - How confident are you in managing patients with rheumatologic conditions (e.g., RA) preoperatively? | Average (Q13 - How confident are you in managing patients with rheumatologic conditions (e.g., RA) preoperatively?) | Minimum (Q13 - How confident are you in managing patients with rheumatologic conditions (e.g., RA) preoperatively?) | Maximum (Q13 - How confident are you in managing patients with rheumatologic conditions (e.g., RA) preoperatively?) | Count |
|---|---|---|---|---|
| Moderately confident | 3.00 | 3.00 | 3.00 | 1 |
| Not at all confident | 1.00 | 1.00 | 1.00 | 4 |
| Slightly confident | 2.00 | 2.00 | 2.00 | 9 |

Guidelines recommend utilizing risk calculators to estimate risk of major adverse cardiac events (MACE). What is the percentage cut off? 10

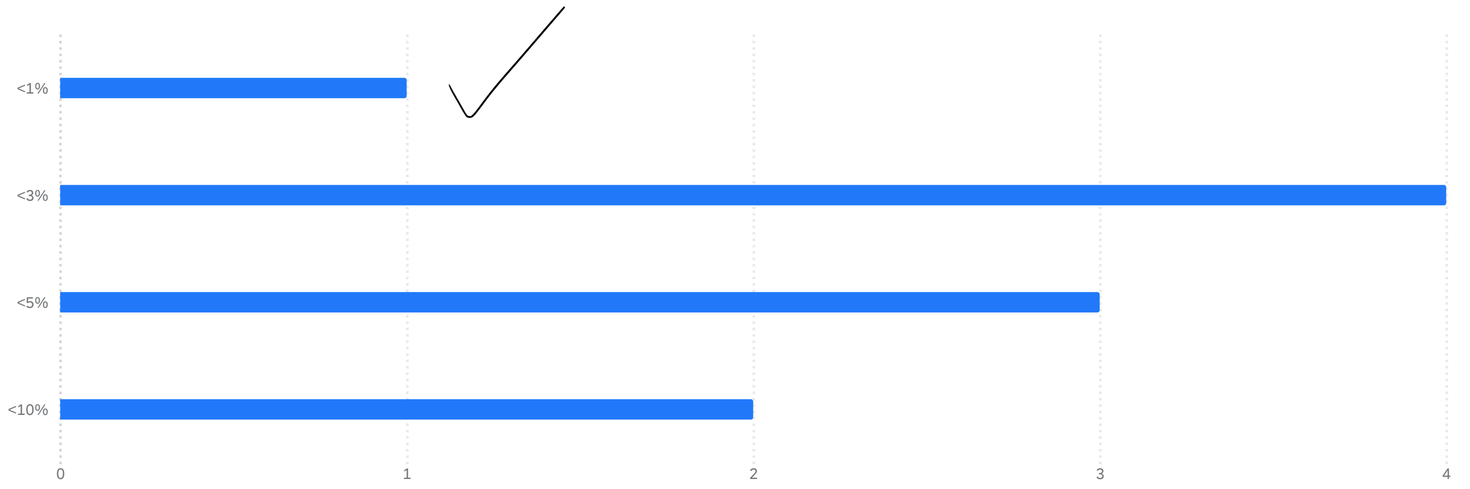


Guidelines recommend utilizing risk calculators to estimate risk of major adverse cardiac events (MACE). What is the percentage cut off? 10

| Q14 - Guidelines recommend utilizing risk calculators to estimate risk of major adverse cardiac events (MACE). What is the percentage cut off? | Count | Count |
|---|---|---|
| <1% | 10% | 1 |
| <3% | 40% | 4 |
| <5% | 30% | 3 |
| <10% | 20% | 2 |

Guidelines recommend utilizing risk calculators to estimate risk of major adverse cardiac events (MACE). What is the percentage cut off? 10

| Q14 - Guidelines recommend utilizing risk calculators to estimate risk of major adverse cardiac events (MACE). What is the percentage cut off? | Average (Q14 - Guidelines recommend utilizing risk calculators to estimate risk of major adverse cardiac events (MACE). What is the percentage cut off?) | Minimum (Q14 - Guidelines recommend utilizing risk calculators to estimate risk of major adverse cardiac events (MACE). What is the percentage cut off?) | Maximum (Q14 - Guidelines recommend utilizing risk calculators to estimate risk of major adverse cardiac events (MACE). What is the percentage cut off?) | Count |
|---|---|---|---|---|
| <1% | 1.00 | 1.00 | 1.00 | 1 |
| <10% | 4.00 | 4.00 | 4.00 | 2 |
| <3% | 2.00 | 2.00 | 2.00 | 4 |
| <5% | 3.00 | 3.00 | 3.00 | 3 |

For patients who received bare metal stents, which of the following is a TRUE statement? 10

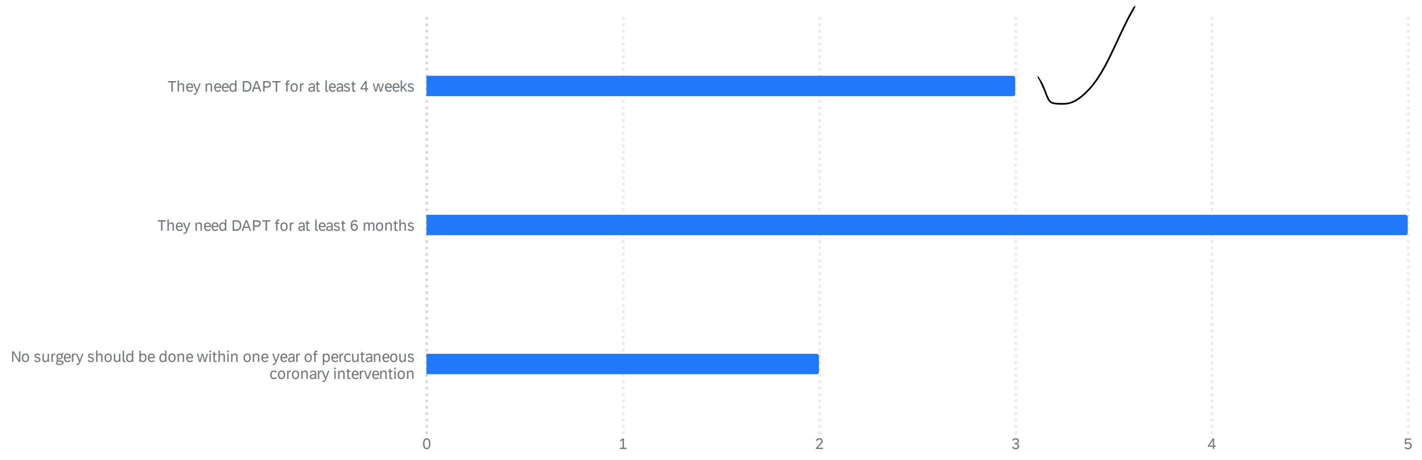


For patients who received bare metal stents, which of the following is a TRUE statement? 10

| Q15 - For patients who received bare metal stents, which of the following is a TRUE statement? | Count | Count |
|---|---|---|
| They need DAPT for at least 4 weeks | 30% | 3 |
| They need DAPT for at least 6 months | 50% | 5 |
| No surgery should be done within one year of percutaneous coronary intervention | 20% | 2 |

For patients who received bare metal stents, which of the following is a TRUE statement? 10

| Q15 - For patients who received bare metal stents, which of the following is a TRUE statement? | Average (Q15 - For patients who received bare metal stents, which of the following is a TRUE statement?) | Minimum (Q15 - For patients who received bare metal stents, which of the following is a TRUE statement?) | Maximum (Q15 - For patients who received bare metal stents, which of the following is a TRUE statement?) | Count |
|---|---|---|---|---|
| No surgery should be done within one year of percutaneous coronary intervention | 3.00 | 3.00 | 3.00 | 2 |
| They need DAPT for at least 4 weeks | 1.00 | 1.00 | 1.00 | 3 |
| They need DAPT for at least 6 months | 2.00 | 2.00 | 2.00 | 5 |

For patients who received drug-eluting stents, what is the recommended duration for dual anti-platelet therapy? 10

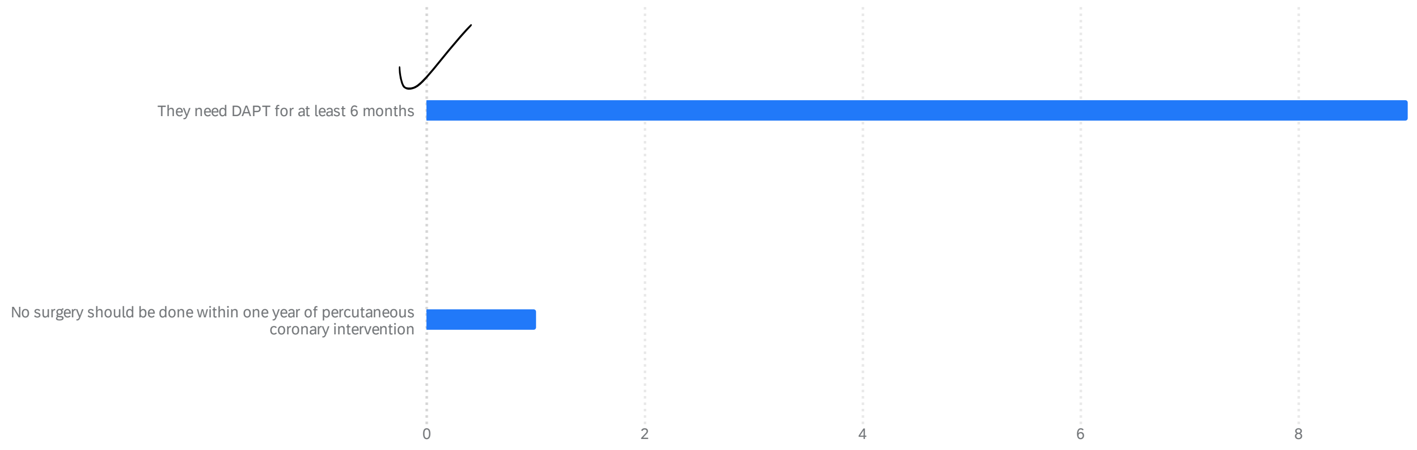


For patients who received drug-eluting stents, what is the recommended duration for dual anti-platelet therapy? 10

| Q16 - For patients who received drug-eluting stents, what is the recommended duration for dual anti-platelet therapy? | Count | Count |
|---|---|---|
| They need DAPT for at least 6 months | 90% | 9 |
| No surgery should be done within one year of percutaneous coronary intervention | 10% | 1 |

For patients who received drug-eluting stents, what is the recommended duration for dual anti-platelet therapy? 10

| Q16 - For patients who received drug-eluting stents, what is the recommended duration for dual anti-platelet therapy? | Average (Q16 - For patients who received drug-eluting stents, what is the recommended duration for dual anti-platelet therapy?) | Minimum (Q16 - For patients who received drug-eluting stents, what is the recommended duration for dual anti-platelet therapy?) | Maximum (Q16 - For patients who received drug-eluting stents, what is the recommended duration for dual anti-platelet therapy?) | Count |
|---|---|---|---|---|
| No surgery should be done within one year of percutaneous coronary intervention | 3.00 | 3.00 | 3.00 | 1 |
| They need DAPT for at least 6 months | 2.00 | 2.00 | 2.00 | 9 |

Which statement is true? 10

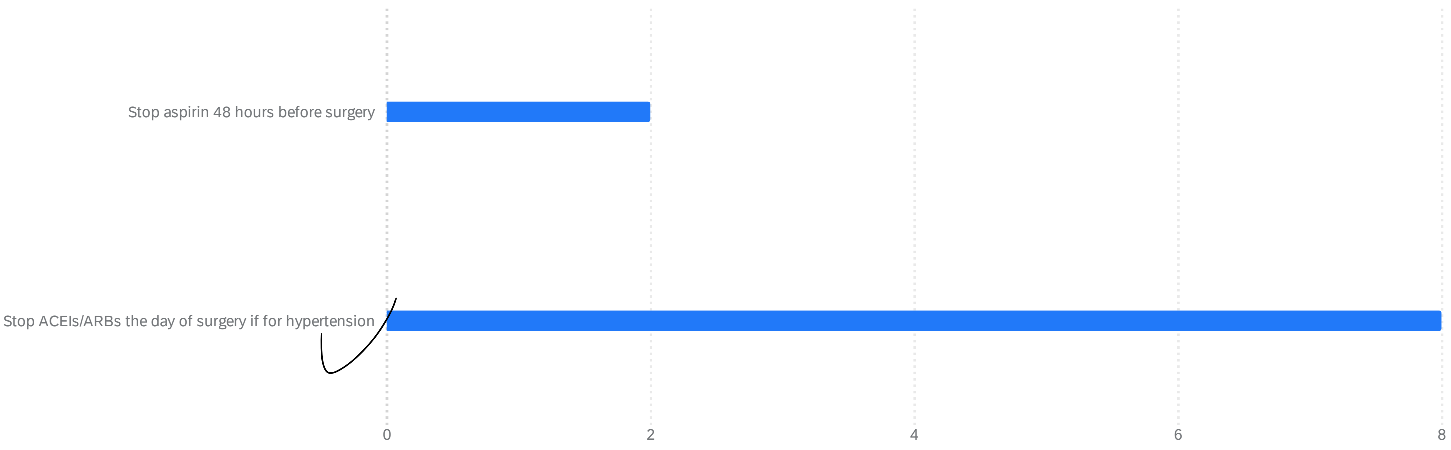

## Which statement is true? 10

| Q17 - Which statement is true? | Count | Count |
|---|---|---|
| Stop aspirin 48 hours before surgery | 20% | 2 |
| Stop ACEIs/ARBs the day of surgery if for hypertension | 80% | 8 |

## Which statement is true? 10

| Q17 - Which statement is true? | Average (Q17 - Which statement is true?) | Minimum (Q17 - Which statement is true?) | Maximum (Q17 - Which statement is true?) | Count |
|---|---|---|---|---|
| Stop ACEIs/ARBs the day of surgery if for hypertension | 3.00 | 3.00 | 3.00 | 8 |
| Stop aspirin 48 hours before surgery | 1.00 | 1.00 | 1.00 | 2 |

## Which set of patients require stress dose steroids to prevent adrenal crisis during surgery? 10

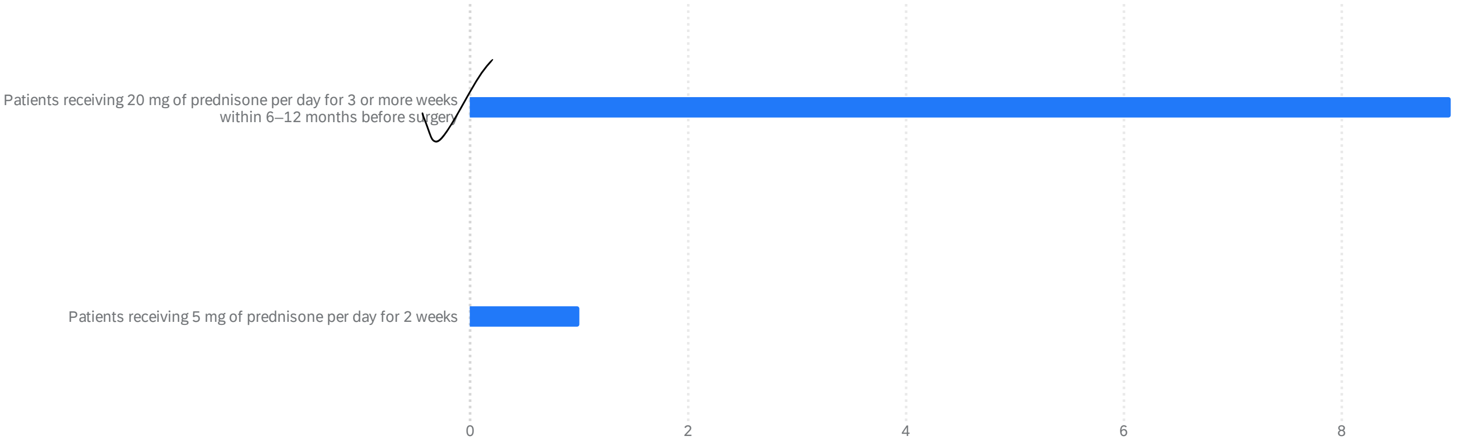


## Which set of patients require stress dose steroids to prevent adrenal crisis during surgery? 10

| Q18 - Which set of patients require stress dose steroids to prevent adrenal crisis during surgery? | Count | Count |
|---|---|---|
| Patients receiving 20 mg of prednisone per day for 3 or more weeks within 6–12 months before surgery | 90% | 9 |
| Patients receiving 5 mg of prednisone per day for 2 weeks | 10% | 1 |

## Which set of patients require stress dose steroids to prevent adrenal crisis during surgery? 10

| Q18 - Which set of patients require stress dose steroids to prevent adrenal crisis during surgery? | Average (Q18 - Which set of patients require stress dose steroids to prevent adrenal crisis during surgery?) | Minimum (Q18 - Which set of patients require stress dose steroids to prevent adrenal crisis during surgery?) | Maximum (Q18 - Which set of patients require stress dose steroids to prevent adrenal crisis during surgery?) | Count |
|---|---|---|---|---|
| Patients receiving 20 mg of prednisone per day for 3 or more weeks within 6–12 months before surgery | 2.00 | 2.00 | 2.00 | 9 |

| Q18 - Which set of patients require stress dose steroids to prevent adrenal crisis during surgery? | Average (Q18 - Which set of patients require stress dose steroids to prevent adrenal crisis during surgery?) | Minimum (Q18 - Which set of patients require stress dose steroids to prevent adrenal crisis during surgery?) | Maximum (Q18 - Which set of patients require stress dose steroids to prevent adrenal crisis during surgery?) | Count |
|---|---|---|---|---|
| Patients receiving 5 mg of prednisone per day for 2 weeks | 3.00 | 3.00 | 3.00 | 1 |

Which of the following statements are true regarding insulin management prior to surgery for patients with type I diabetes? (Select all that apply) 10

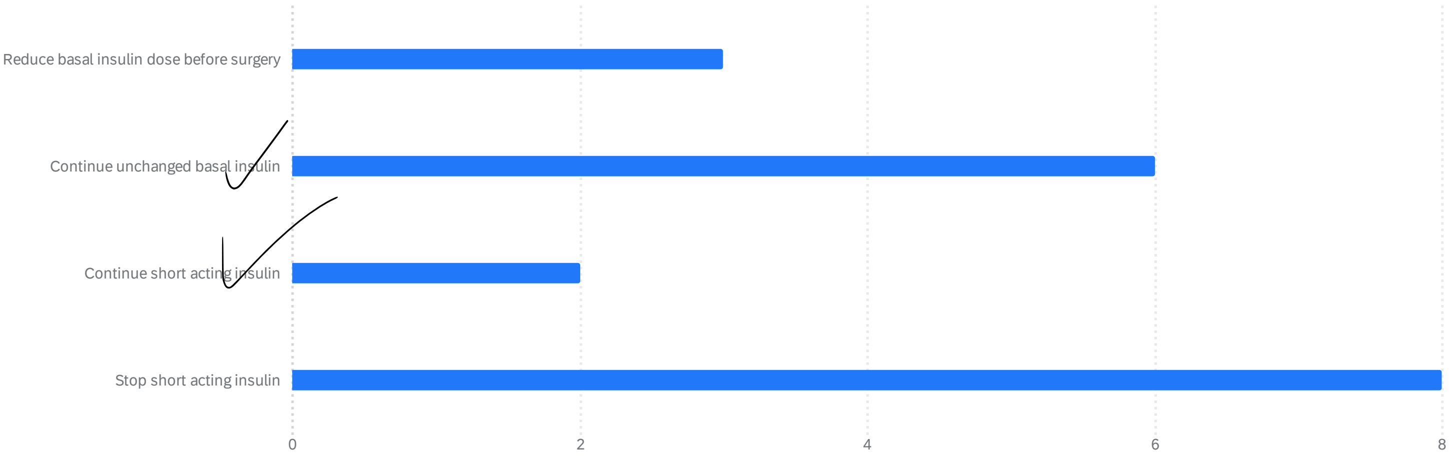


Which of the following statements are true regarding insulin management prior to surgery for patients with type I diabetes? (Select all that apply) 10

| Q19 - Which of the following statements are true regarding insulin management prior to surgery for patients with type I diabetes? (Select all that apply) | Count | Count |
|---|---|---|
| Reduce basal insulin dose before surgery | 30% | 3 |
| Continue unchanged basal insulin | 60% | 6 |
| Continue short acting insulin | 20% | 2 |
| Stop short acting insulin | 80% | 8 |

Which statement regarding SGLT2 inhibitors is true? (Select all that apply) 10

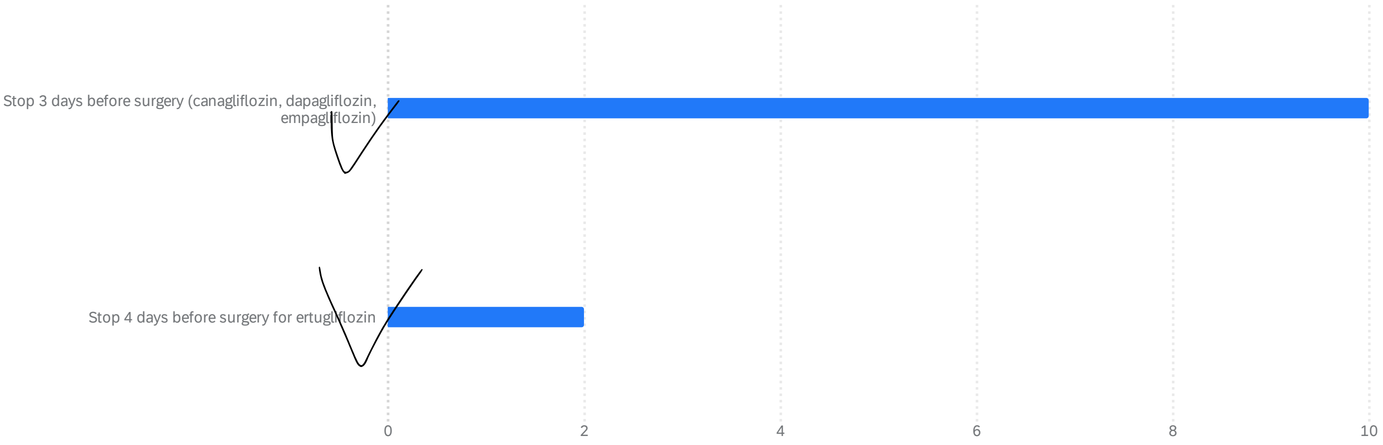

Which statement regarding SGLT2 inhibitors is true? (Select all that apply) 10

| Q20 - Which statement regarding SGLT2 inhibitors is true? (Select all that apply) | Count | Count |
|---|---|---|
| Stop 3 days before surgery (canagliflozin, dapagliflozin, empagliflozin) | 100% | 10 |
| Stop 4 days before surgery for ertugliflozin | 20% | 2 |

Which rheumatologic agents are safe to continue before surgery? (Select all that apply) 10

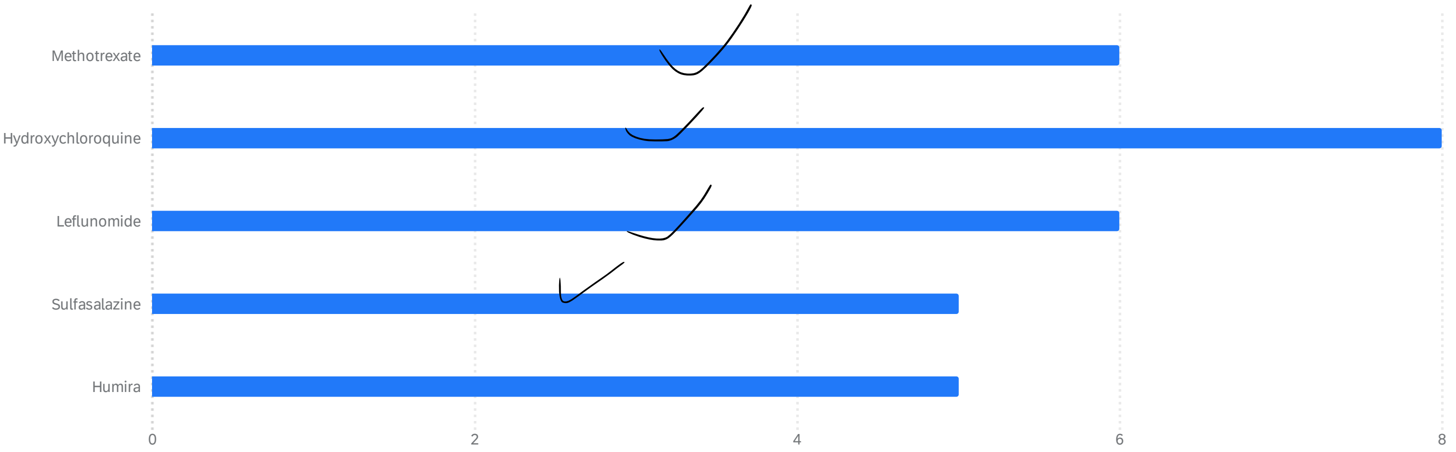


Which rheumatologic agents are safe to continue before surgery? (Select all that apply) 10

| Q21 - Which rheumatologic agents are safe to continue before surgery? (Select all that apply) | Count | Count |
|---|---|---|
| Methotrexate | 60% | 6 |
| Hydroxychloroquine | 80% | 8 |
| Leflunomide | 60% | 6 |
| Sulfasalazine | 50% | 5 |
| Humira | 50% | 5 |

Which statement regarding bridging is true? (Select all that apply) 10

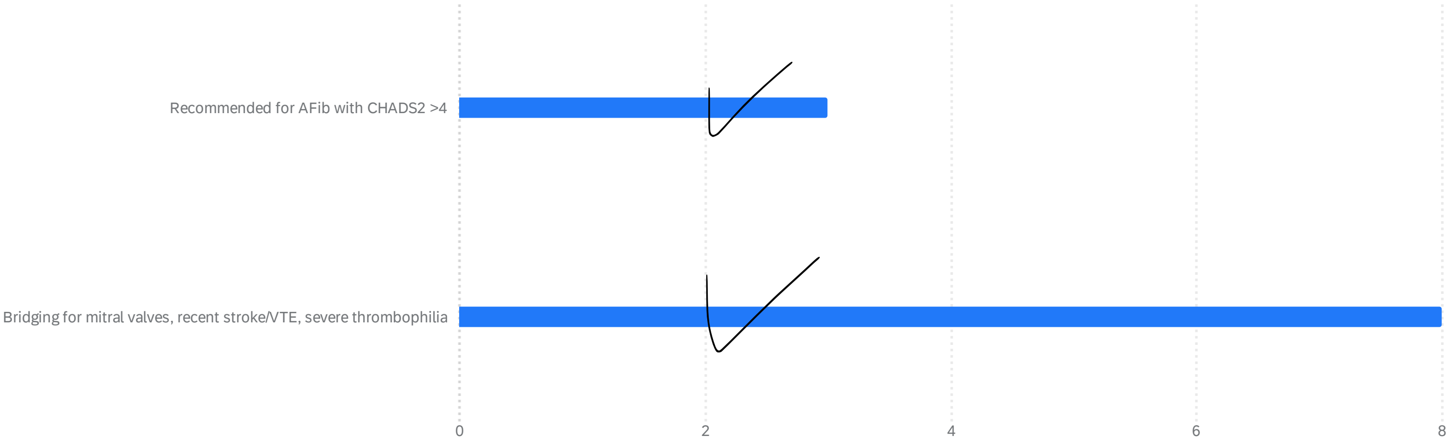

Which statement regarding bridging is true? (Select all that apply) 10

| Q22 - Which statement regarding bridging is true? (Select all that apply) | Count | Count |
|---|---|---|
| Recommended for AFib with CHADS2 >4 | 30% | 3 |
| Bridging for mitral valves, recent stroke/VTE, severe thrombophilia | 80% | 8 |

For patients on warfarin, you would check INR 7–10 days before the procedure. Which of the following is TRUE? (Select all that apply) 10

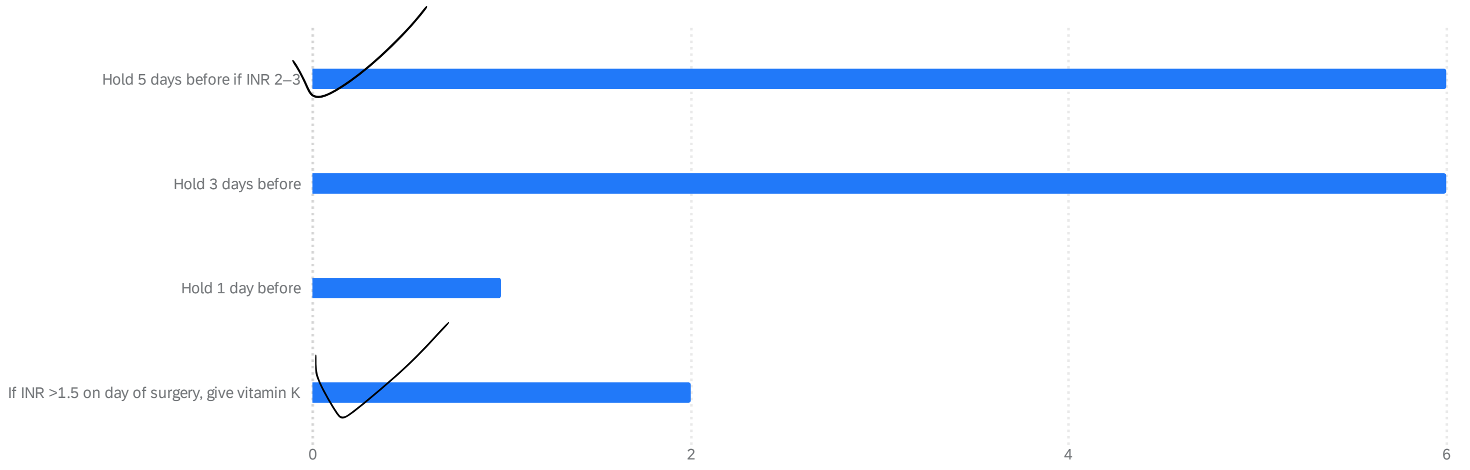


For patients on warfarin, you would check INR 7–10 days before the procedure. Which of the following is TRUE? (Select all that apply) 10

| Q23 - For patients on warfarin, you would check INR 7–10 days before the procedure. Which of the following is TRUE? (Select all that apply) | Count | Count |
|---|---|---|
| Hold 5 days before if INR 2–3 | 60% | 6 |
| Hold 3 days before | 60% | 6 |
| Hold 1 day before | 10% | 1 |
| If INR >1.5 on day of surgery, give vitamin K | 20% | 2 |

How do you restart warfarin after low-moderate bleeding risk procedure? 10

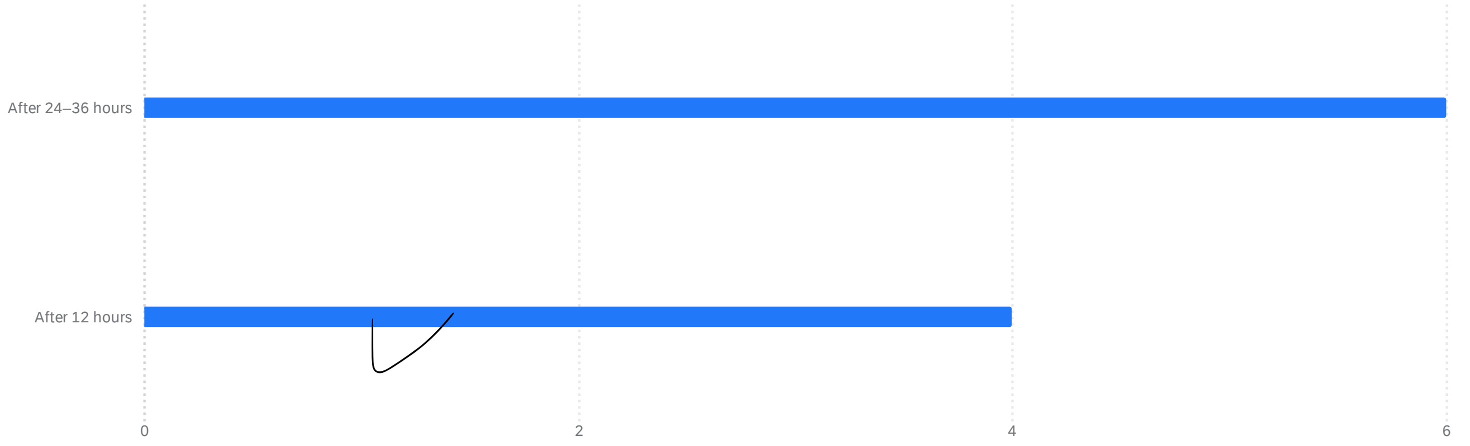

How do you restart warfarin after low-moderate bleeding risk procedure? 10

| Q24 - How do you restart warfarin after low-moderate bleeding risk procedure? | Count | Count |
|---|---|---|
| After 24–36 hours | 60% | 6 |
| After 12 hours | 40% | 4 |

How do you restart warfarin after low-moderate bleeding risk procedure? 10

| Q24 - How do you restart warfarin after low-moderate bleeding risk procedure? | Average (Q24 - How do you restart warfarin after low-moderate bleeding risk procedure?) | Minimum (Q24 - How do you restart warfarin after low-moderate bleeding risk procedure?) | Maximum (Q24 - How do you restart warfarin after low-moderate bleeding risk procedure?) | Count |
|---|---|---|---|---|
| After 12 hours | 2.00 | 2.00 | 2.00 | 4 |
| After 24–36 hours | 1.00 | 1.00 | 1.00 | 6 |

Which is true regarding DOACs and normal CrCl? 10

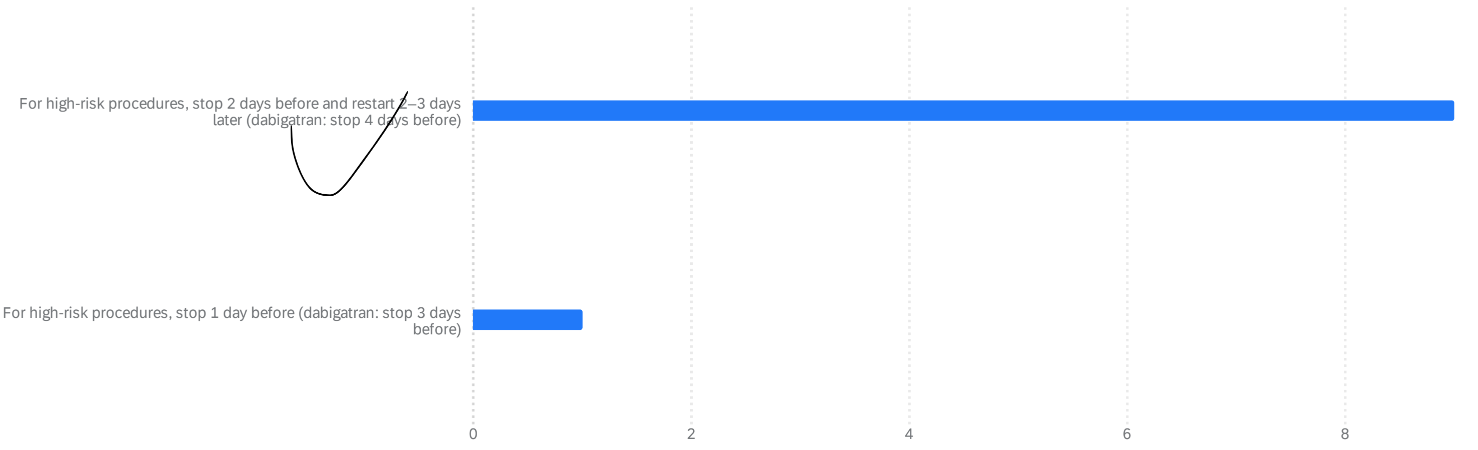


Which is true regarding DOACs and normal CrCl? 10

| Q25 - Which is true regarding DOACs and normal CrCl? | Count | Count |
|---|---|---|
| For high-risk procedures, stop 2 days before and restart 2–3 days later (dabigatran: stop 4 days before) | 90% | 9 |
| For high-risk procedures, stop 1 day before (dabigatran: stop 3 days before) | 10% | 1 |

Which is true regarding DOACs and normal CrCl? 10

| Q25 - Which is true regarding DOACs and normal CrCl? | Average (Q25 - Which is true regarding DOACs and normal CrCl?) | Minimum (Q25 - Which is true regarding DOACs and normal CrCl?) | Maximum (Q25 - Which is true regarding DOACs and normal CrCl?) | Count |
|---|---|---|---|---|
| For high-risk procedures, stop 1 day before (dabigatran: stop 3 days before) | 2.00 | 2.00 | 2.00 | 1 |
| For high-risk procedures, stop 2 days before and restart 2–3 days later (dabigatran: stop 4 days before) | 1.00 | 1.00 | 1.00 | 9 |

What calculator(s) can be used to assess VTE risk in bariatric surgery patients? (Select all that apply) 9

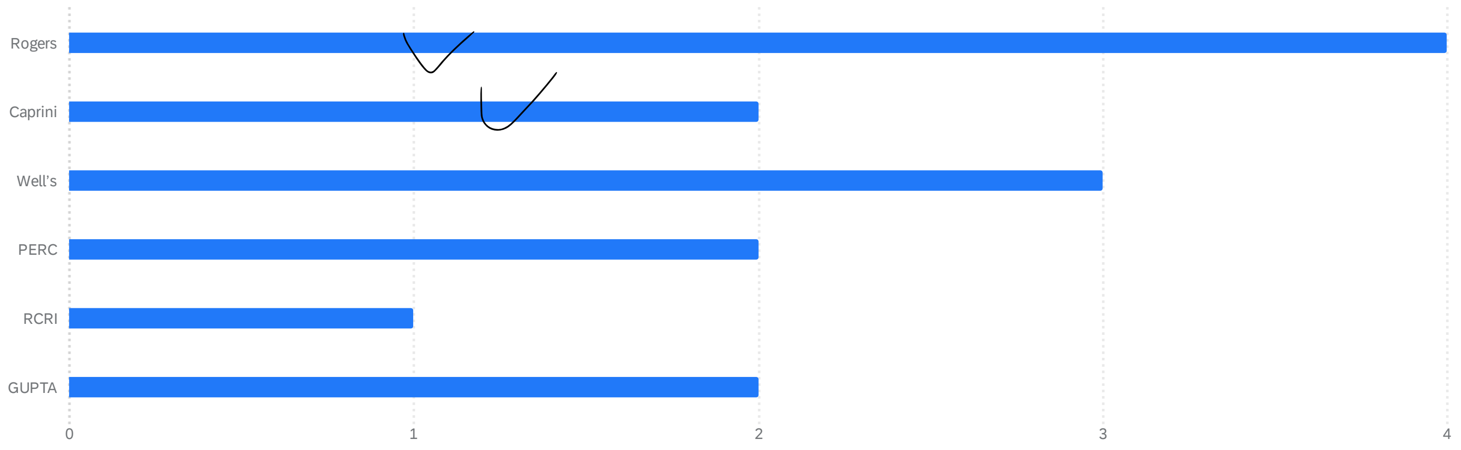


What calculator(s) can be used to assess VTE risk in bariatric surgery patients? (Select all that apply) 9

| Q26 - What calculator(s) can be used to assess VTE risk in bariatric surgery patients? (Select all that apply) | Count | Count |
|---|---|---|
| Rogers | 44% | 4 |
| Caprini | 22% | 2 |
| Well's | 33% | 3 |
| PERC | 22% | 2 |
| RCRI | 11% | 1 |
| GUPTA | 22% | 2 |

Please provide feedback for improvement: 14

**Please provide feedback for improvement:**
