# Supplementary material for: Escaping the OR: a pilot study of a Jigsaw-based workshop to teach preoperative assessment in internal medicine residency
Source: BMC Med Educ. 2026 May 16;26:1097. doi: 10.1186/s12909-026-09419-w (PMC13348809; doi:10.1186/s12909-026-09419-w)
Supplement: Supplementary file 1 — Supplementary Material 1. [file 12909_2026_9419_MOESM1_ESM.zip › Post intervention.pdf]

Post Intervention Pre OP Group Survey / Page 1

Responses: 7

Age: 7

Age:

27
32
34
30
39

Gender 5

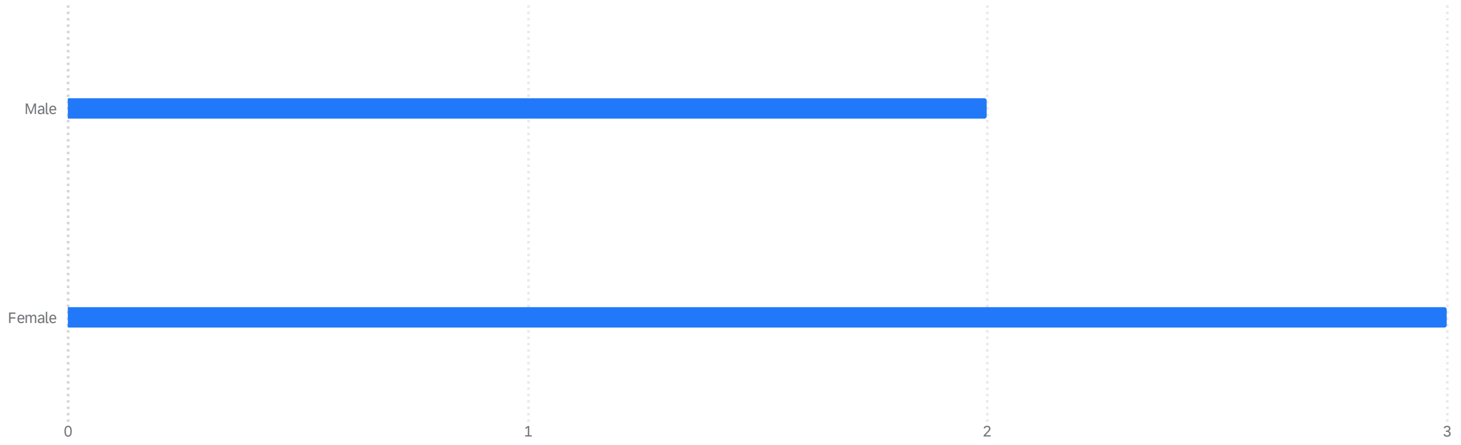

Gender 5

Q2 - Gender	Count	Count
Male	40%	2
Female	60%	3

Gender 5

Q2 - Gender	Average (Q2 - Gender)	Minimum (Q2 - Gender)	Maximum (Q2 - Gender)	Count
Female	2.00	2.00	2.00	3
Male	1.00	1.00	1.00	2

Ethnicity (Check all that apply): 5

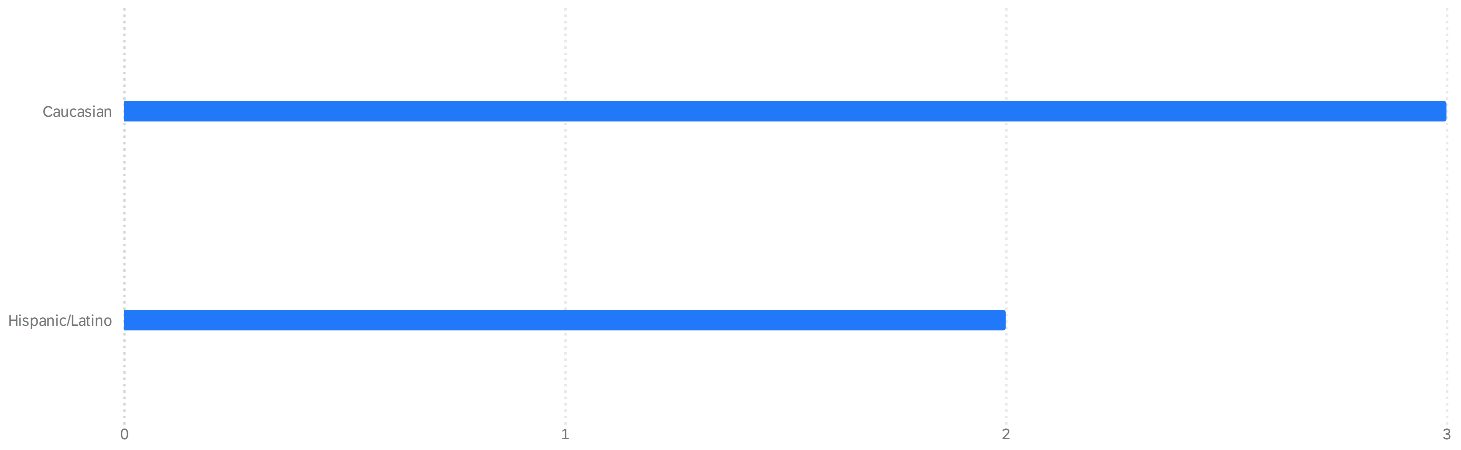

Ethnicity (Check all that apply): 5

Q3 - Ethnicity (Check all that apply):	Count	Count
Caucasian	60%	3
Hispanic/Latino	40%	2

Please indicate your PGY year: 5

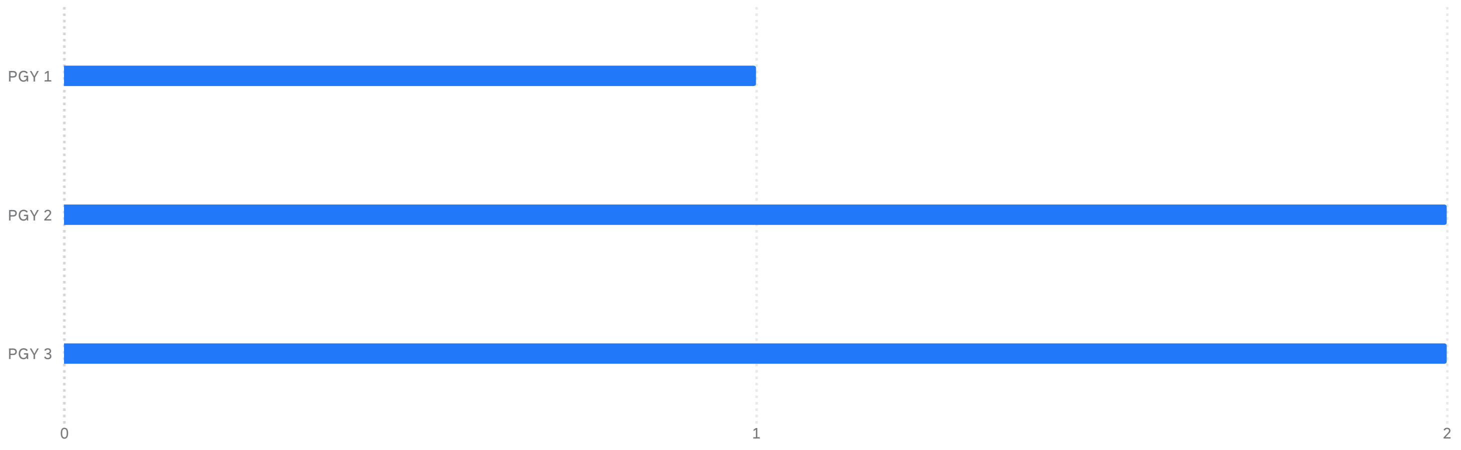

Please indicate your PGY year: 5

Q4 - Please indicate your PGY year:	Count	Count
PGY 1	20%	1
PGY 2	40%	2
PGY 3	40%	2

Please indicate your PGY year: 5

Q4 - Please indicate your PGY year:	Average (Q4 - Please indicate your PGY year:)	Minimum (Q4 - Please indicate your PGY year:)	Maximum (Q4 - Please indicate your PGY year:)	Count
PGY 1	1.00	1.00	1.00	1
PGY 2	2.00	2.00	2.00	2
PGY 3	3.00	3.00	3.00	2

Have you received training in ambulatory preoperative assessment in the following settings? 5

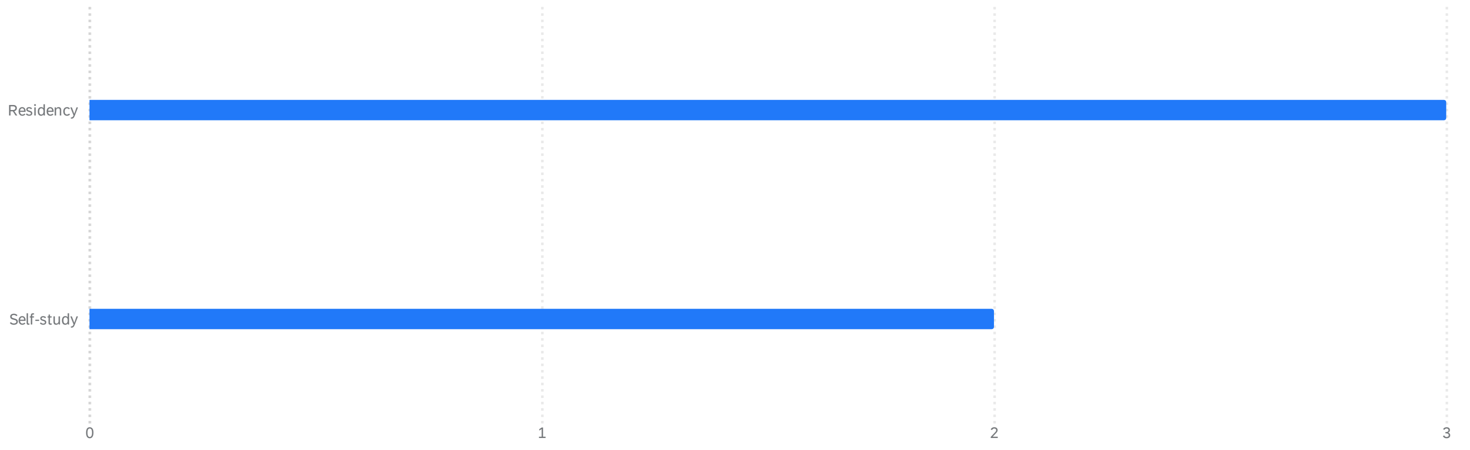

Have you received training in ambulatory preoperative assessment in the following settings? 5

Q5 - Have you received training in ambulatory preoperative assessment in the following settings?	Count	Count
Residency	60%	3
Self-study	40%	2

Have you received training in ambulatory preoperative assessment in the following settings? 5

Q5 - Have you received training in ambulatory preoperative assessment in the following settings?	Average (Q5 - Have you received training in ambulatory preoperative assessment in the following settings?)	Minimum (Q5 - Have you received training in ambulatory preoperative assessment in the following settings?)	Maximum (Q5 - Have you received training in ambulatory preoperative assessment in the following settings?)	Count
Residency	4.00	4.00	4.00	3
Self-study	5.00	5.00	5.00	2

Have you received training in inpatient preoperative assessment in the following settings? 5

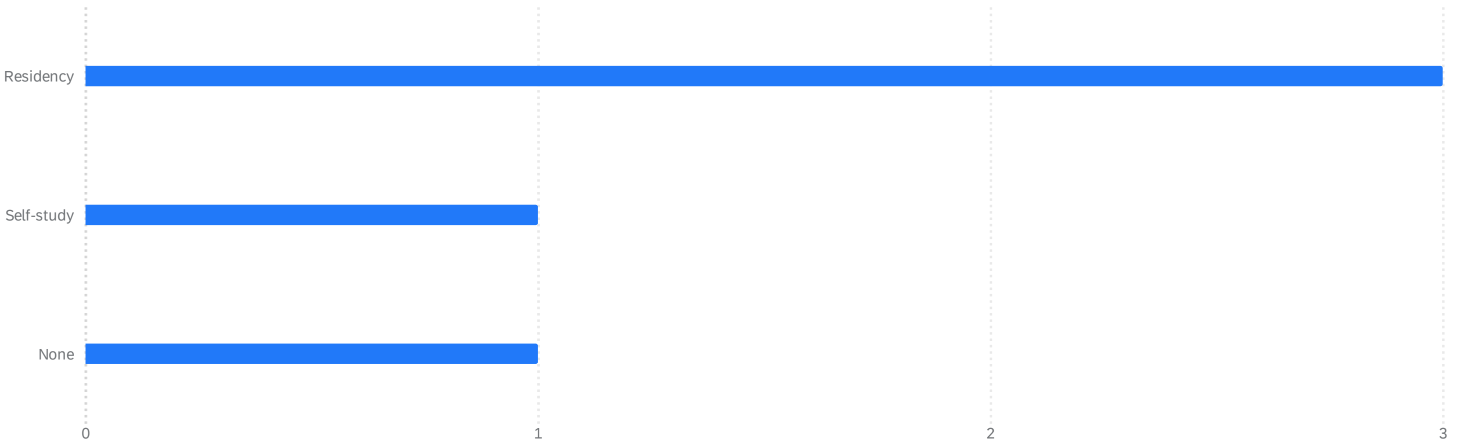

Have you received training in inpatient preoperative assessment in the following settings? 5

Q6 - Have you received training in inpatient preoperative assessment in the following settings?	Count	Count
Residency	60%	3
Self-study	20%	1
None	20%	1

Have you received training in inpatient preoperative assessment in the following settings? 5

Q6 - Have you received training in inpatient preoperative assessment in the following settings?	Average (Q6 - Have you received training in inpatient preoperative assessment in the following settings?)	Minimum (Q6 - Have you received training in inpatient preoperative assessment in the following settings?)	Maximum (Q6 - Have you received training in inpatient preoperative assessment in the following settings?)	Count
None	6.00	6.00	6.00	1
Residency	4.00	4.00	4.00	3
Self-study	5.00	5.00	5.00	1

How confident are you in performing ambulatory preoperative assessment? 6

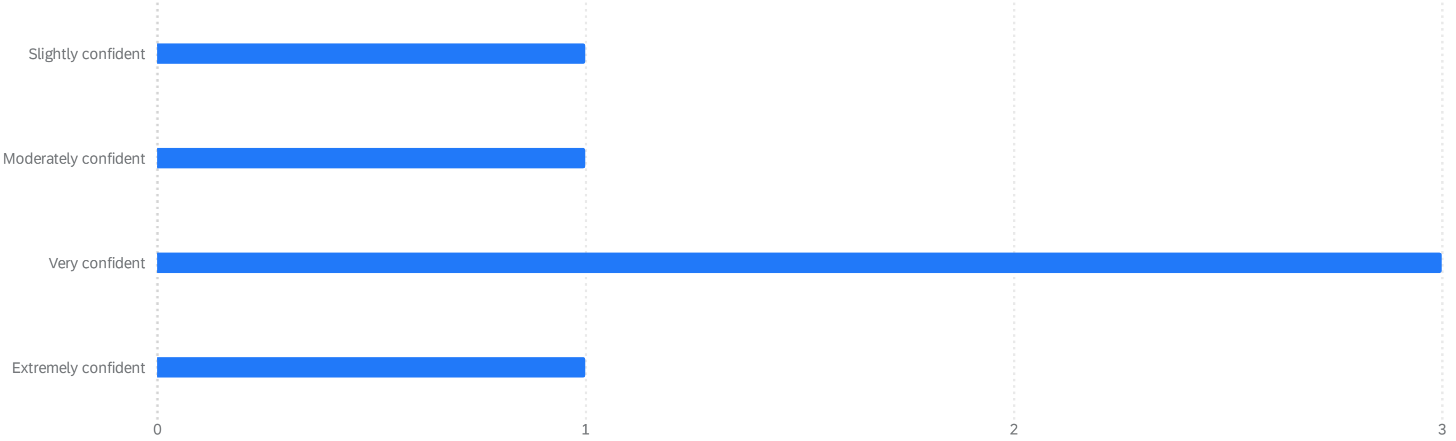

How confident are you in performing ambulatory preoperative assessment? 6

Q7 - How confident are you in performing ambulatory preoperative assessment?	Count	Count
Slightly confident	17%	1
Moderately confident	17%	1
Very confident	50%	3
Extremely confident	17%	1

How confident are you in performing ambulatory preoperative assessment? 6

Q7 - How confident are you in performing ambulatory preoperative assessment?	Average (Q7 - How confident are you in performing ambulatory preoperative assessment?)	Minimum (Q7 - How confident are you in performing ambulatory preoperative assessment?)	Maximum (Q7 - How confident are you in performing ambulatory preoperative assessment?)	Count
Extremely confident	5.00	5.00	5.00	1
Moderately confident	3.00	3.00	3.00	1
Slightly confident	2.00	2.00	2.00	1
Very confident	4.00	4.00	4.00	3

How confident are you in performing inpatient preoperative assessment? 5

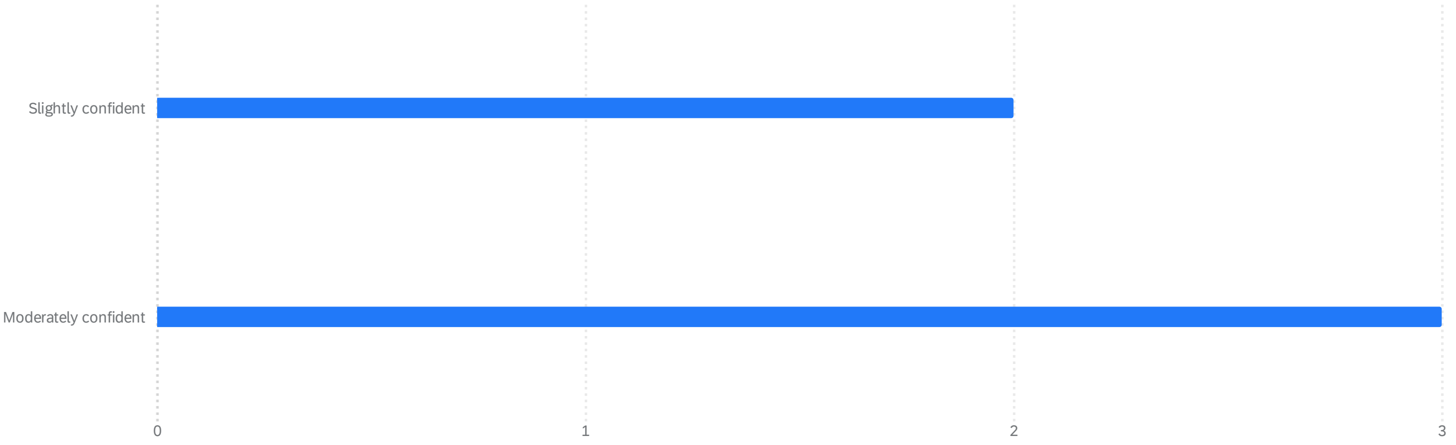

How confident are you in performing inpatient preoperative assessment? 5

Q8 - How confident are you in performing inpatient preoperative assessment?	Count	Count
Slightly confident	40%	2
Moderately confident	60%	3

How confident are you in performing inpatient preoperative assessment? 5

Q8 - How confident are you in performing inpatient preoperative assessment?	Average (Q8 - How confident are you in performing inpatient preoperative assessment?)	Minimum (Q8 - How confident are you in performing inpatient preoperative assessment?)	Maximum (Q8 - How confident are you in performing inpatient preoperative assessment?)	Count
Moderately confident	3.00	3.00	3.00	3
Slightly confident	2.00	2.00	2.00	2

How confident are you in applying the ACC/AHA guidelines for perioperative cardiovascular evaluation? 5

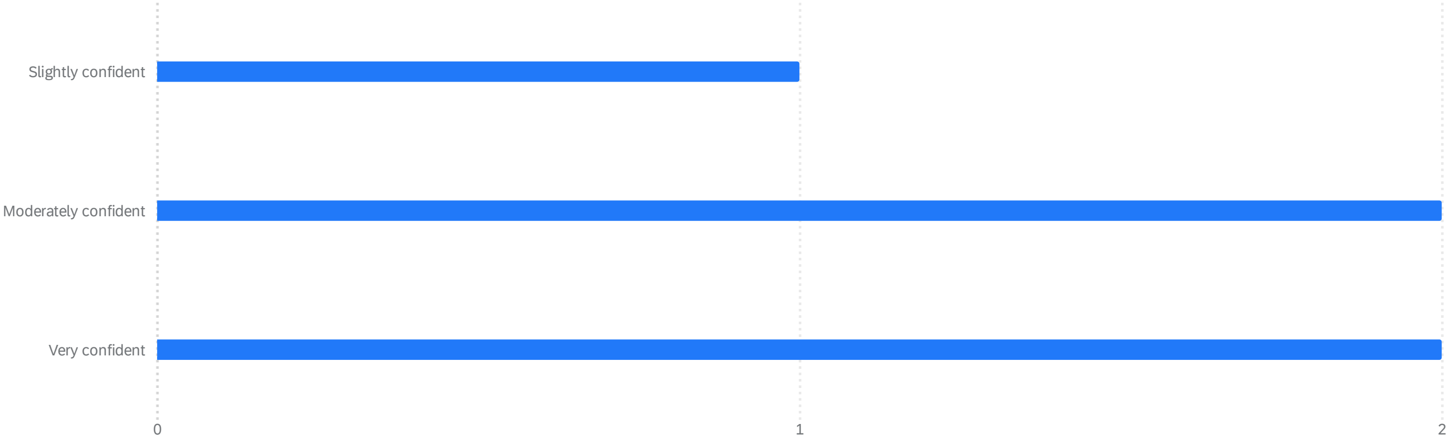

How confident are you in applying the ACC/AHA guidelines for perioperative cardiovascular evaluation? 5

Q9 - How confident are you in applying the ACC/AHA guidelines for perioperative cardiovascular evaluation?	Count	Count
Slightly confident	20%	1
Moderately confident	40%	2
Very confident	40%	2

How confident are you in applying the ACC/AHA guidelines for perioperative cardiovascular evaluation? 5

Q9 - How confident are you in applying the ACC/AHA guidelines for perioperative cardiovascular evaluation?	Average (Q9 - How confident are you in applying the ACC/AHA guidelines for perioperative cardiovascular evaluation?)	Minimum (Q9 - How confident are you in applying the ACC/AHA guidelines for perioperative cardiovascular evaluation?)	Maximum (Q9 - How confident are you in applying the ACC/AHA guidelines for perioperative cardiovascular evaluation?)	Count
Moderately confident	3.00	3.00	3.00	2
Slightly confident	2.00	2.00	2.00	1
Very confident	4.00	4.00	4.00	2

How confident are you in managing patients undergoing bariatric surgery preoperatively? 5

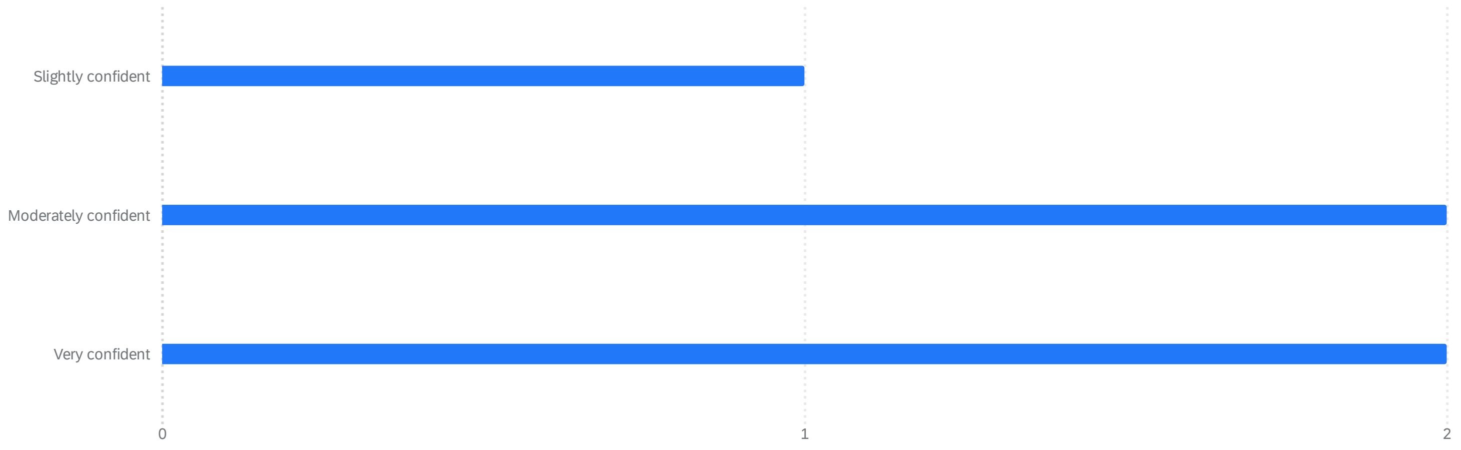

How confident are you in managing patients undergoing bariatric surgery preoperatively? 5

Q10 - How confident are you in managing patients undergoing bariatric surgery preoperatively?	Count	Count
Slightly confident	20%	1
Moderately confident	40%	2
Very confident	40%	2

How confident are you in managing patients undergoing bariatric surgery preoperatively? 5

Q10 - How confident are you in managing patients undergoing bariatric surgery preoperatively?	Average (Q10 - How confident are you in managing patients undergoing bariatric surgery preoperatively?)	Minimum (Q10 - How confident are you in managing patients undergoing bariatric surgery preoperatively?)	Maximum (Q10 - How confident are you in managing patients undergoing bariatric surgery preoperatively?)	Count
Moderately confident	3.00	3.00	3.00	2
Slightly confident	2.00	2.00	2.00	1
Very confident	4.00	4.00	4.00	2

How confident are you in managing patients with chronic kidney disease preoperatively? 5

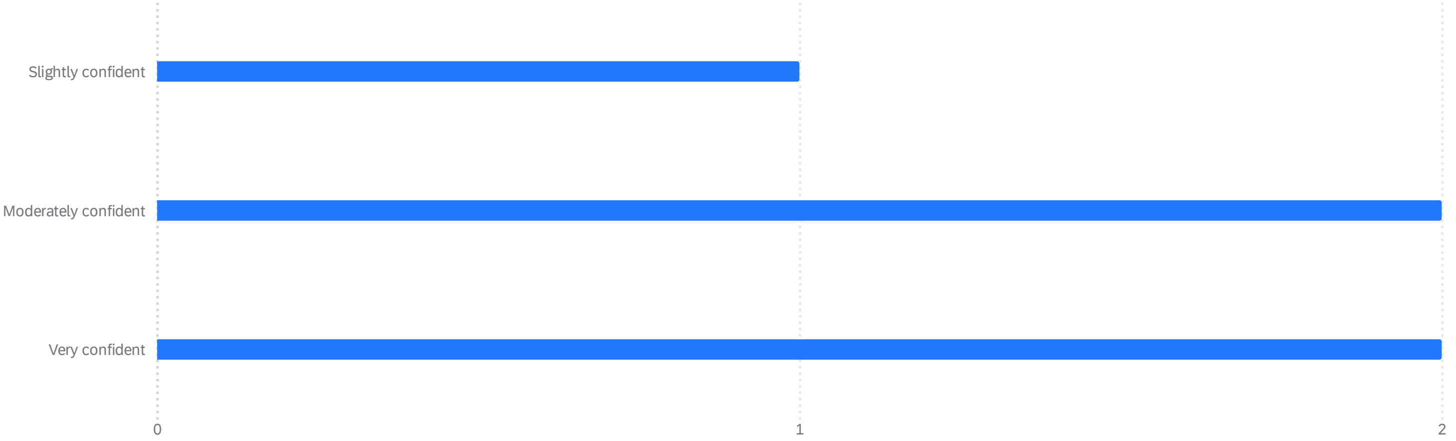

How confident are you in managing patients with chronic kidney disease preoperatively? 5

Q11 - How confident are you in managing patients with chronic kidney disease preoperatively?	Count	Count
Slightly confident	20%	1
Moderately confident	40%	2
Very confident	40%	2

How confident are you in managing patients with chronic kidney disease preoperatively? 5

Q11 - How confident are you in managing patients with chronic kidney disease preoperatively?	Average (Q11 - How confident are you in managing patients with chronic kidney disease preoperatively?)	Minimum (Q11 - How confident are you in managing patients with chronic kidney disease preoperatively?)	Maximum (Q11 - How confident are you in managing patients with chronic kidney disease preoperatively?)	Count
Moderately confident	3.00	3.00	3.00	2
Slightly confident	2.00	2.00	2.00	1
Very confident	4.00	4.00	4.00	2

How confident are you in managing patients with liver disease preoperatively? 5

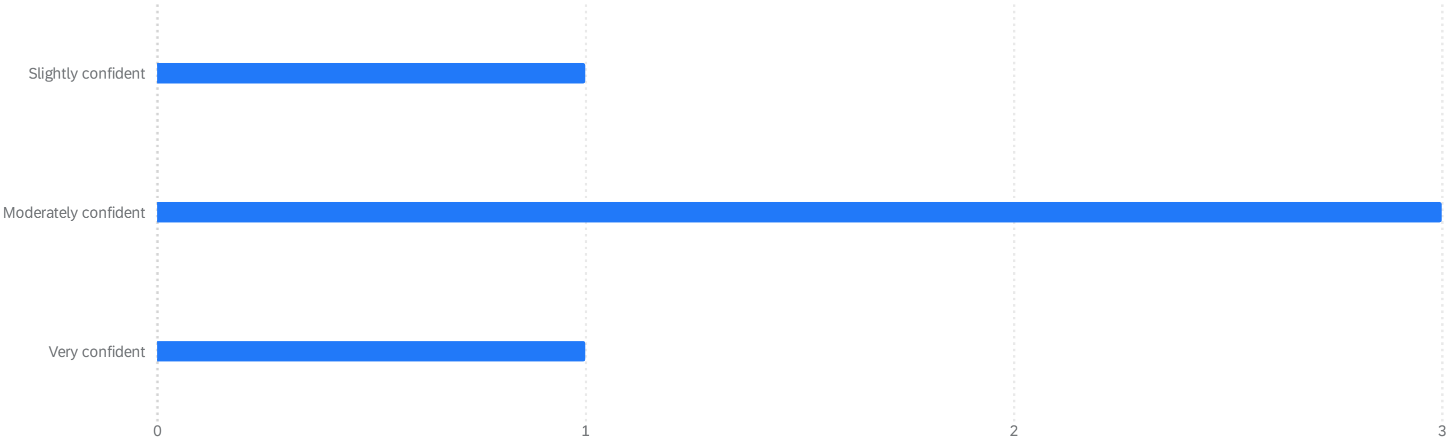

How confident are you in managing patients with liver disease preoperatively? 5

Q12 - How confident are you in managing patients with liver disease preoperatively?	Count	Count
Slightly confident	20%	1
Moderately confident	60%	3
Very confident	20%	1

How confident are you in managing patients with liver disease preoperatively? 5

Q12 - How confident are you in managing patients with liver disease preoperatively?	Average (Q12 - How confident are you in managing patients with liver disease preoperatively?)	Minimum (Q12 - How confident are you in managing patients with liver disease preoperatively?)	Maximum (Q12 - How confident are you in managing patients with liver disease preoperatively?)	Count
Moderately confident	3.00	3.00	3.00	3
Slightly confident	2.00	2.00	2.00	1
Very confident	4.00	4.00	4.00	1

How confident are you in managing patients with rheumatologic conditions (e.g., RA) preoperatively? 5

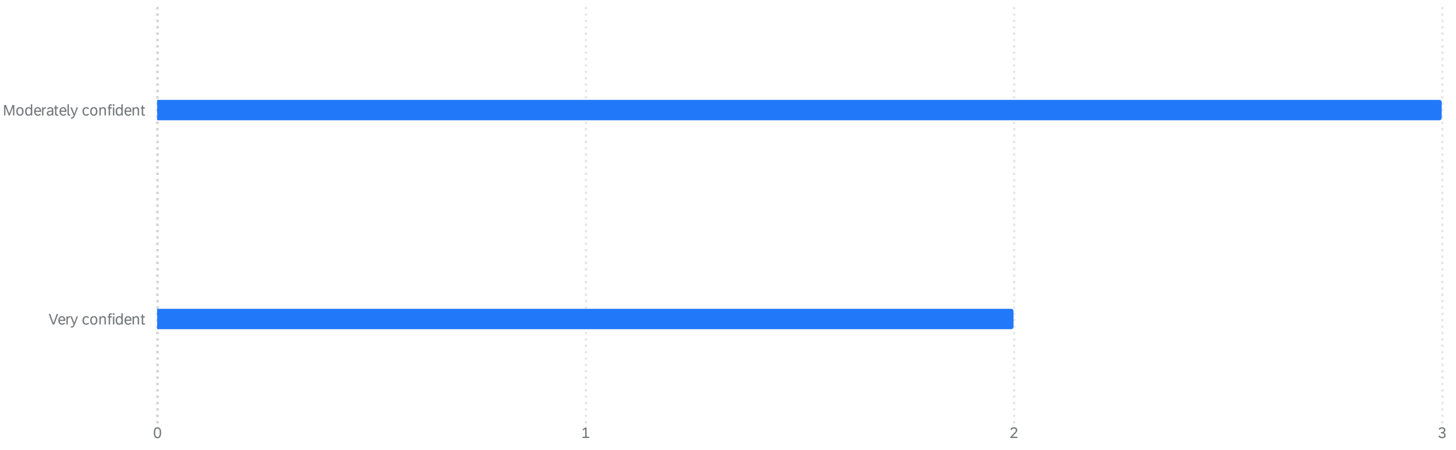

How confident are you in managing patients with rheumatologic conditions (e.g., RA) preoperatively? 5

Q13 - How confident are you in managing patients with rheumatologic conditions (e.g., RA) preoperatively?	Count	Count
Moderately confident	60%	3
Very confident	40%	2

How confident are you in managing patients with rheumatologic conditions (e.g., RA) preoperatively? 5

Q13 - How confident are you in managing patients with rheumatologic conditions (e.g., RA) preoperatively?	Average (Q13 - How confident are you in managing patients with rheumatologic conditions (e.g., RA) preoperatively?)	Minimum (Q13 - How confident are you in managing patients with rheumatologic conditions (e.g., RA) preoperatively?)	Maximum (Q13 - How confident are you in managing patients with rheumatologic conditions (e.g., RA) preoperatively?)	Count
Moderately confident	3.00	3.00	3.00	3
Very confident	4.00	4.00	4.00	2

Guidelines recommend utilizing risk calculators to estimate risk of major adverse cardiac events (MACE). What is the percentage cut off? 4

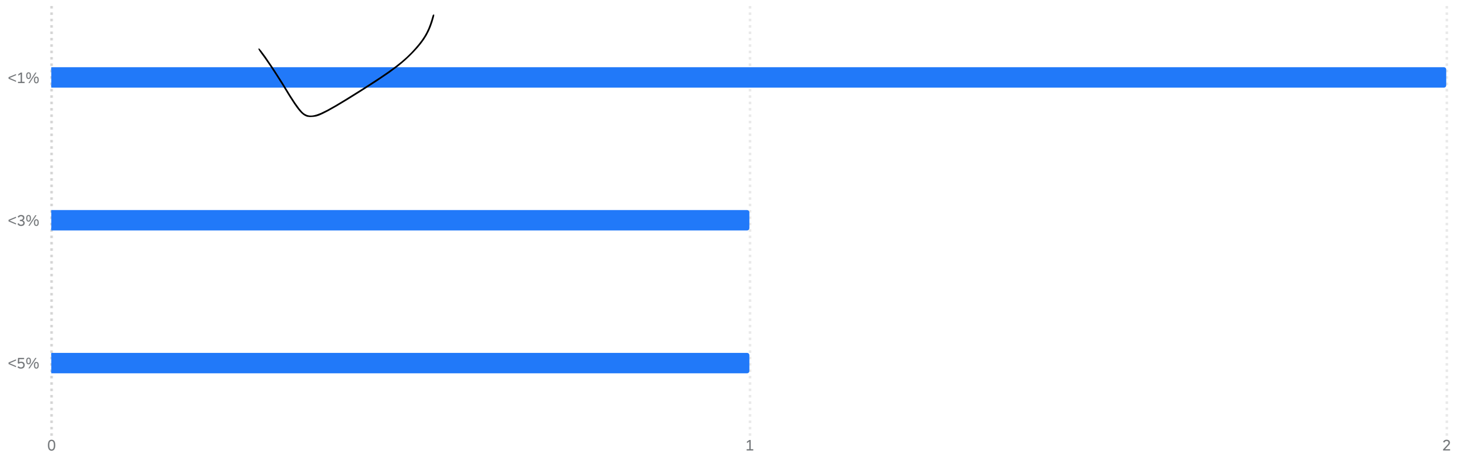

Guidelines recommend utilizing risk calculators to estimate risk of major adverse cardiac events (MACE). What is the percentage cut off? 4

Q14 - Guidelines recommend utilizing risk calculators to estimate risk of major adverse cardiac events (MACE). What is the percentage cut off?	Count	Count
<1%	50%	2
<3%	25%	1
<5%	25%	1

Guidelines recommend utilizing risk calculators to estimate risk of major adverse cardiac events (MACE). What is the percentage cut off? 4

Q14 - Guidelines recommend utilizing risk calculators to estimate risk of major adverse cardiac events (MACE). What is the percentage cut off?	Average (Q14 - Guidelines recommend utilizing risk calculators to estimate risk of major adverse cardiac events (MACE). What is the percentage cut off?)	Minimum (Q14 - Guidelines recommend utilizing risk calculators to estimate risk of major adverse cardiac events (MACE). What is the percentage cut off?)	Maximum (Q14 - Guidelines recommend utilizing risk calculators to estimate risk of major adverse cardiac events (MACE). What is the percentage cut off?)	Count
<1%	1.00	1.00	1.00	2
<3%	2.00	2.00	2.00	1
<5%	3.00	3.00	3.00	1

For patients who received bare metal stents, which of the following is a TRUE statement? 4

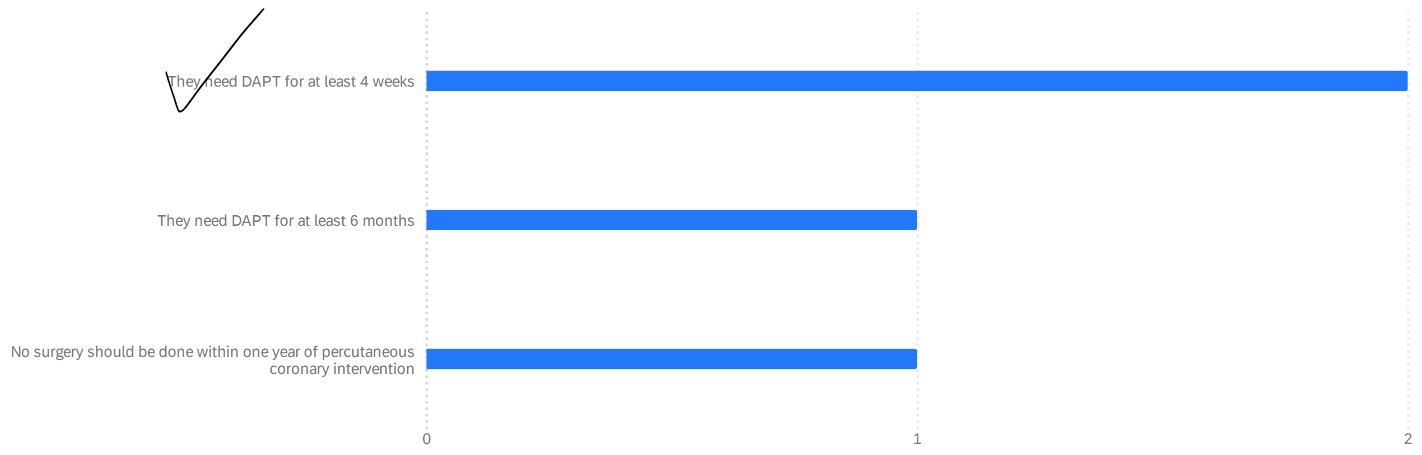

For patients who received bare metal stents, which of the following is a TRUE statement? 4

Q15 - For patients who received bare metal stents, which of the following is a TRUE statement?	Count	Count
They need DAPT for at least 4 weeks	50%	2
They need DAPT for at least 6 months	25%	1
No surgery should be done within one year of percutaneous coronary intervention	25%	1

For patients who received bare metal stents, which of the following is a TRUE statement? 4

Q15 - For patients who received bare metal stents, which of the following is a TRUE statement?	Average (Q15 - For patients who received bare metal stents, which of the following is a TRUE statement?)	Minimum (Q15 - For patients who received bare metal stents, which of the following is a TRUE statement?)	Maximum (Q15 - For patients who received bare metal stents, which of the following is a TRUE statement?)	Count
No surgery should be done within one year of percutaneous coronary intervention	3.00	3.00	3.00	1
They need DAPT for at least 4 weeks	1.00	1.00	1.00	2
They need DAPT for at least 6 months	2.00	2.00	2.00	1

For patients who received drug-eluting stents, what is the recommended duration for dual anti-platelet therapy? 4

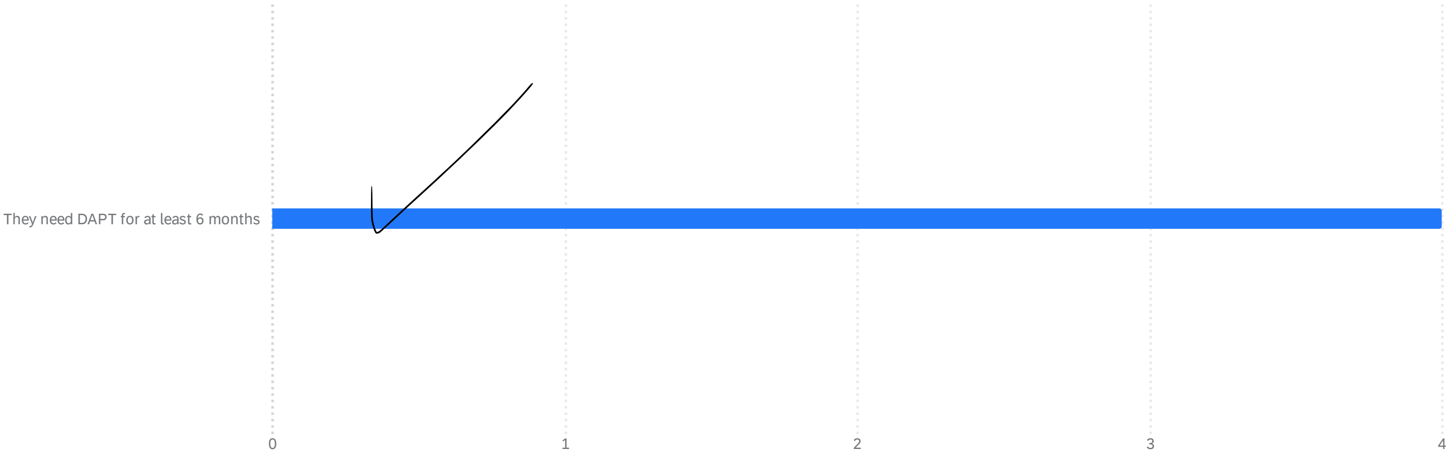

For patients who received drug-eluting stents, what is the recommended duration for dual anti-platelet therapy? 4

Q16 - For patients who received drug-eluting stents, what is the recommended duration for dual anti-platelet therapy?	Count	Count
They need DAPT for at least 6 months	100%	4

For patients who received drug-eluting stents, what is the recommended duration for dual anti-platelet therapy? 4

Q16 - For patients who received drug-eluting stents, what is the recommended duration for dual anti-platelet therapy?	Average (Q16 - For patients who received drug-eluting stents, what is the recommended duration for dual anti-platelet therapy?)	Minimum (Q16 - For patients who received drug-eluting stents, what is the recommended duration for dual anti-platelet therapy?)	Maximum (Q16 - For patients who received drug-eluting stents, what is the recommended duration for dual anti-platelet therapy?)	Count
They need DAPT for at least 6 months	2.00	2.00	2.00	4

Which statement is true? 4

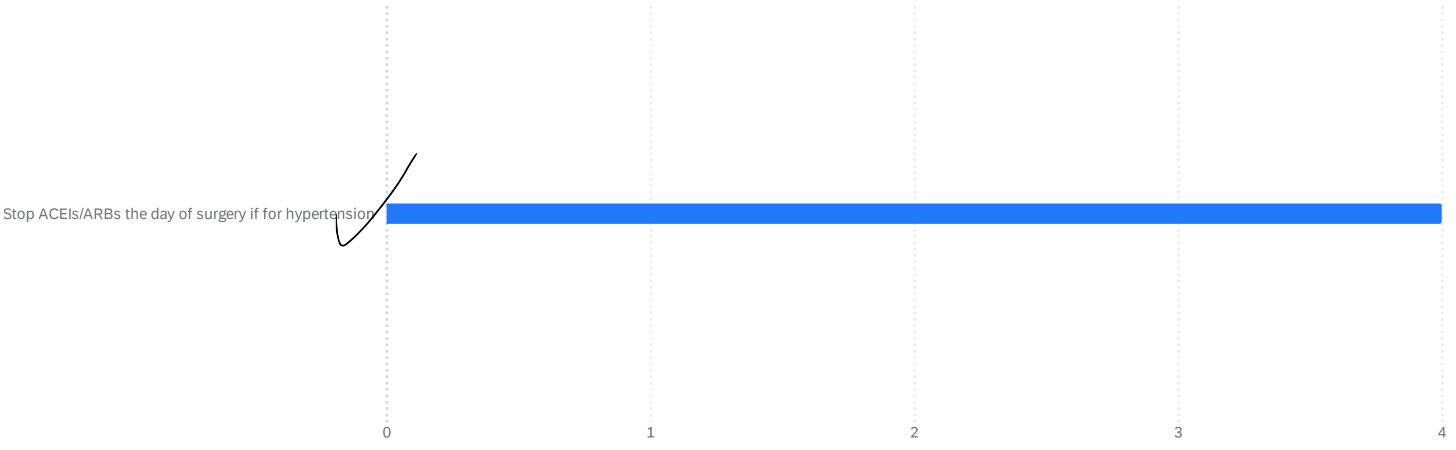

Which statement is true? 4

Q17 - Which statement is true?	Count	Count
Stop ACEIs/ARBs the day of surgery if for hypertension	100%	4

Which statement is true? 4

Q17 - Which statement is true?	Average (Q17 - Which statement is true?)	Minimum (Q17 - Which statement is true?)	Maximum (Q17 - Which statement is true?)	Count
Stop ACEIs/ARBs the day of surgery if for hypertension	3.00	3.00	3.00	4

Which set of patients require stress dose steroids to prevent adrenal crisis during surgery? 4

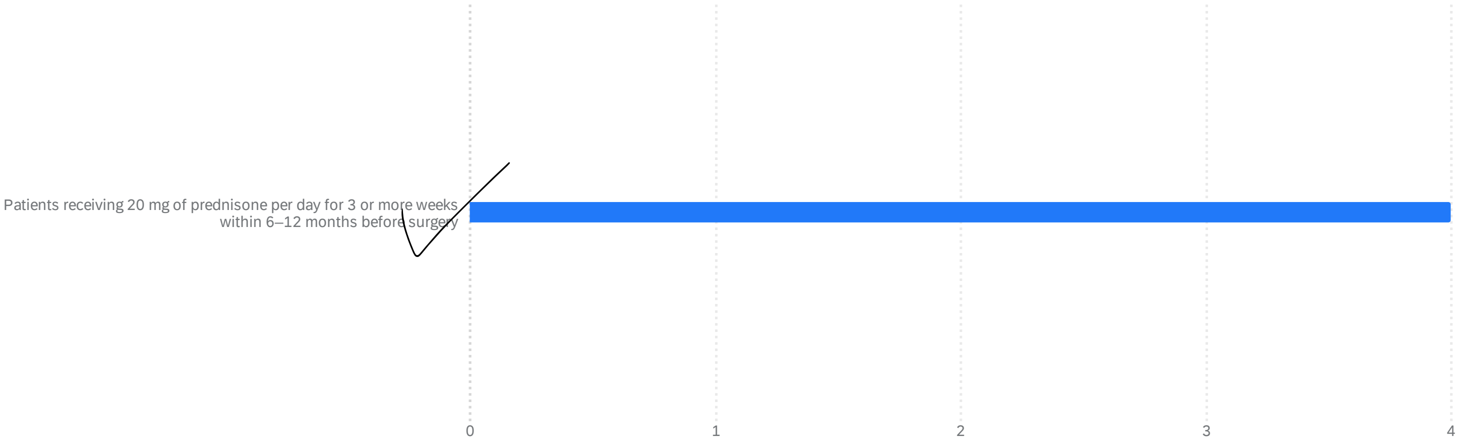

Which set of patients require stress dose steroids to prevent adrenal crisis during surgery? 4

Q18 - Which set of patients require stress dose steroids to prevent adrenal crisis during surgery?	Count	Count
Patients receiving 20 mg of prednisone per day for 3 or more weeks within 6–12 months before surgery	100%	4

Which set of patients require stress dose steroids to prevent adrenal crisis during surgery? 4

Q18 - Which set of patients require stress dose steroids to prevent adrenal crisis during surgery?	Average (Q18 - Which set of patients require stress dose steroids to prevent adrenal crisis during surgery?)	Minimum (Q18 - Which set of patients require stress dose steroids to prevent adrenal crisis during surgery?)	Maximum (Q18 - Which set of patients require stress dose steroids to prevent adrenal crisis during surgery?)	Count
Patients receiving 20 mg of prednisone per day for 3 or more weeks within 6–12 months before surgery	2.00	2.00	2.00	4

Which of the following statements are true regarding insulin management prior to surgery for patients with type I diabetes? (Select all that apply) 4

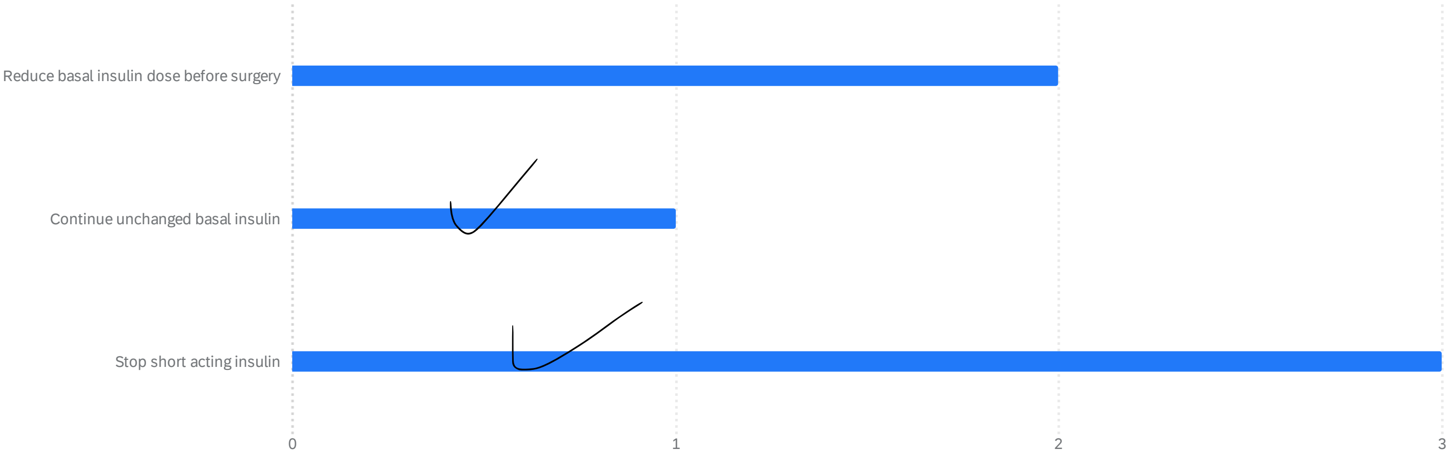

Which of the following statements are true regarding insulin management prior to surgery for patients with type I diabetes? (Select all that apply) 4

Q19 - Which of the following statements are true regarding insulin management prior to surgery for patients with type I diabetes? (Select all that apply)	Count	Count
Reduce basal insulin dose before surgery	50%	2
Continue unchanged basal insulin	25%	1
Stop short acting insulin	75%	3

Which statement regarding SGLT2 inhibitors is true? (Select all that apply) 4

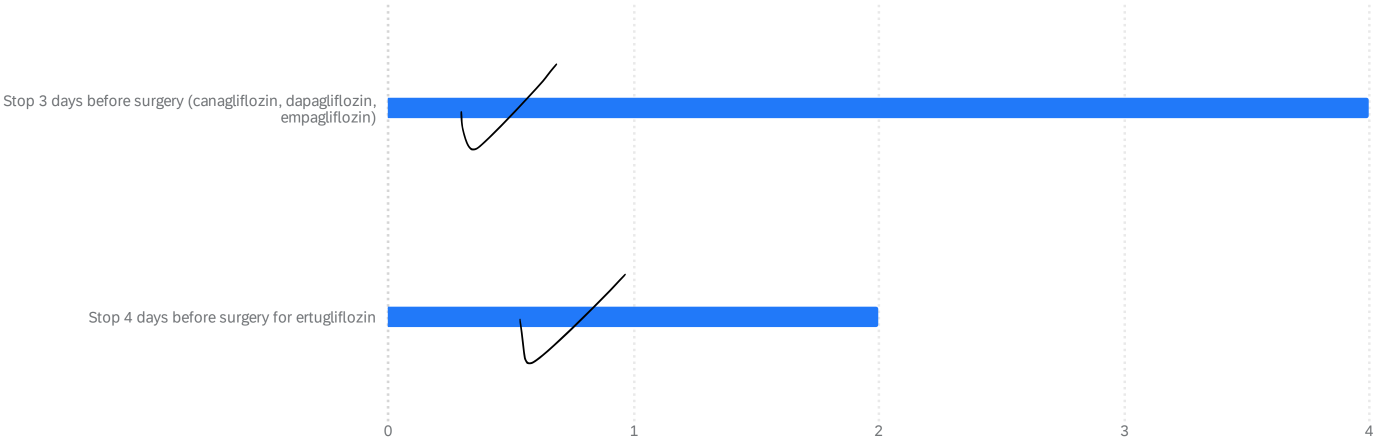

Which statement regarding SGLT2 inhibitors is true? (Select all that apply) 4

Q20 - Which statement regarding SGLT2 inhibitors is true? (Select all that apply)	Count	Count
Stop 3 days before surgery (canagliflozin, dapagliflozin, empagliflozin)	100%	4
Stop 4 days before surgery for ertugliflozin	50%	2

Which rheumatologic agents are safe to continue before surgery? (Select all that apply) 4

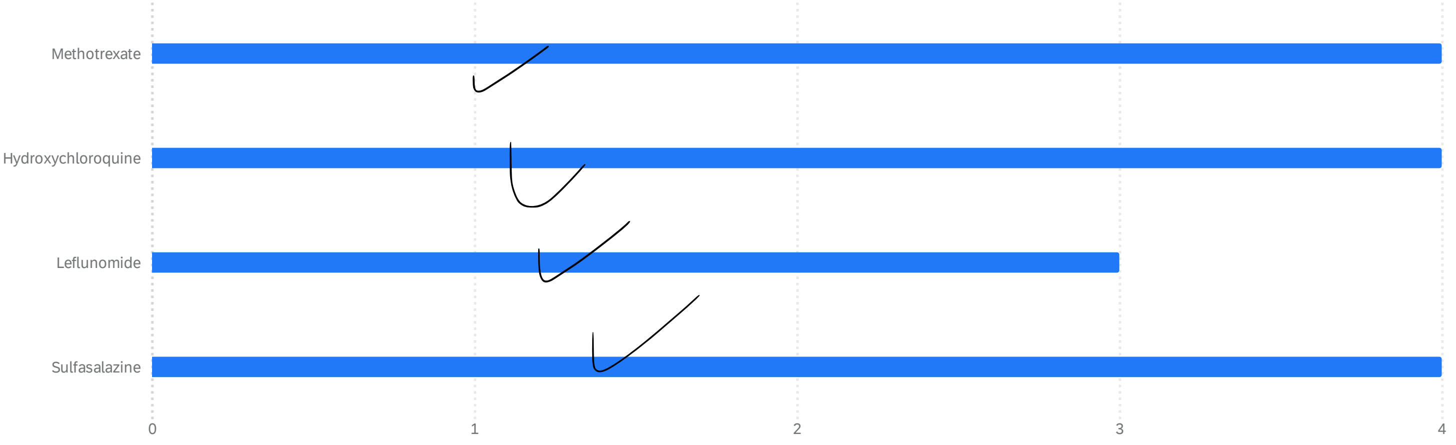

Which rheumatologic agents are safe to continue before surgery? (Select all that apply) 4

Q21 - Which rheumatologic agents are safe to continue before surgery? (Select all that apply)	Count	Count
Methotrexate	100%	4
Hydroxychloroquine	100%	4
Leflunomide	75%	3
Sulfasalazine	100%	4

Which statement regarding bridging is true? (Select all that apply) 4

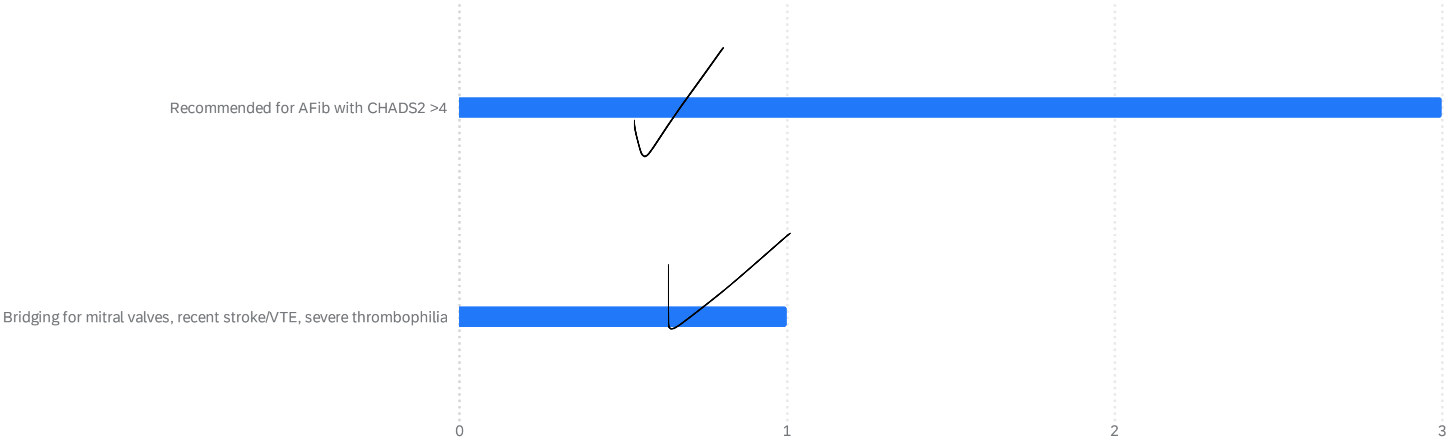

Which statement regarding bridging is true? (Select all that apply) 4

Q22 - Which statement regarding bridging is true? (Select all that apply)	Count	Count
Recommended for AFib with CHADS2 >4	75%	3
Bridging for mitral valves, recent stroke/VTE, severe thrombophilia	25%	1

For patients on warfarin, you would check INR 7–10 days before the procedure. Which of the following is TRUE? (Select all that apply) 4

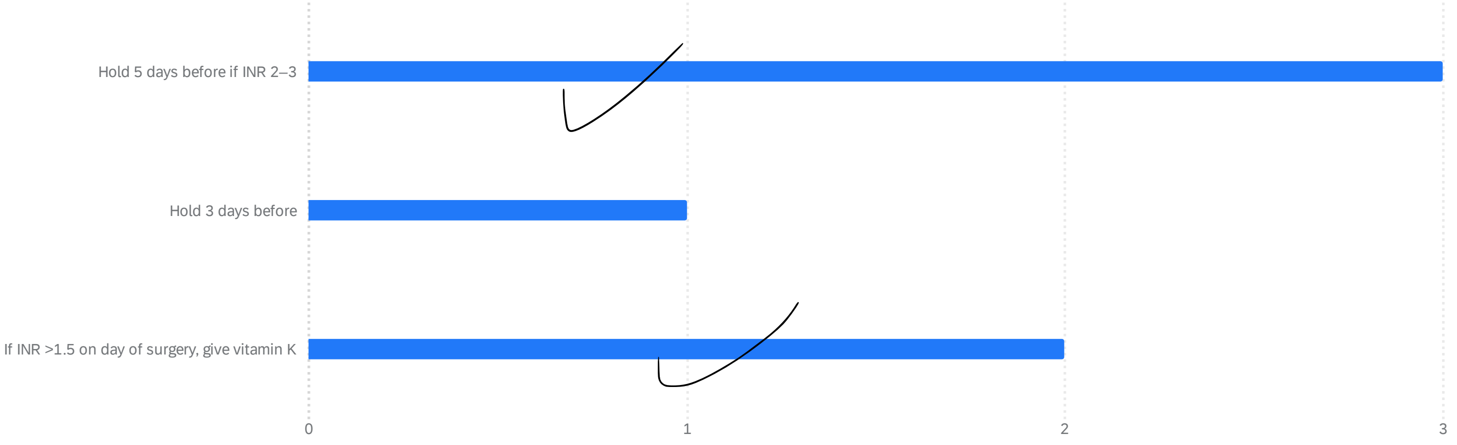

For patients on warfarin, you would check INR 7–10 days before the procedure. Which of the following is TRUE? (Select all that apply) 4

Q23 - For patients on warfarin, you would check INR 7–10 days before the procedure. Which of the following is TRUE? (Select all that apply)	Count	Count
Hold 5 days before if INR 2–3	75%	3
Hold 3 days before	25%	1
If INR >1.5 on day of surgery, give vitamin K	50%	2

How do you restart warfarin after low-moderate bleeding risk procedure? 4

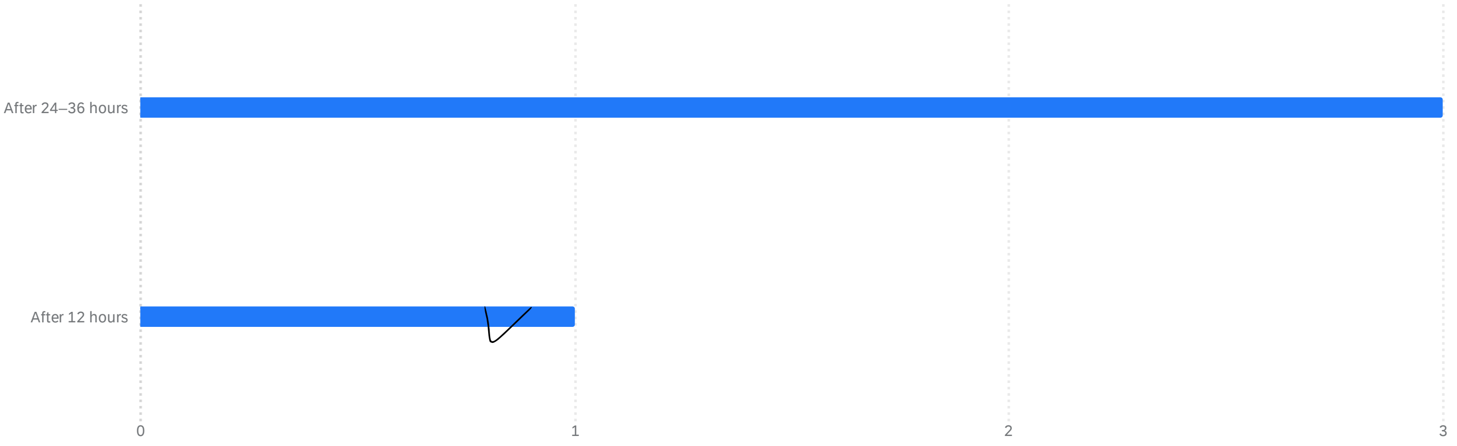

How do you restart warfarin after low-moderate bleeding risk procedure? 4

Q24 - How do you restart warfarin after low-moderate bleeding risk procedure?	Count	Count
After 24–36 hours	75%	3
After 12 hours	25%	1

How do you restart warfarin after low-moderate bleeding risk procedure? 4

Q24 - How do you restart warfarin after low-moderate bleeding risk procedure?	Average (Q24 - How do you restart warfarin after low-moderate bleeding risk procedure?)	Minimum (Q24 - How do you restart warfarin after low-moderate bleeding risk procedure?)	Maximum (Q24 - How do you restart warfarin after low-moderate bleeding risk procedure?)	Count
After 12 hours	2.00	2.00	2.00	1
After 24–36 hours	1.00	1.00	1.00	3

Which is true regarding DOACs and normal CrCl? 4

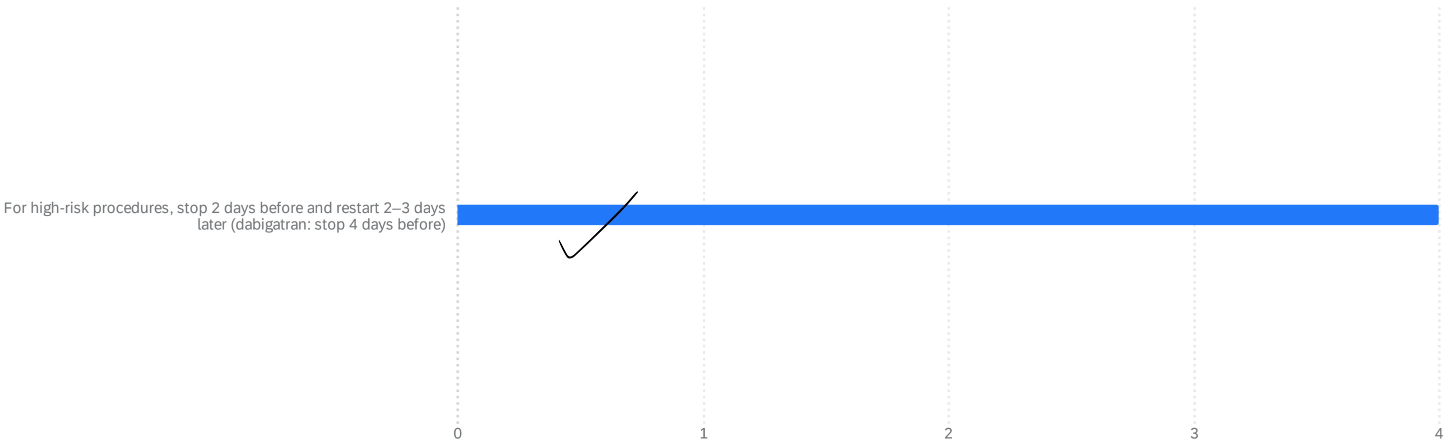

Which is true regarding DOACs and normal CrCl? 4

Q25 - Which is true regarding DOACs and normal CrCl?	Count	Count
For high-risk procedures, stop 2 days before and restart 2–3 days later (dabigatran: stop 4 days before)	100%	4

Which is true regarding DOACs and normal CrCl? 4

Q25 - Which is true regarding DOACs and normal CrCl?	Average (Q25 - Which is true regarding DOACs and normal CrCl?)	Minimum (Q25 - Which is true regarding DOACs and normal CrCl?)	Maximum (Q25 - Which is true regarding DOACs and normal CrCl?)	Count
For high-risk procedures, stop 2 days before and restart 2–3 days later (dabigatran: stop 4 days before)	1.00	1.00	1.00	4

What calculator(s) can be used to assess VTE risk in bariatric surgery patients? (Select all that apply) 4

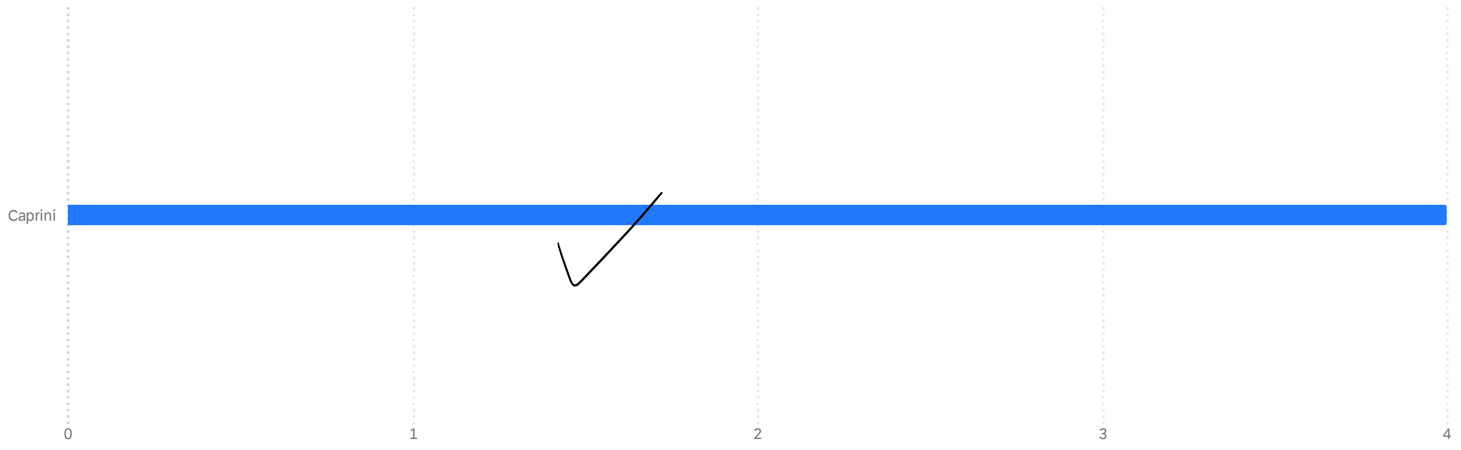

What calculator(s) can be used to assess VTE risk in bariatric surgery patients? (Select all that apply) 4

Q26 - What calculator(s) can be used to assess VTE risk in bariatric surgery patients? (Select all that apply)	Count	Count
Caprini	100%	4

Please provide feedback for improvement: 7

Please provide feedback for improvement:
