# Supplementary material for: Escaping the OR: a pilot study of a Jigsaw-based workshop to teach preoperative assessment in internal medicine residency
Source: BMC Med Educ. 2026 May 16;26:1097. doi: 10.1186/s12909-026-09419-w (PMC13348809; doi:10.1186/s12909-026-09419-w)
Supplement: Supplementary file 1 — Supplementary Material 1. [file 12909_2026_9419_MOESM1_ESM.zip › Post_Survey_Final.docx]

# Pre- and Post-Intervention Survey

Internal Medicine Residency Program

## Demographics

Age: ______

Gender:
☐ Male
☐ Female
☐ Other
☐ Prefer not to say

Ethnicity (Check all that apply):

☐ Asian/Pacific Islander
☐ Caucasian
☐ Black/African American
☐ Hispanic/Latino
☐ Native American
☐ Other: _______
☐ Prefer not to answer

Please indicate your PGY year:
☐ PGY1
☐ PGY2
☐ PGY3

## Prior Experience

Have you received training in ambulatory preoperative assessment in the following settings?
☐ Undergraduate coursework
☐ Medical school
☐ Graduate school
☐ Residency
☐ Self-study
☐ None
☐ Other: ____________

Have you received training in inpatient preoperative assessment in the following settings?
☐ Undergraduate coursework
☐ Medical school
☐ Graduate school
☐ Residency
☐ Self-study
☐ None
☐ Other: ____________

## Confidence Ratings (Use the same scale for all):

### How confident are you in performing ambulatory preoperative assessment?

☐ Not at all confident

☐ Slightly confident

☐ Moderately confident

☐ Very confident

☐ Extremely confident

### How confident are you in performing inpatient preoperative assessment?

☐ Not at all confident

☐ Slightly confident

☐ Moderately confident

☐ Very confident

☐ Extremely confident

### How confident are you in applying the ACC/AHA guidelines for perioperative cardiovascular evaluation?

☐ Not at all confident

☐ Slightly confident

☐ Moderately confident

☐ Very confident

☐ Extremely confident

### How confident are you in managing patients undergoing bariatric surgery preoperatively?

☐ Not at all confident

☐ Slightly confident

☐ Moderately confident

☐ Very confident

☐ Extremely confident

### How confident are you in managing patients with chronic kidney disease preoperatively?

☐ Not at all confident

☐ Slightly confident

☐ Moderately confident

☐ Very confident

☐ Extremely confident

### How confident are you in managing patients with liver disease preoperatively?

☐ Not at all confident

☐ Slightly confident

☐ Moderately confident

☐ Very confident

☐ Extremely confident

### How confident are you in managing patients with obstructive sleep apnea (OSA) preoperatively?

☐ Not at all confident

☐ Slightly confident

☐ Moderately confident

☐ Very confident

☐ Extremely confident

### How confident are you in managing patients with rheumatologic conditions (e.g., RA) preoperatively?

☐ Not at all confident

☐ Slightly confident

☐ Moderately confident

☐ Very confident

☐ Extremely confident

## Knowledge Questions

Guidelines recommend utilizing risk calculators to estimate risk of major adverse cardiac events (MACE). What is the percentage cut off?
x <1%
☐ <3%
☐ <5%
☐ <10%

For patients who received bare metal stents, which of the following is a TRUE statement?
x They need DAPT for at least 4 weeks
☐ They need DAPT for at least 6 months
☐ No surgery should be done within one year of percutaneous coronary intervention

For patients who received drug-eluting stents, what is the recommended duration for dual anti-platelet therapy?
☐ They need DAPT for at least 4 weeks
x They need DAPT for at least 6 months
☐ No surgery should be done within one year of percutaneous coronary intervention

Which statement is true?
☐ Stop aspirin 48 hours before surgery
☐ Stop anti-epileptics 72 hours before surgery
x Stop ACEIs/ARBs the day of surgery if for hypertension
☐ Continue all medications before surgery

Which set of patients require stress dose steroids to prevent adrenal crisis during surgery?
☐ Patients who had chronic COPD/asthma with bursts of prednisone one year ago
x Patients receiving 20 mg of prednisone per day for 3 or more weeks within 6–12 months before surgery
☐ Patients with diabetes
☐ Patients with hyperthyroidism

Which of the following statements are true regarding insulin management prior to surgery for patients with type I diabetes? (Select all that apply)
☐ Reduce basal insulin dose before surgery
x Continue unchanged basal insulin
☐ Stop basal insulin completely
☐ Continue short acting insulin
x Stop short acting insulin

Which statement regarding SGLT2 inhibitors is true? (Select all that apply)
x Stop 3 days before surgery (canagliflozin, dapagliflozin, empagliflozin)
x Stop 4 days before surgery for ertugliflozin
☐ Do not stop these medications

Which rheumatologic agents are safe to continue before surgery? (Select all that apply)
x Methotrexate
x Hydroxychloroquine
x Leflunomide
x Sulfasalazine
☐ Humira

Which statement regarding bridging is true? (Select all that apply)
☐ Recommended for AFib with CHADS2 <4
x Recommended for AFib with CHADS2 >4
☐ Bridging regardless of CHADS2
☐ Bridging not recommended due to bleeding risk
x Bridging for mitral valves, recent stroke/VTE, severe thrombophilia

For patients on warfarin, you would check INR 7–10 days before the procedure. Which of the following is TRUE? (Select all that apply)
x Hold 5 days before if INR 2–3
☐ Hold 3 days before
☐ Hold 1 day before
☐ Do not hold warfarin
x If INR >1.5 on day of surgery, give vitamin K

How do you restart warfarin after low-moderate bleeding risk procedure?
☐ After 24–36 hours
x After 12 hours
☐ Do not restart

Which is true regarding DOACs and normal CrCl?
x For high-risk procedures, stop 2 days before and restart 2–3 days later (dabigatran: stop 4 days before)
☐ For high-risk procedures, stop 1 day before (dabigatran: stop 3 days before)
☐ DOACs do not require interruption

What calculator(s) can be used to assess VTE risk in bariatric surgery patients? (Select all that apply)
x Rogers
x Caprini
☐ Well’s
☐ PERC
☐ RCRI
☐ GUPTA

## Open Feedback

Any feedback about the curriculum or preoperative medicine project?

_________________________________________________________

_________________________________________________________

_________________________________________________________
