# Supplementary material for: Escaping the OR: a pilot study of a Jigsaw-based workshop to teach preoperative assessment in internal medicine residency
Source: BMC Med Educ. 2026 May 16;26:1097. doi: 10.1186/s12909-026-09419-w (PMC13348809; doi:10.1186/s12909-026-09419-w)
Supplement: Supplementary file 1 — Supplementary Material 1. [file 12909_2026_9419_MOESM1_ESM.zip › Pre control.pdf]

PRE CONTROL Group Pre Op Survey / Page 1

Responses: 14

Age: 14

Age:

30

29

28

28

29

34

33

28

30

31

27

34

32

Gender 13

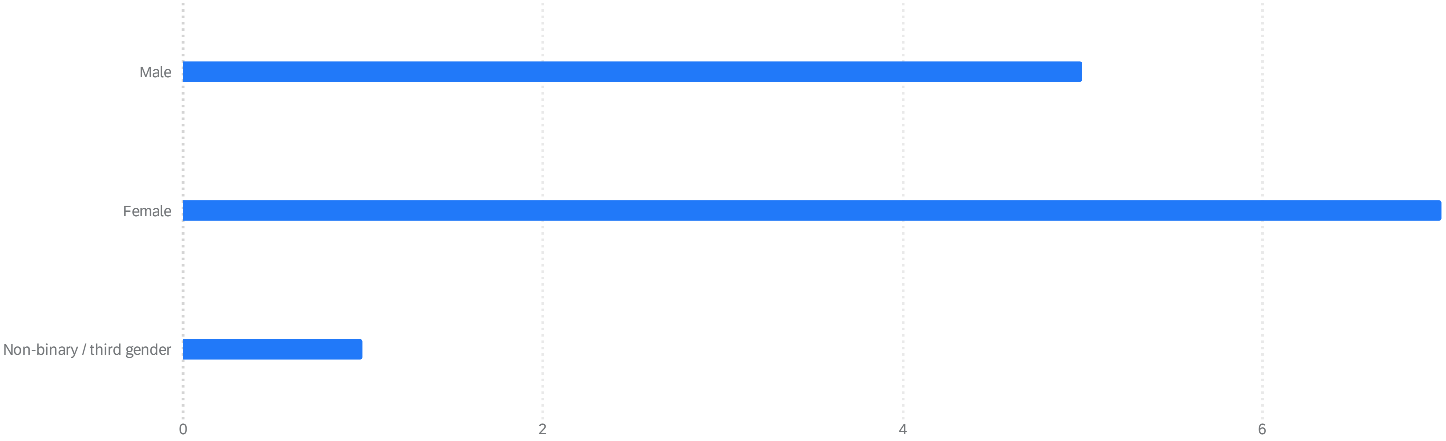

Gender 13

Q2 - Gender	Count	Count
Male	38%	5
Female	54%	7
Non-binary / third gender	8%	1

Gender 13

Q2 - Gender	Average (Q2 - Gender)	Minimum (Q2 - Gender)	Maximum (Q2 - Gender)	Count
Female	2.00	2.00	2.00	7
Male	1.00	1.00	1.00	5
Non-binary / third gender	3.00	3.00	3.00	1

Ethnicity (Check all that apply): 13

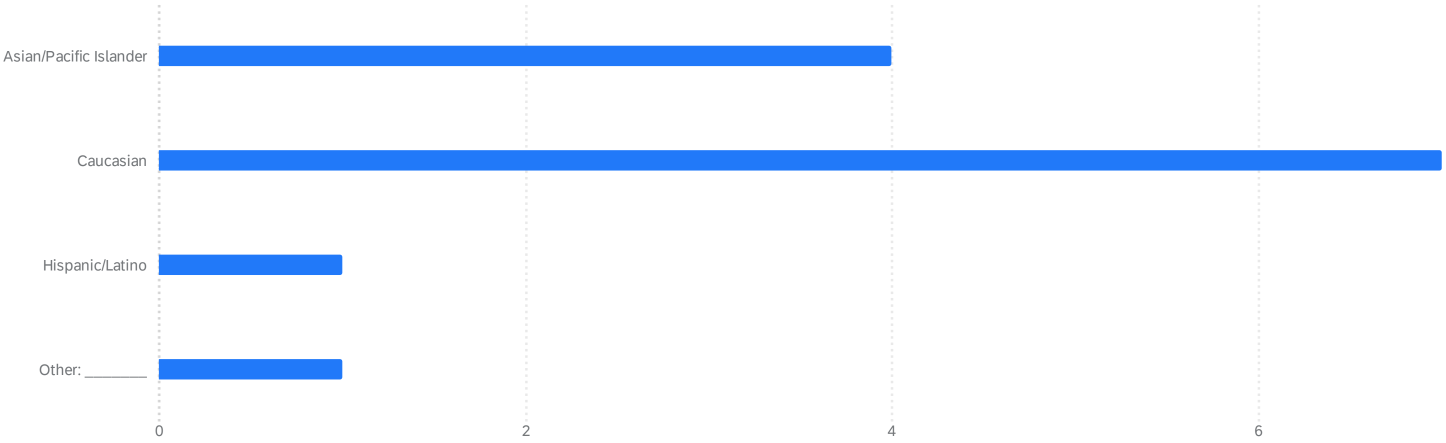

Ethnicity (Check all that apply): 13

Q3 - Ethnicity (Check all that apply):	Count	Count
Asian/Pacific Islander	31%	4
Caucasian	54%	7
Hispanic/Latino	8%	1
Other: ________	8%	1

Please indicate your PGY year: 13

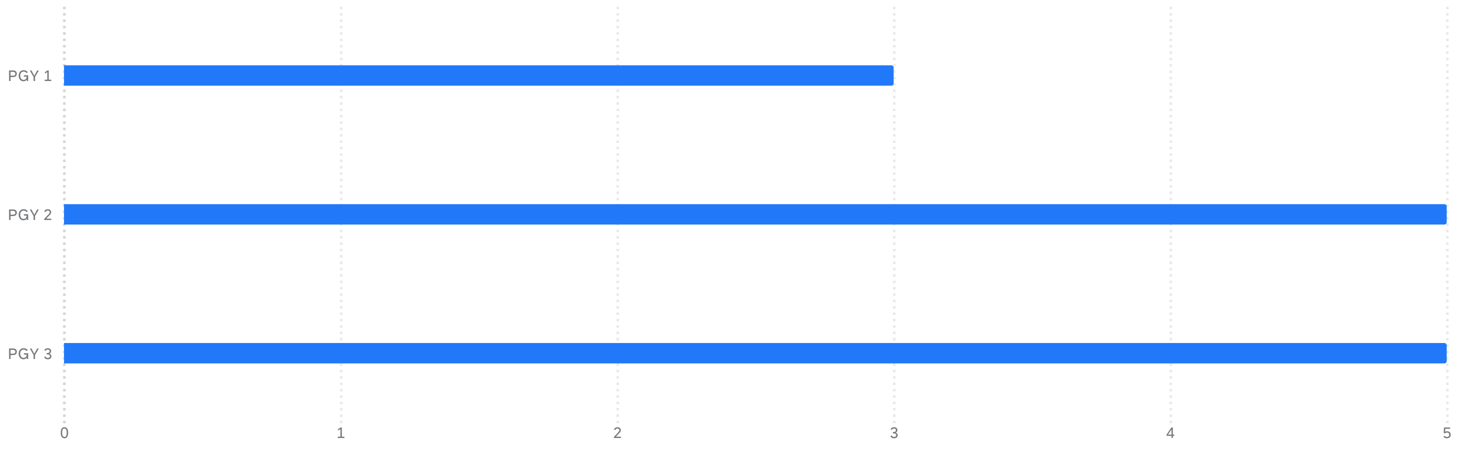

Please indicate your PGY year: 13

Q4 - Please indicate your PGY year:	Count	Count
PGY 1	23%	3
PGY 2	38%	5
PGY 3	38%	5

Please indicate your PGY year: 13

Q4 - Please indicate your PGY year:	Average (Q4 - Please indicate your PGY year:)	Minimum (Q4 - Please indicate your PGY year:)	Maximum (Q4 - Please indicate your PGY year:)	Count
PGY 1	1.00	1.00	1.00	3
PGY 2	2.00	2.00	2.00	5
PGY 3	3.00	3.00	3.00	5

Have you received training in ambulatory preoperative assessment in the following settings? 13

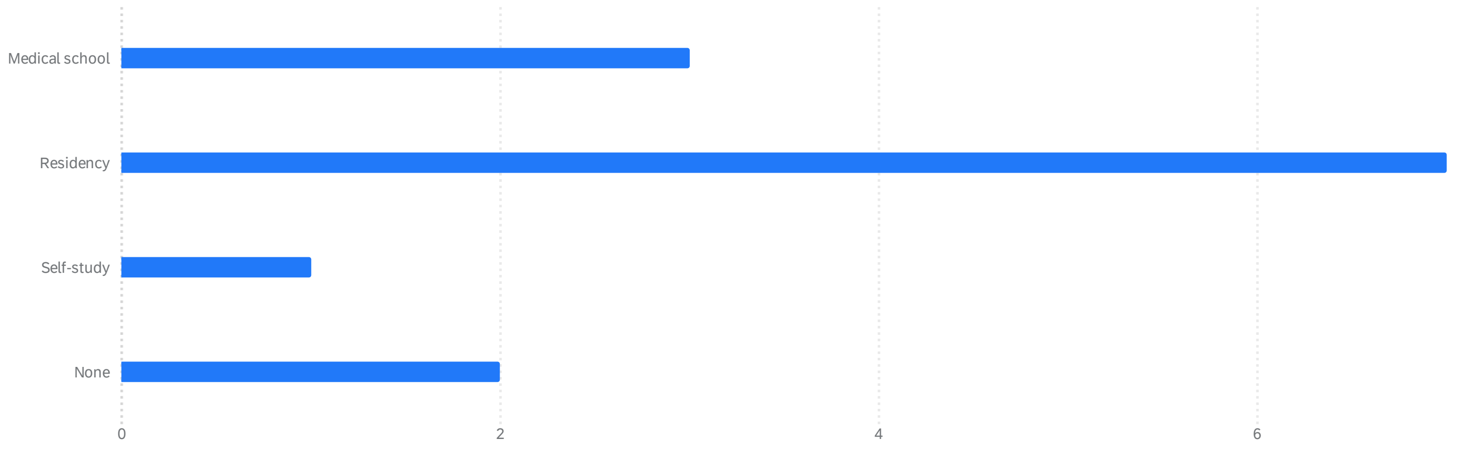

Have you received training in ambulatory preoperative assessment in the following settings? 13

Q5 - Have you received training in ambulatory preoperative assessment in the following settings?	Count	Count
Medical school	23%	3
Residency	54%	7
Self-study	8%	1
None	15%	2

Have you received training in ambulatory preoperative assessment in the following settings? 13

Q5 - Have you received training in ambulatory preoperative assessment in the following settings?	Average (Q5 - Have you received training in ambulatory preoperative assessment in the following settings?)	Minimum (Q5 - Have you received training in ambulatory preoperative assessment in the following settings?)	Maximum (Q5 - Have you received training in ambulatory preoperative assessment in the following settings?)	Count
Medical school	2.00	2.00	2.00	3
None	6.00	6.00	6.00	2
Residency	4.00	4.00	4.00	7
Self-study	5.00	5.00	5.00	1

Have you received training in inpatient preoperative assessment in the following settings? 13

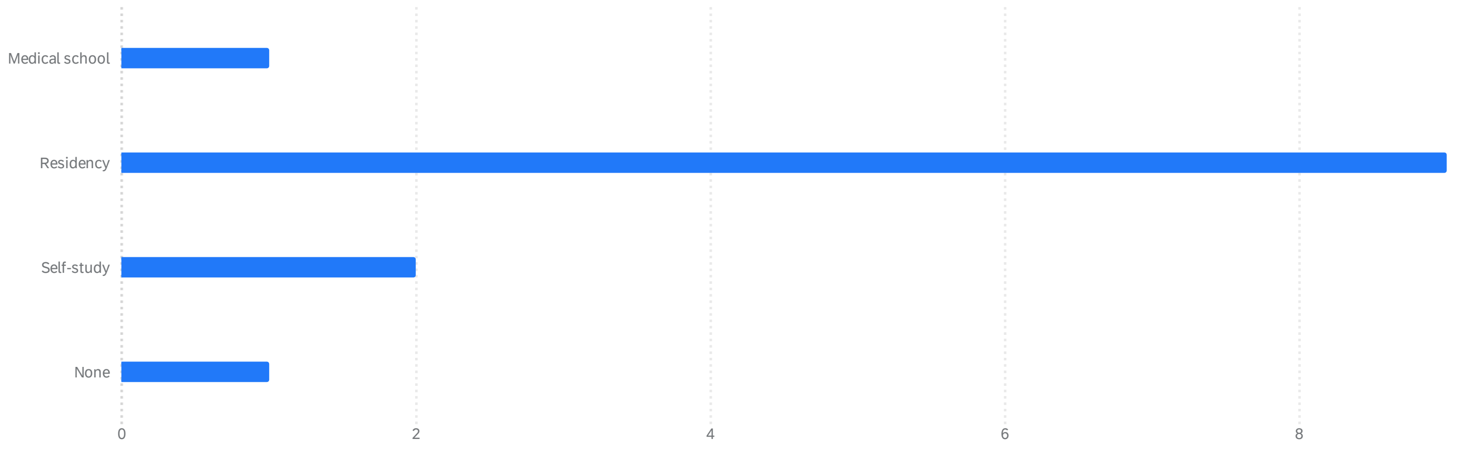

Have you received training in inpatient preoperative assessment in the following settings? 13

Q6 - Have you received training in inpatient preoperative assessment in the following settings?	Count	Count
Medical school	8%	1
Residency	69%	9
Self-study	15%	2
None	8%	1

Have you received training in inpatient preoperative assessment in the following settings? 13

Q6 - Have you received training in inpatient preoperative assessment in the following settings?	Average (Q6 - Have you received training in inpatient preoperative assessment in the following settings?)	Minimum (Q6 - Have you received training in inpatient preoperative assessment in the following settings?)	Maximum (Q6 - Have you received training in inpatient preoperative assessment in the following settings?)	Count
Medical school	2.00	2.00	2.00	1
None	6.00	6.00	6.00	1
Residency	4.00	4.00	4.00	9
Self-study	5.00	5.00	5.00	2

How confident are you in performing ambulatory preoperative assessment? 13

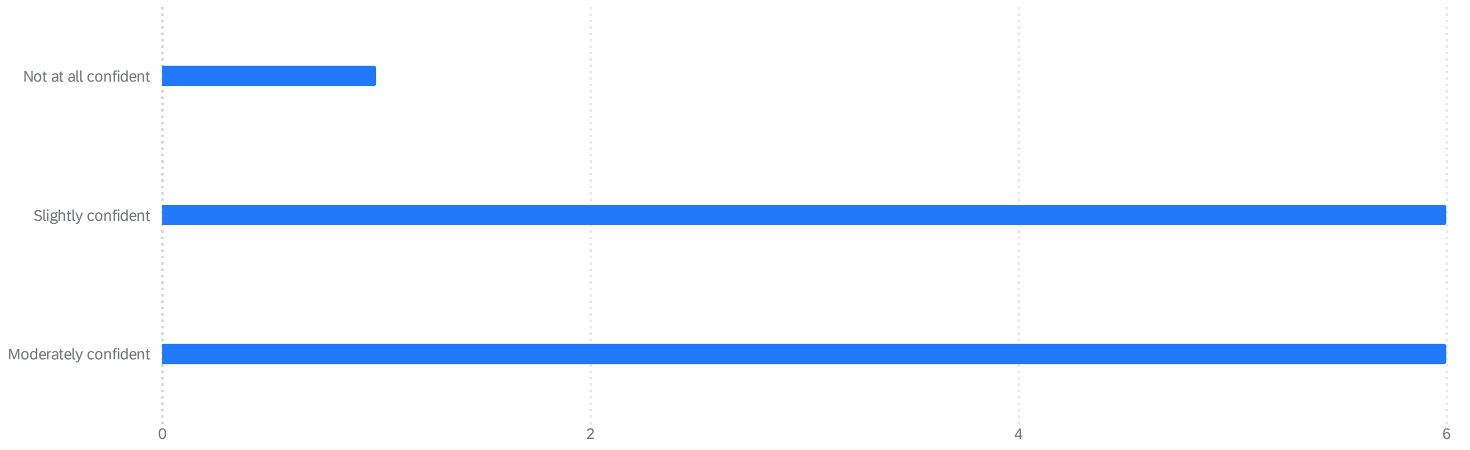

How confident are you in performing ambulatory preoperative assessment? 13

Q7 - How confident are you in performing ambulatory preoperative assessment?	Count	Count
Not at all confident	8%	1
Slightly confident	46%	6
Moderately confident	46%	6

How confident are you in performing ambulatory preoperative assessment? 13

Q7 - How confident are you in performing ambulatory preoperative assessment?	Average (Q7 - How confident are you in performing ambulatory preoperative assessment?)	Minimum (Q7 - How confident are you in performing ambulatory preoperative assessment?)	Maximum (Q7 - How confident are you in performing ambulatory preoperative assessment?)	Count
Moderately confident	3.00	3.00	3.00	6
Not at all confident	1.00	1.00	1.00	1
Slightly confident	2.00	2.00	2.00	6

How confident are you in performing inpatient preoperative assessment? 13

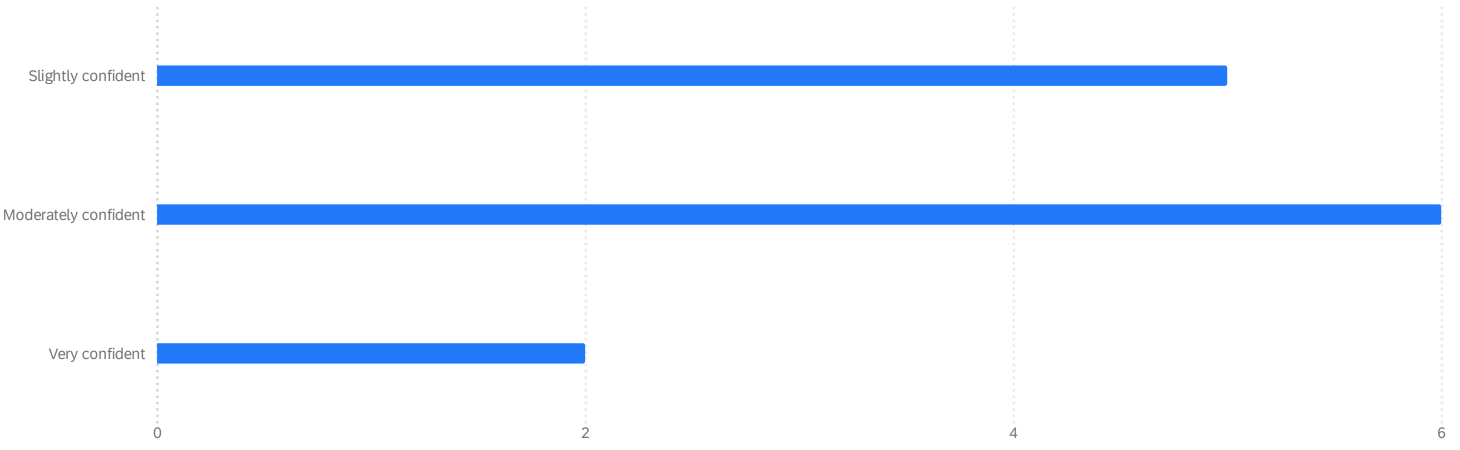

How confident are you in performing inpatient preoperative assessment? 13

Q8 - How confident are you in performing inpatient preoperative assessment?	Count	Count
Slightly confident	38%	5
Moderately confident	46%	6
Very confident	15%	2

How confident are you in performing inpatient preoperative assessment? 13

Q8 - How confident are you in performing inpatient preoperative assessment?	Average (Q8 - How confident are you in performing inpatient preoperative assessment?)	Minimum (Q8 - How confident are you in performing inpatient preoperative assessment?)	Maximum (Q8 - How confident are you in performing inpatient preoperative assessment?)	Count
Moderately confident	3.00	3.00	3.00	6
Slightly confident	2.00	2.00	2.00	5
Very confident	4.00	4.00	4.00	2

How confident are you in applying the ACC/AHA guidelines for perioperative cardiovascular evaluation? 13

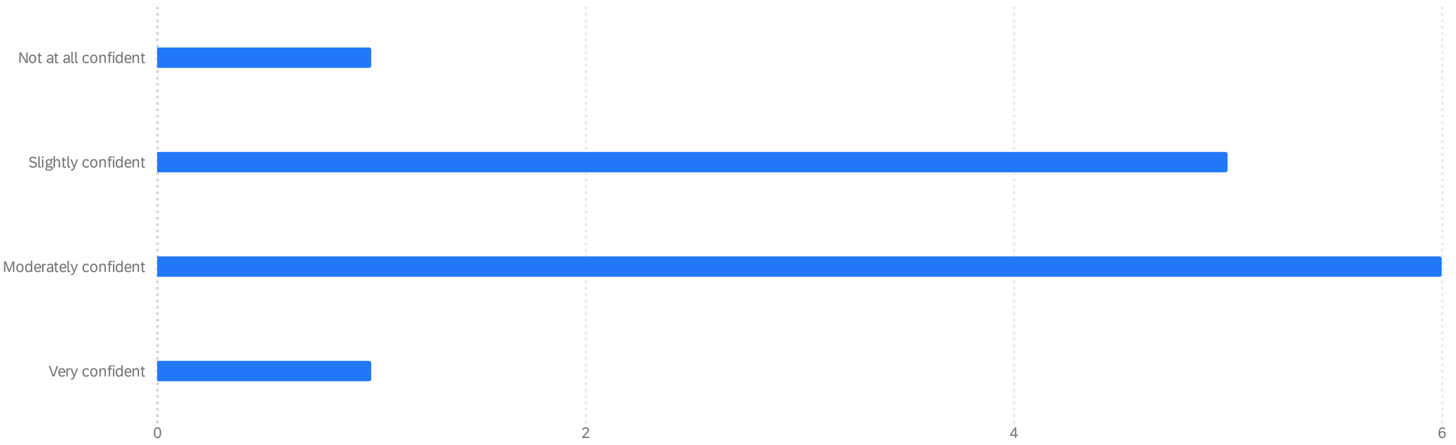

How confident are you in applying the ACC/AHA guidelines for perioperative cardiovascular evaluation? 13

Q9 - How confident are you in applying the ACC/AHA guidelines for perioperative cardiovascular evaluation?	Count	Count
Not at all confident	8%	1
Slightly confident	38%	5
Moderately confident	46%	6
Very confident	8%	1

How confident are you in applying the ACC/AHA guidelines for perioperative cardiovascular evaluation? 13

Q9 - How confident are you in applying the ACC/AHA guidelines for perioperative cardiovascular evaluation?	Average (Q9 - How confident are you in applying the ACC/AHA guidelines for perioperative cardiovascular evaluation?)	Minimum (Q9 - How confident are you in applying the ACC/AHA guidelines for perioperative cardiovascular evaluation?)	Maximum (Q9 - How confident are you in applying the ACC/AHA guidelines for perioperative cardiovascular evaluation?)	Count
Moderately confident	3.00	3.00	3.00	6
Not at all confident	1.00	1.00	1.00	1
Slightly confident	2.00	2.00	2.00	5
Very confident	4.00	4.00	4.00	1

How confident are you in managing patients undergoing bariatric surgery preoperatively? 13

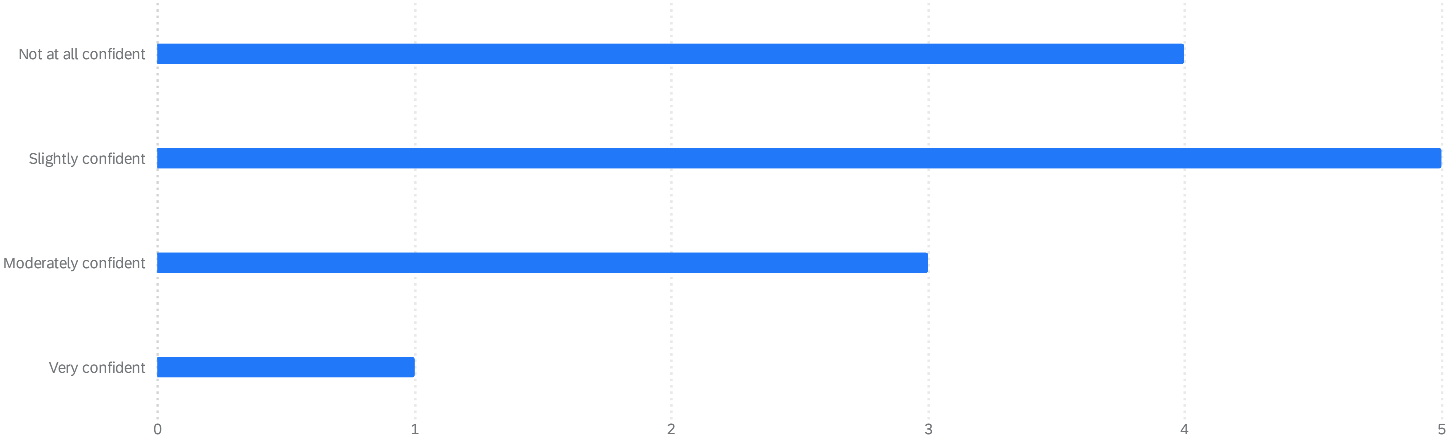

How confident are you in managing patients undergoing bariatric surgery preoperatively? 13

Q10 - How confident are you in managing patients undergoing bariatric surgery preoperatively?	Count	Count
Not at all confident	31%	4
Slightly confident	38%	5
Moderately confident	23%	3
Very confident	8%	1

How confident are you in managing patients undergoing bariatric surgery preoperatively? 13

Q10 - How confident are you in managing patients undergoing bariatric surgery preoperatively?	Average (Q10 - How confident are you in managing patients undergoing bariatric surgery preoperatively?)	Minimum (Q10 - How confident are you in managing patients undergoing bariatric surgery preoperatively?)	Maximum (Q10 - How confident are you in managing patients undergoing bariatric surgery preoperatively?)	Count
Moderately confident	3.00	3.00	3.00	3
Not at all confident	1.00	1.00	1.00	4
Slightly confident	2.00	2.00	2.00	5
Very confident	4.00	4.00	4.00	1

How confident are you in managing patients with chronic kidney disease preoperatively? 13

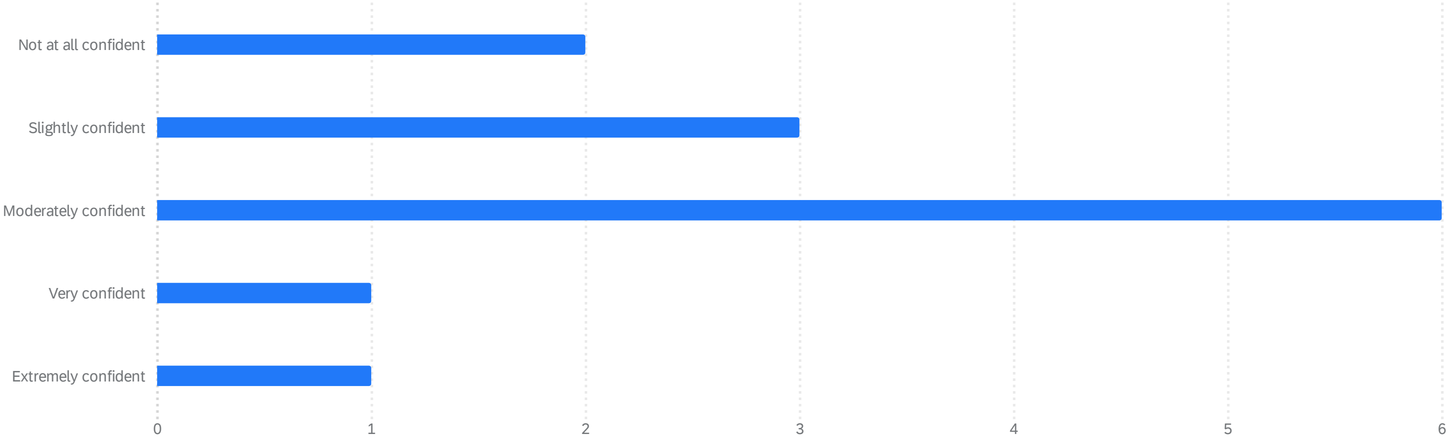

How confident are you in managing patients with chronic kidney disease preoperatively? 13

Q11 - How confident are you in managing patients with chronic kidney disease preoperatively?	Count	Count
Not at all confident	15%	2
Slightly confident	23%	3
Moderately confident	46%	6
Very confident	8%	1
Extremely confident	8%	1

How confident are you in managing patients with chronic kidney disease preoperatively? 13

Q11 - How confident are you in managing patients with chronic kidney disease preoperatively?	Average (Q11 - How confident are you in managing patients with chronic kidney disease preoperatively?)	Minimum (Q11 - How confident are you in managing patients with chronic kidney disease preoperatively?)	Maximum (Q11 - How confident are you in managing patients with chronic kidney disease preoperatively?)	Count
Extremely confident	5.00	5.00	5.00	1
Moderately confident	3.00	3.00	3.00	6
Not at all confident	1.00	1.00	1.00	2
Slightly confident	2.00	2.00	2.00	3
Very confident	4.00	4.00	4.00	1

How confident are you in managing patients with liver disease preoperatively? 13

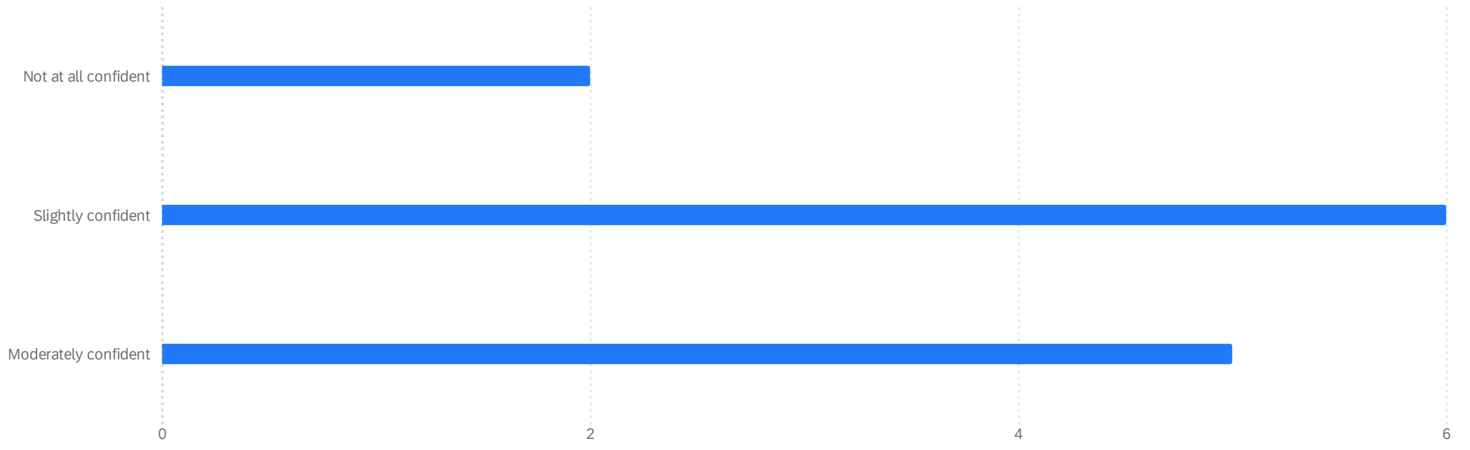

How confident are you in managing patients with liver disease preoperatively? 13

Q12 - How confident are you in managing patients with liver disease preoperatively?	Count	Count
Not at all confident	15%	2
Slightly confident	46%	6
Moderately confident	38%	5

How confident are you in managing patients with liver disease preoperatively? 13

Q12 - How confident are you in managing patients with liver disease preoperatively?	Average (Q12 - How confident are you in managing patients with liver disease preoperatively?)	Minimum (Q12 - How confident are you in managing patients with liver disease preoperatively?)	Maximum (Q12 - How confident are you in managing patients with liver disease preoperatively?)	Count
Moderately confident	3.00	3.00	3.00	5
Not at all confident	1.00	1.00	1.00	2
Slightly confident	2.00	2.00	2.00	6

How confident are you in managing patients with rheumatologic conditions (e.g., RA) preoperatively? 13

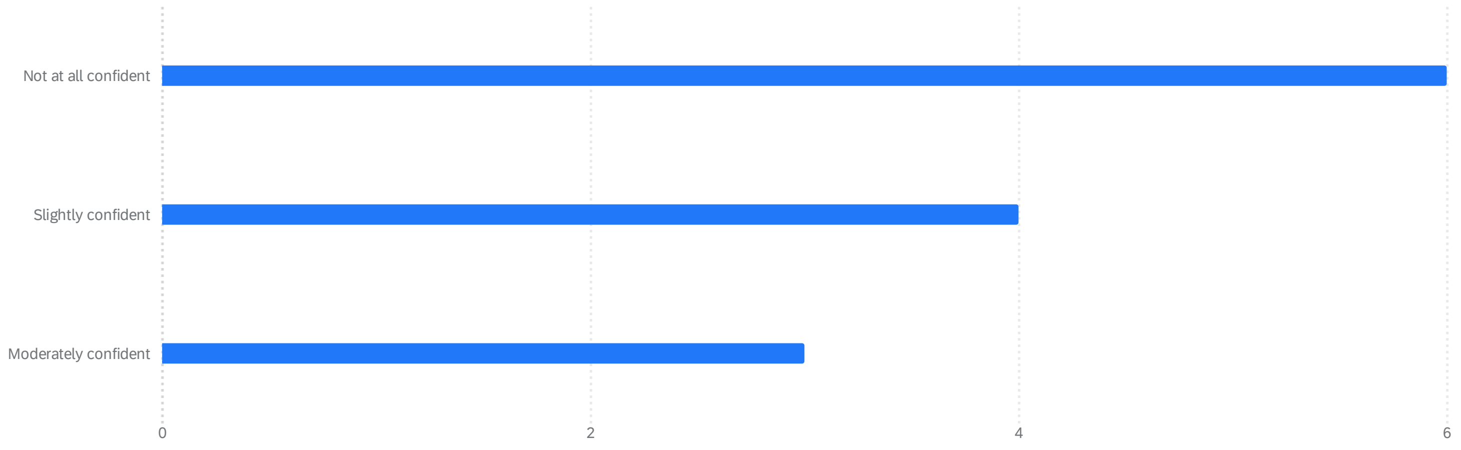

How confident are you in managing patients with rheumatologic conditions (e.g., RA) preoperatively? 13

Q13 - How confident are you in managing patients with rheumatologic conditions (e.g., RA) preoperatively?	Count	Count
Not at all confident	46%	6
Slightly confident	31%	4
Moderately confident	23%	3

How confident are you in managing patients with rheumatologic conditions (e.g., RA) preoperatively? 13

Q13 - How confident are you in managing patients with rheumatologic conditions (e.g., RA) preoperatively?	Average (Q13 - How confident are you in managing patients with rheumatologic conditions (e.g., RA) preoperatively?)	Minimum (Q13 - How confident are you in managing patients with rheumatologic conditions (e.g., RA) preoperatively?)	Maximum (Q13 - How confident are you in managing patients with rheumatologic conditions (e.g., RA) preoperatively?)	Count
Moderately confident	3.00	3.00	3.00	3
Not at all confident	1.00	1.00	1.00	6
Slightly confident	2.00	2.00	2.00	4

Guidelines recommend utilizing risk calculators to estimate risk of major adverse cardiac events (MACE). What is the percentage cut off? 11

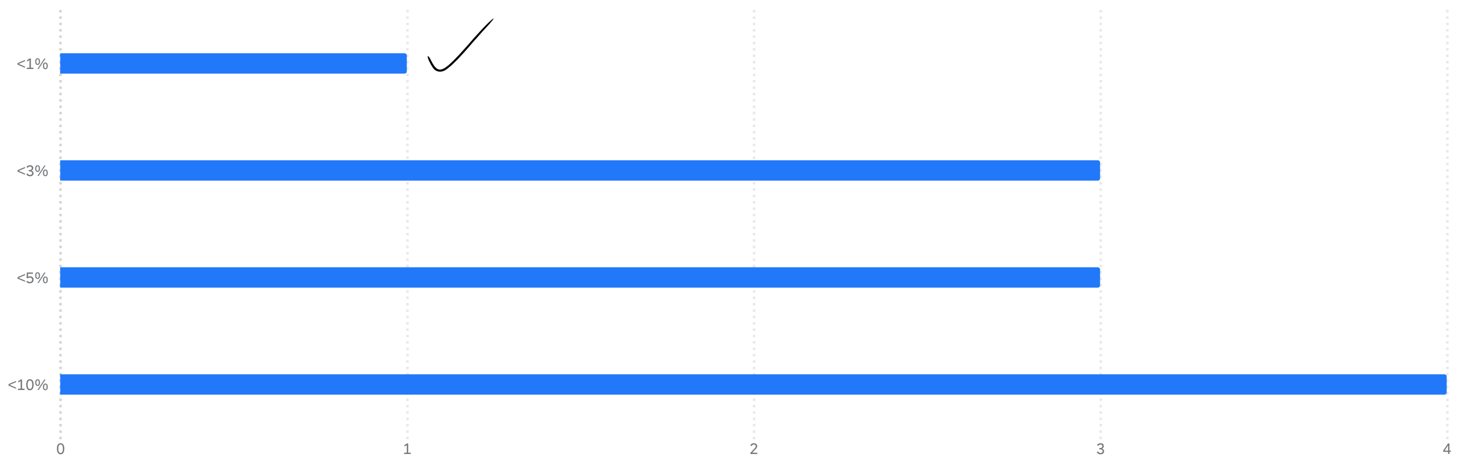

Guidelines recommend utilizing risk calculators to estimate risk of major adverse cardiac events (MACE). What is the percentage cut off? 11

Q14 - Guidelines recommend utilizing risk calculators to estimate risk of major adverse cardiac events (MACE). What is the percentage cut off?	Count	Count
<1%	9%	1
<3%	27%	3
<5%	27%	3
<10%	36%	4

Guidelines recommend utilizing risk calculators to estimate risk of major adverse cardiac events (MACE). What is the percentage cut off? 11

Q14 - Guidelines recommend utilizing risk calculators to estimate risk of major adverse cardiac events (MACE). What is the percentage cut off?	Average (Q14 - Guidelines recommend utilizing risk calculators to estimate risk of major adverse cardiac events (MACE). What is the percentage cut off?)	Minimum (Q14 - Guidelines recommend utilizing risk calculators to estimate risk of major adverse cardiac events (MACE). What is the percentage cut off?)	Maximum (Q14 - Guidelines recommend utilizing risk calculators to estimate risk of major adverse cardiac events (MACE). What is the percentage cut off?)	Count
<1%	1.00	1.00	1.00	1
<10%	4.00	4.00	4.00	4
<3%	2.00	2.00	2.00	3
<5%	3.00	3.00	3.00	3

For patients who received bare metal stents, which of the following is a TRUE statement? 11

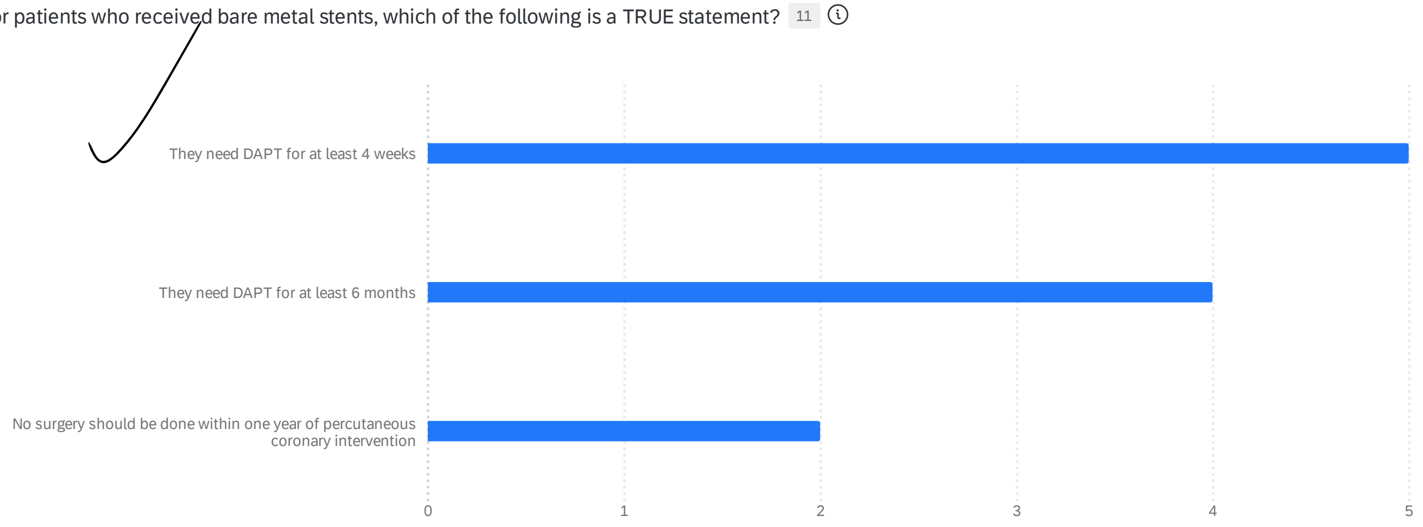

For patients who received bare metal stents, which of the following is a TRUE statement? 11

Q15 - For patients who received bare metal stents, which of the following is a TRUE statement?	Count	Count
They need DAPT for at least 4 weeks	45%	5
They need DAPT for at least 6 months	36%	4
No surgery should be done within one year of percutaneous coronary intervention	18%	2

For patients who received bare metal stents, which of the following is a TRUE statement? 11

Q15 - For patients who received bare metal stents, which of the following is a TRUE statement?	Average (Q15 - For patients who received bare metal stents, which of the following is a TRUE statement?)	Minimum (Q15 - For patients who received bare metal stents, which of the following is a TRUE statement?)	Maximum (Q15 - For patients who received bare metal stents, which of the following is a TRUE statement?)	Count
No surgery should be done within one year of percutaneous coronary intervention	3.00	3.00	3.00	2
They need DAPT for at least 4 weeks	1.00	1.00	1.00	5
They need DAPT for at least 6 months	2.00	2.00	2.00	4

For patients who received drug-eluting stents, what is the recommended duration for dual anti-platelet therapy? 11

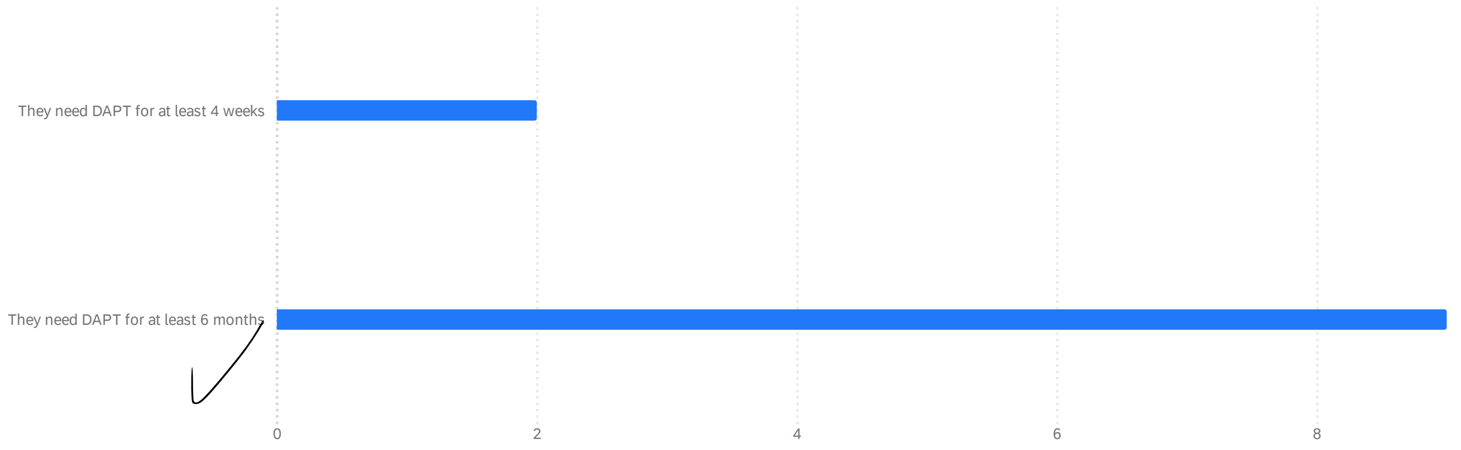

For patients who received drug-eluting stents, what is the recommended duration for dual anti-platelet therapy? 11

Q16 - For patients who received drug-eluting stents, what is the recommended duration for dual anti-platelet therapy?	Count	Count
They need DAPT for at least 4 weeks	18%	2
They need DAPT for at least 6 months	82%	9

For patients who received drug-eluting stents, what is the recommended duration for dual anti-platelet therapy? 11

Q16 - For patients who received drug-eluting stents, what is the recommended duration for dual anti-platelet therapy?	Average (Q16 - For patients who received drug-eluting stents, what is the recommended duration for dual anti-platelet therapy?)	Minimum (Q16 - For patients who received drug-eluting stents, what is the recommended duration for dual anti-platelet therapy?)	Maximum (Q16 - For patients who received drug-eluting stents, what is the recommended duration for dual anti-platelet therapy?)	Count
They need DAPT for at least 4 weeks	1.00	1.00	1.00	2
They need DAPT for at least 6 months	2.00	2.00	2.00	9

Which statement is true? 11

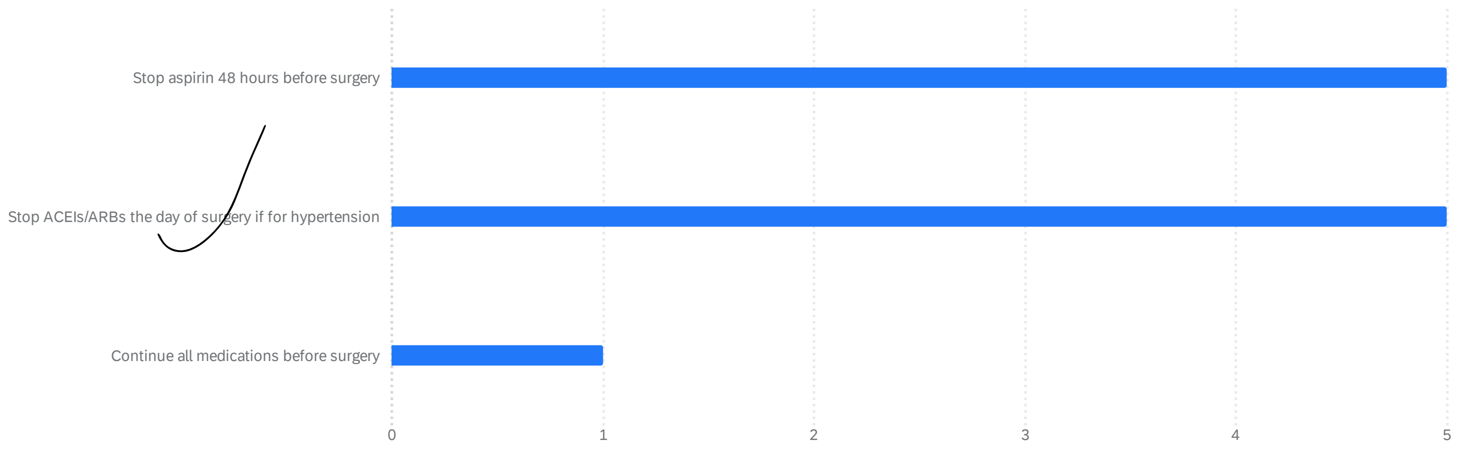

Which statement is true? 11

Q17 - Which statement is true?	Count	Count
Stop aspirin 48 hours before surgery	45%	5
Stop ACEIs/ARBs the day of surgery if for hypertension	45%	5
Continue all medications before surgery	9%	1

Which statement is true? 11

Q17 - Which statement is true?	Average (Q17 - Which statement is true?)	Minimum (Q17 - Which statement is true?)	Maximum (Q17 - Which statement is true?)	Count
Continue all medications before surgery	4.00	4.00	4.00	1
Stop ACEIs/ARBs the day of surgery if for hypertension	3.00	3.00	3.00	5
Stop aspirin 48 hours before surgery	1.00	1.00	1.00	5

Which set of patients require stress dose steroids to prevent adrenal crisis during surgery? 11

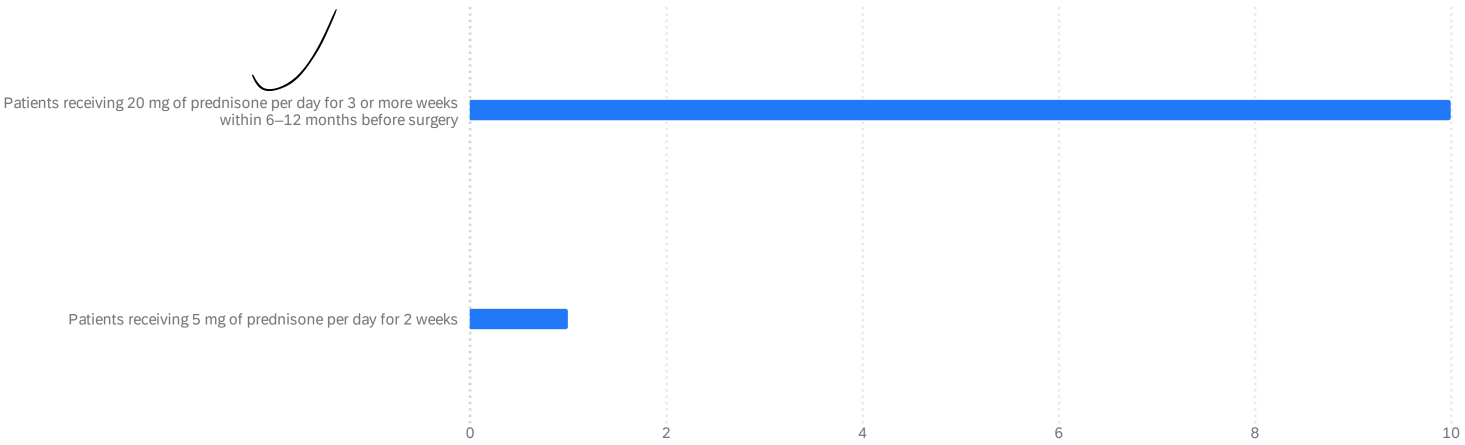

Which set of patients require stress dose steroids to prevent adrenal crisis during surgery? 11

Q18 - Which set of patients require stress dose steroids to prevent adrenal crisis during surgery?	Count	Count
Patients receiving 20 mg of prednisone per day for 3 or more weeks within 6–12 months before surgery	91%	10
Patients receiving 5 mg of prednisone per day for 2 weeks	9%	1

Which set of patients require stress dose steroids to prevent adrenal crisis during surgery? 11

Q18 - Which set of patients require stress dose steroids to prevent adrenal crisis during surgery?	Average (Q18 - Which set of patients require stress dose steroids to prevent adrenal crisis during surgery?)	Minimum (Q18 - Which set of patients require stress dose steroids to prevent adrenal crisis during surgery?)	Maximum (Q18 - Which set of patients require stress dose steroids to prevent adrenal crisis during surgery?)	Count
Patients receiving 20 mg of prednisone per day for 3 or more weeks within 6–12 months before surgery	2.00	2.00	2.00	10
Patients receiving 5 mg of prednisone per day for 2 weeks	3.00	3.00	3.00	1

Which of the following statements are true regarding insulin management prior to surgery for patients with type I diabetes? (Select all that apply) 11

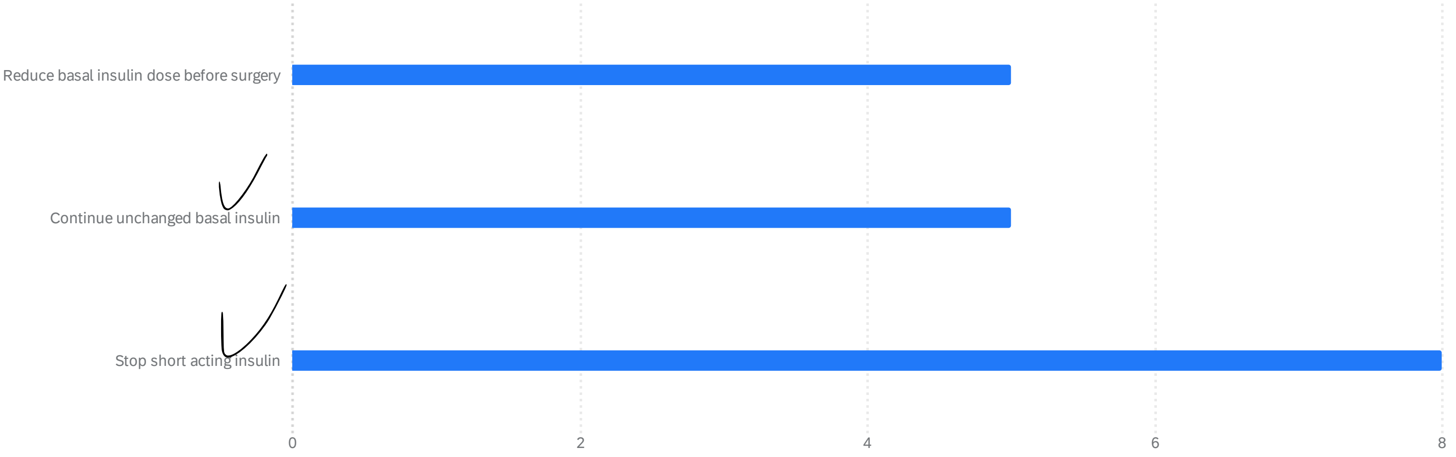

Which of the following statements are true regarding insulin management prior to surgery for patients with type I diabetes? (Select all that apply) 11

Q19 - Which of the following statements are true regarding insulin management prior to surgery for patients with type I diabetes? (Select all that apply)	Count	Count
Reduce basal insulin dose before surgery	45%	5
Continue unchanged basal insulin	45%	5
Stop short acting insulin	73%	8

Which statement regarding SGLT2 inhibitors is true? (Select all that apply) 11

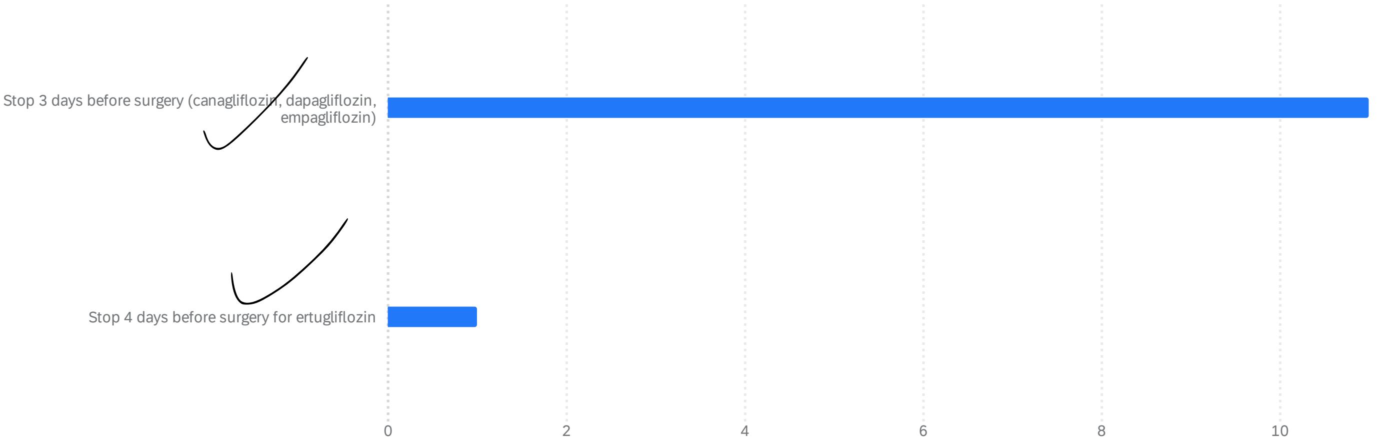

Which statement regarding SGLT2 inhibitors is true? (Select all that apply) 11

Q20 - Which statement regarding SGLT2 inhibitors is true? (Select all that apply)	Count	Count
Stop 3 days before surgery (canagliflozin, dapagliflozin, empagliflozin)	100%	11
Stop 4 days before surgery for ertugliflozin	9%	1

Which rheumatologic agents are safe to continue before surgery? (Select all that apply) 10

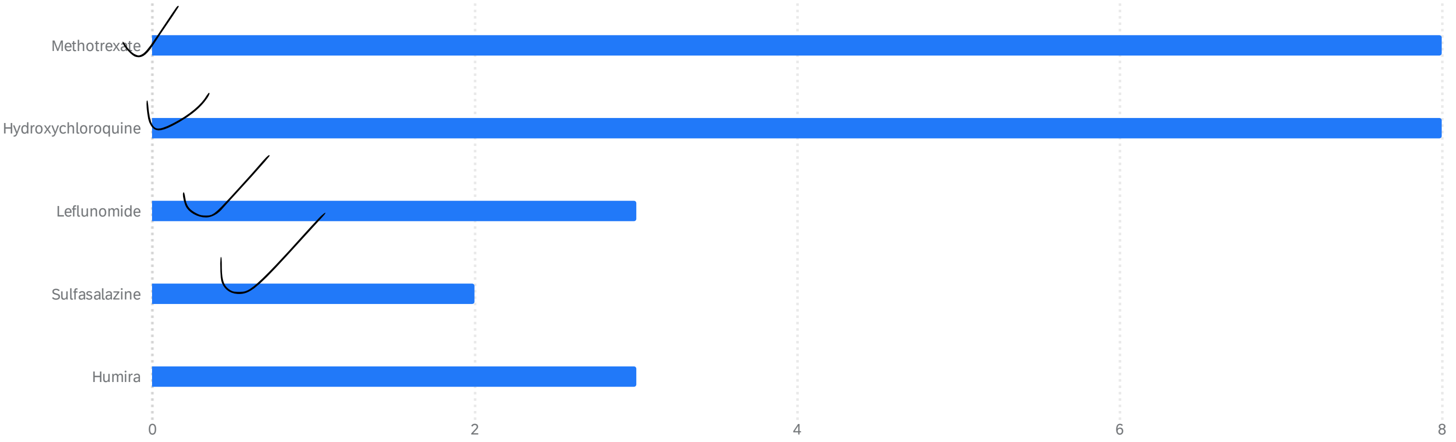

Which rheumatologic agents are safe to continue before surgery? (Select all that apply) 10

Q21 - Which rheumatologic agents are safe to continue before surgery? (Select all that apply)	Count	Count
Methotrexate	80%	8
Hydroxychloroquine	80%	8
Leflunomide	30%	3
Sulfasalazine	20%	2
Humira	30%	3

Which statement regarding bridging is true? (Select all that apply) 10

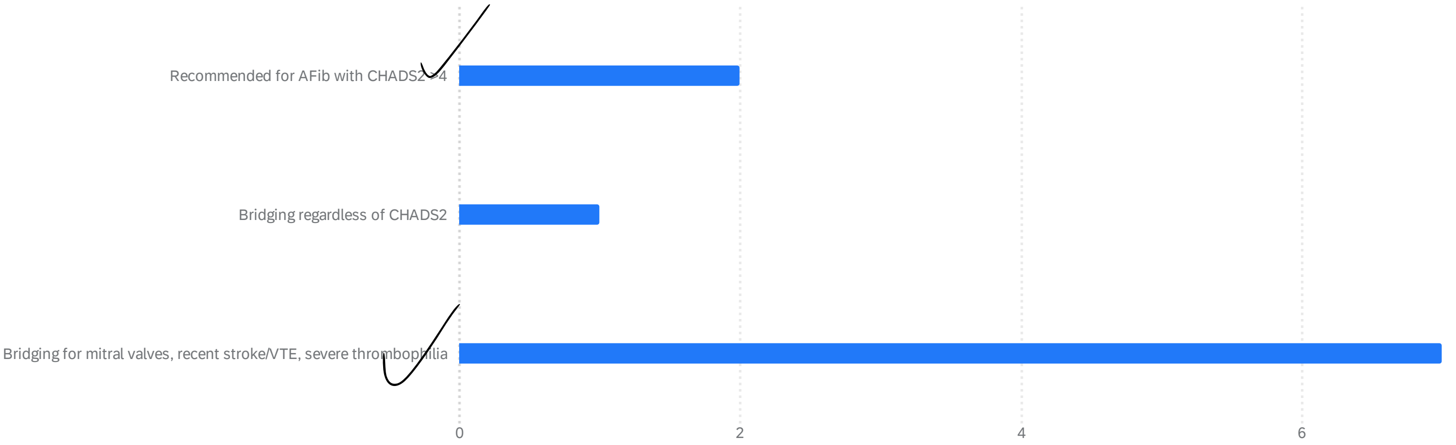

Which statement regarding bridging is true? (Select all that apply) 10

Q22 - Which statement regarding bridging is true? (Select all that apply)	Count	Count
Recommended for AFib with CHADS2 >4	20%	2
Bridging regardless of CHADS2	10%	1
Bridging for mitral valves, recent stroke/VTE, severe thrombophilia	70%	7

Which statement regarding bridging is true? (Select all that apply) 10

Q22 - Which statement regarding bridging is true? (Select all that apply)	Average (Q22 - Which statement regarding bridging is true? (Select all that apply))	Minimum (Q22 - Which statement regarding bridging is true? (Select all that apply))	Maximum (Q22 - Which statement regarding bridging is true? (Select all that apply))	Count
Bridging for mitral valves, recent stroke/VTE, severe thrombophilia	5.00	5.00	5.00	7
Bridging regardless of CHADS2	3.00	3.00	3.00	1
Recommended for AFib with CHADS2 >4	2.00	2.00	2.00	2

For patients on warfarin, you would check INR 7–10 days before the procedure. Which of the following is TRUE? (Select all that apply) 11

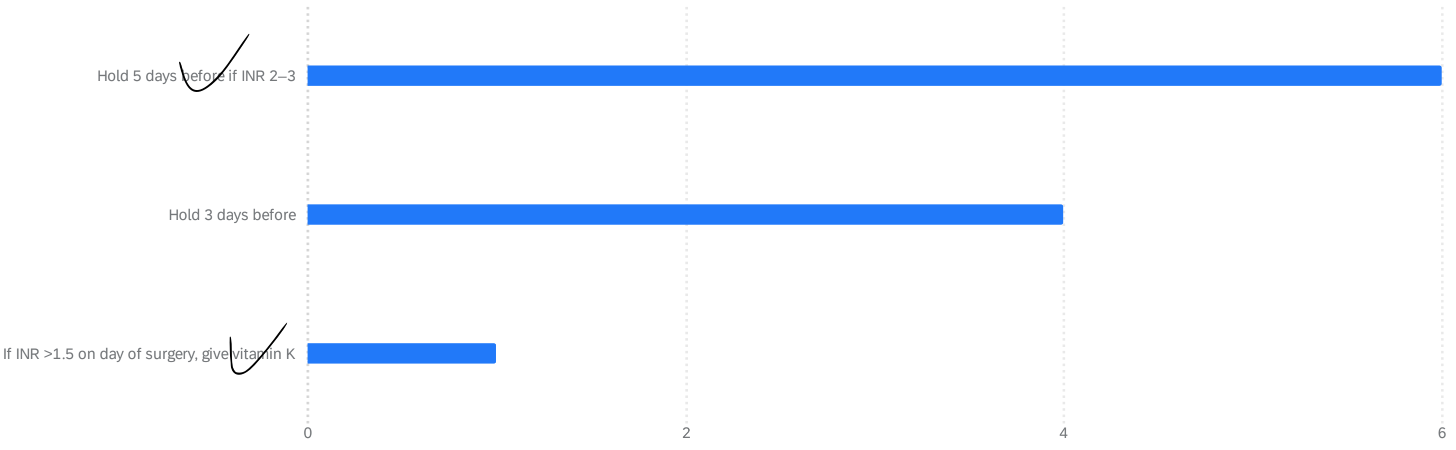

For patients on warfarin, you would check INR 7–10 days before the procedure. Which of the following is TRUE? (Select all that apply) 11

Q23 - For patients on warfarin, you would check INR 7–10 days before the procedure. Which of the following is TRUE? (Select all that apply)	Count	Count
Hold 5 days before if INR 2–3	55%	6
Hold 3 days before	36%	4
If INR >1.5 on day of surgery, give vitamin K	9%	1

For patients on warfarin, you would check INR 7–10 days before the procedure. Which of the following is TRUE? (Select all that apply) 11

Q23 - For patients on warfarin, you would check INR 7–10 days before the procedure. Which of the following is TRUE? (Select all that apply)	Average (Q23 - For patients on warfarin, you would check INR 7–10 days before the procedure. Which of the following is TRUE? (Select all that apply))	Minimum (Q23 - For patients on warfarin, you would check INR 7–10 days before the procedure. Which of the following is TRUE? (Select all that apply))	Maximum (Q23 - For patients on warfarin, you would check INR 7–10 days before the procedure. Which of the following is TRUE? (Select all that apply))	Count
Hold 3 days before	2.00	2.00	2.00	4
Hold 5 days before if INR 2–3	1.00	1.00	1.00	6
If INR >1.5 on day of surgery, give vitamin K	5.00	5.00	5.00	1

How do you restart warfarin after low-moderate bleeding risk procedure? 11

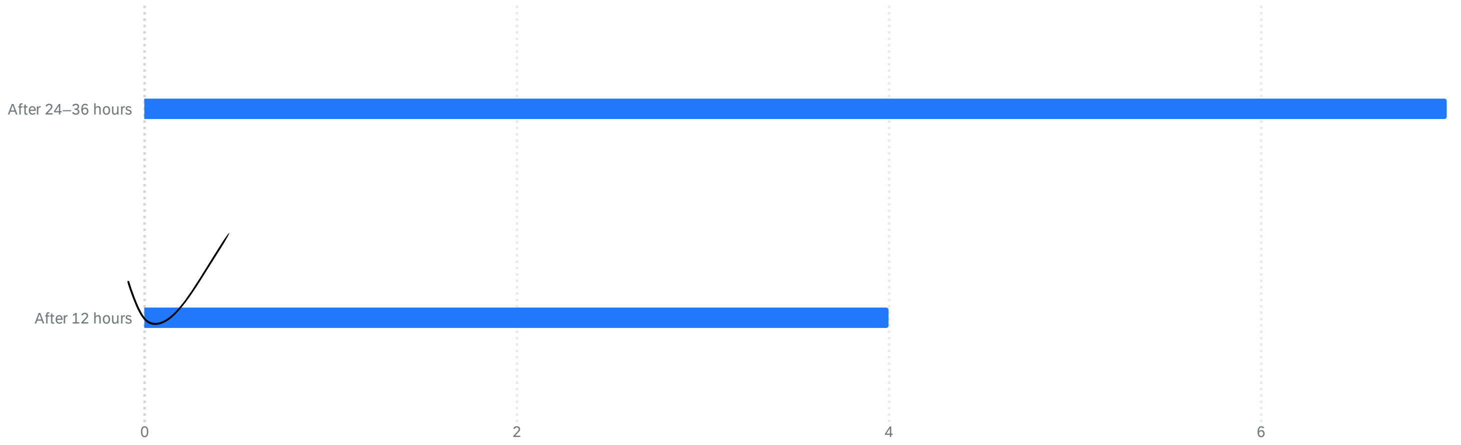

How do you restart warfarin after low-moderate bleeding risk procedure? 11

Q24 - How do you restart warfarin after low-moderate bleeding risk procedure?	Count	Count
After 24–36 hours	64%	7
After 12 hours	36%	4

How do you restart warfarin after low-moderate bleeding risk procedure? 11

Q24 - How do you restart warfarin after low-moderate bleeding risk procedure?	Average (Q24 - How do you restart warfarin after low-moderate bleeding risk procedure?)	Minimum (Q24 - How do you restart warfarin after low-moderate bleeding risk procedure?)	Maximum (Q24 - How do you restart warfarin after low-moderate bleeding risk procedure?)	Count
After 12 hours	2.00	2.00	2.00	4
After 24–36 hours	1.00	1.00	1.00	7

Which is true regarding DOACs and normal CrCl? 11

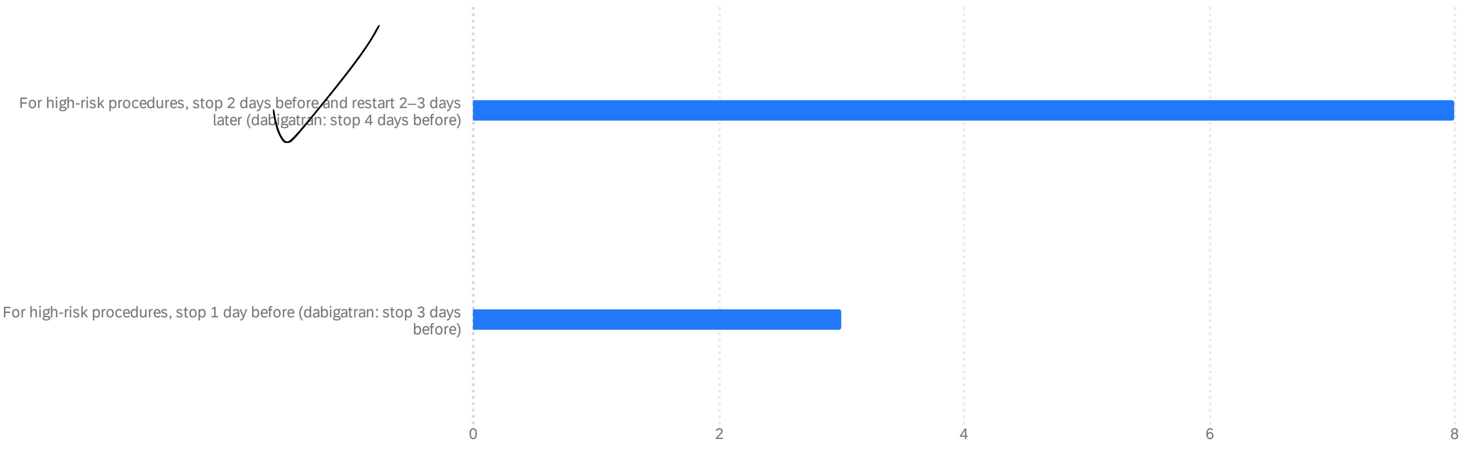

Which is true regarding DOACs and normal CrCl? 11

Q25 - Which is true regarding DOACs and normal CrCl?	Count	Count
For high-risk procedures, stop 2 days before and restart 2–3 days later (dabigatran: stop 4 days before)	73%	8
For high-risk procedures, stop 1 day before (dabigatran: stop 3 days before)	27%	3

Which is true regarding DOACs and normal CrCl? 11

Q25 - Which is true regarding DOACs and normal CrCl?	Average (Q25 - Which is true regarding DOACs and normal CrCl?)	Minimum (Q25 - Which is true regarding DOACs and normal CrCl?)	Maximum (Q25 - Which is true regarding DOACs and normal CrCl?)	Count
For high-risk procedures, stop 1 day before (dabigatran: stop 3 days before)	2.00	2.00	2.00	3
For high-risk procedures, stop 2 days before and restart 2–3 days later (dabigatran: stop 4 days before)	1.00	1.00	1.00	8

What calculator(s) can be used to assess VTE risk in bariatric surgery patients? (Select all that apply) 7

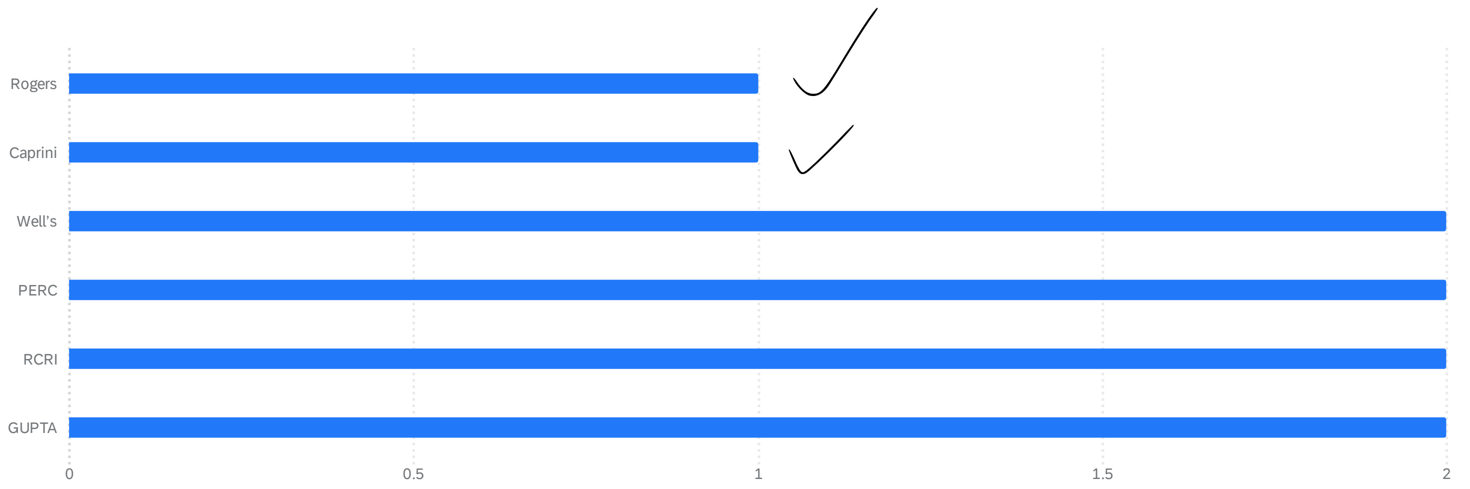

What calculator(s) can be used to assess VTE risk in bariatric surgery patients? (Select all that apply) 7

Q26 - What calculator(s) can be used to assess VTE risk in bariatric surgery patients? (Select all that apply)	Count	Count
Rogers	14%	1
Caprini	14%	1
Well's	29%	2
PERC	29%	2
RCRI	29%	2
GUPTA	29%	2
