# Supplementary material for: Escaping the OR: a pilot study of a Jigsaw-based workshop to teach preoperative assessment in internal medicine residency
Source: BMC Med Educ. 2026 May 16;26:1097. doi: 10.1186/s12909-026-09419-w (PMC13348809; doi:10.1186/s12909-026-09419-w)
Supplement: Supplementary file 1 — Supplementary Material 1. [file 12909_2026_9419_MOESM1_ESM.zip › Pre Intervention.pdf]

PRE Intervention Pre-OP Group Survey / Page 1

Responses: 8

Age: 8

Age:

29
34
28
32
30
39
29
30

Gender 8

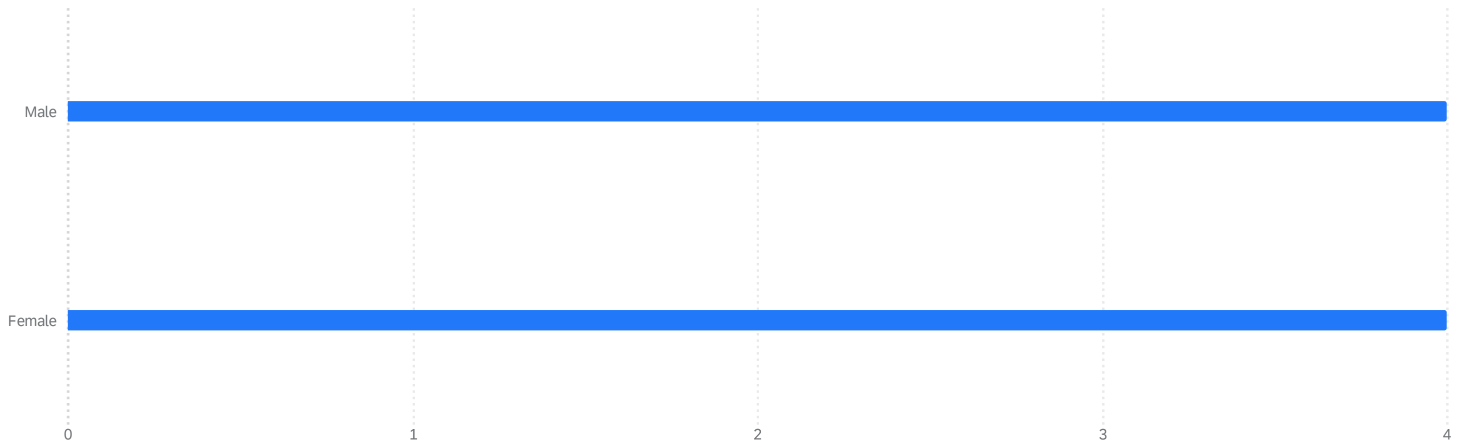

Gender 8

Q2 - Gender	Count	Count
Male	50%	4
Female	50%	4

Gender 8

Q2 - Gender	Average (Q2 - Gender)	Minimum (Q2 - Gender)	Maximum (Q2 - Gender)	Count
Female	2.00	2.00	2.00	4
Male	1.00	1.00	1.00	4

Ethnicity (Check all that apply): 8

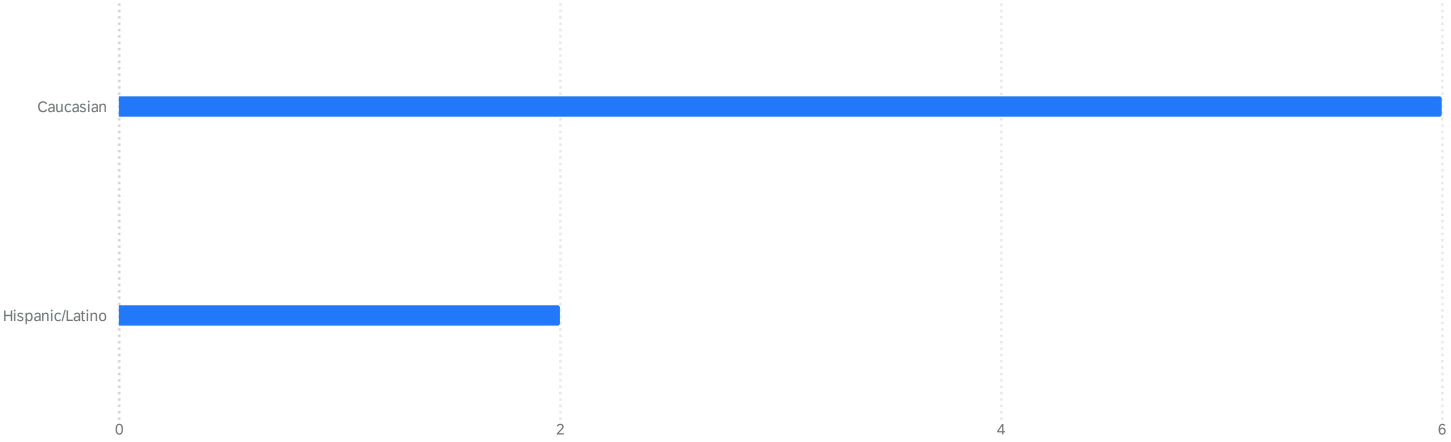

Ethnicity (Check all that apply): 8

Q3 - Ethnicity (Check all that apply):	Count	Count
Caucasian	75%	6
Hispanic/Latino	25%	2

Please indicate your PGY year: 8

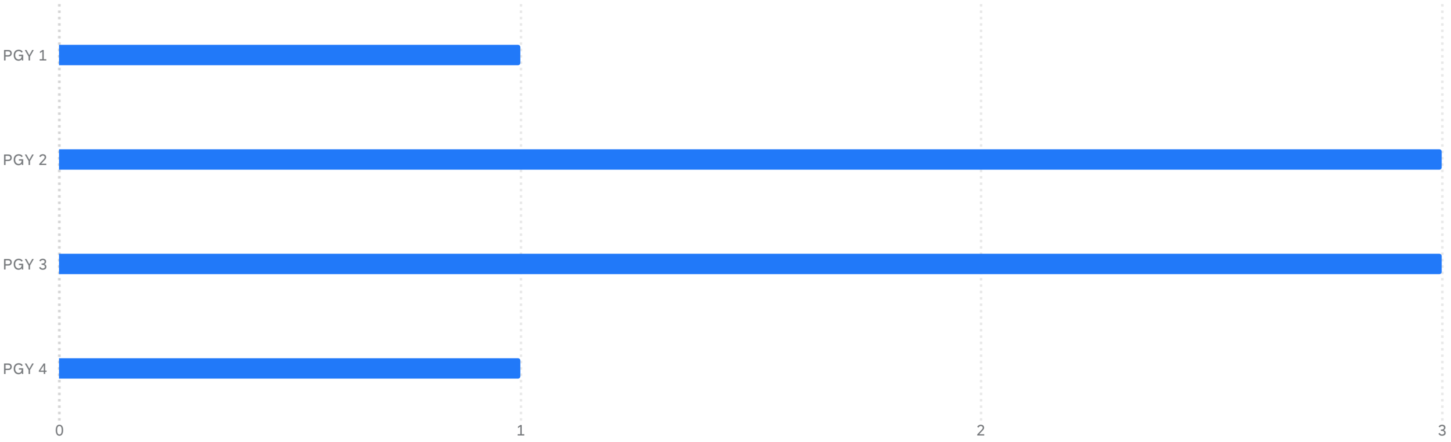

Please indicate your PGY year: 8

Q4 - Please indicate your PGY year:	Count	Count
PGY 1	13%	1
PGY 2	38%	3
PGY 3	38%	3
PGY 4	13%	1

Please indicate your PGY year: 8

Q4 - Please indicate your PGY year:	Average (Q4 - Please indicate your PGY year:)	Minimum (Q4 - Please indicate your PGY year:)	Maximum (Q4 - Please indicate your PGY year:)	Count
PGY 1	1.00	1.00	1.00	1
PGY 2	2.00	2.00	2.00	3
PGY 3	3.00	3.00	3.00	3
PGY 4	4.00	4.00	4.00	1

Have you received training in ambulatory preoperative assessment in the following settings? 8

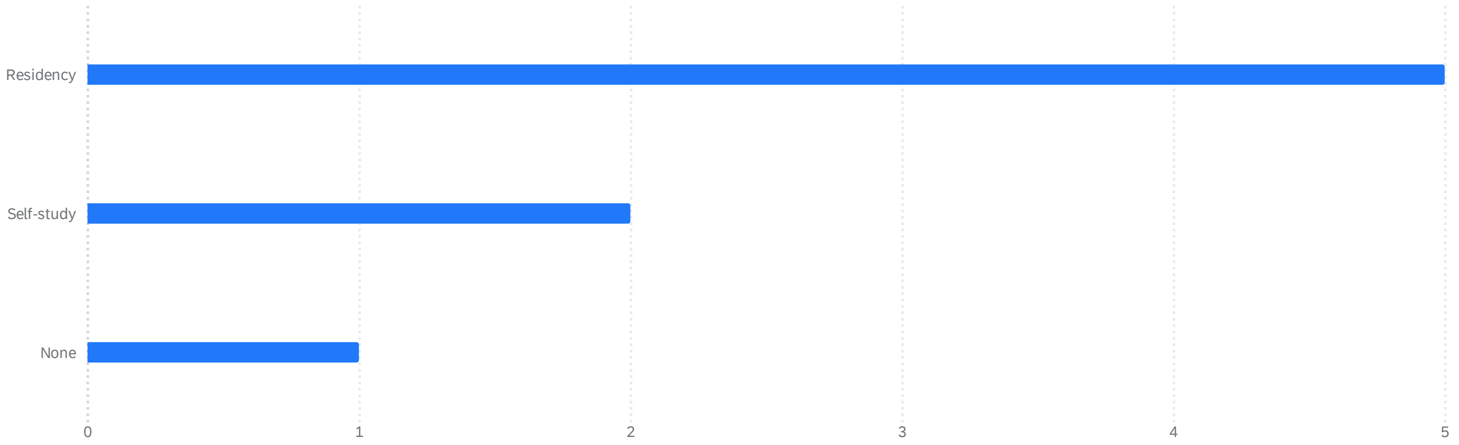

Have you received training in ambulatory preoperative assessment in the following settings? 8

Q5 - Have you received training in ambulatory preoperative assessment in the following settings?	Count	Count
Residency	63%	5
Self-study	25%	2
None	13%	1

Have you received training in ambulatory preoperative assessment in the following settings? 8

Q5 - Have you received training in ambulatory preoperative assessment in the following settings?	Average (Q5 - Have you received training in ambulatory preoperative assessment in the following settings?)	Minimum (Q5 - Have you received training in ambulatory preoperative assessment in the following settings?)	Maximum (Q5 - Have you received training in ambulatory preoperative assessment in the following settings?)	Count
None	6.00	6.00	6.00	1
Residency	4.00	4.00	4.00	5
Self-study	5.00	5.00	5.00	2

Have you received training in inpatient preoperative assessment in the following settings? 8

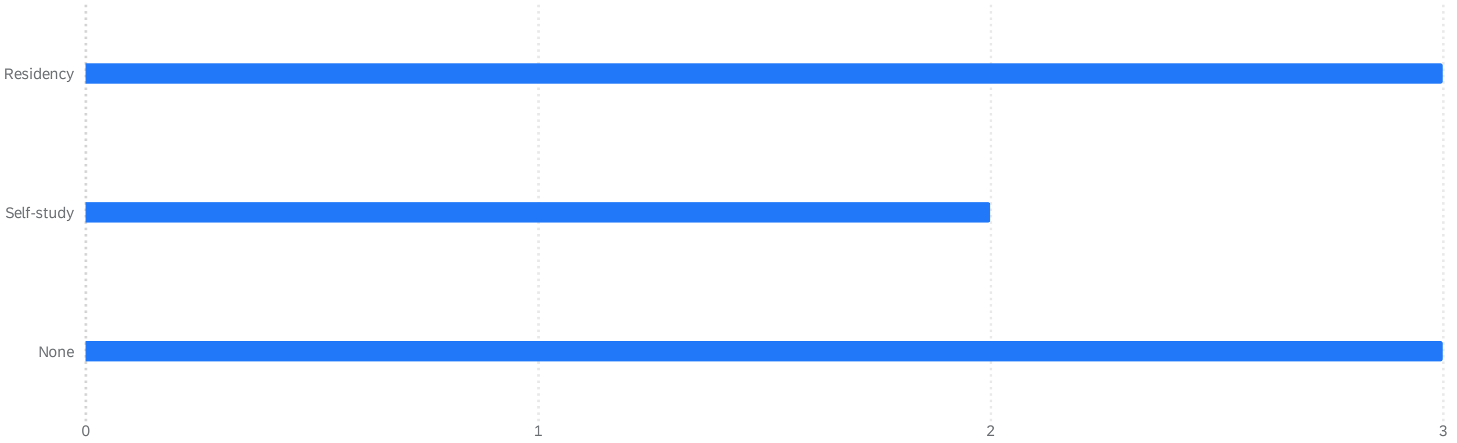

Have you received training in inpatient preoperative assessment in the following settings? 8

Q6 - Have you received training in inpatient preoperative assessment in the following settings?	Count	Count
Residency	38%	3
Self-study	25%	2
None	38%	3

Have you received training in inpatient preoperative assessment in the following settings? 8

Q6 - Have you received training in inpatient preoperative assessment in the following settings?	Average (Q6 - Have you received training in inpatient preoperative assessment in the following settings?)	Minimum (Q6 - Have you received training in inpatient preoperative assessment in the following settings?)	Maximum (Q6 - Have you received training in inpatient preoperative assessment in the following settings?)	Count
None	6.00	6.00	6.00	3
Residency	4.00	4.00	4.00	3
Self-study	5.00	5.00	5.00	2

How confident are you in performing ambulatory preoperative assessment? 8

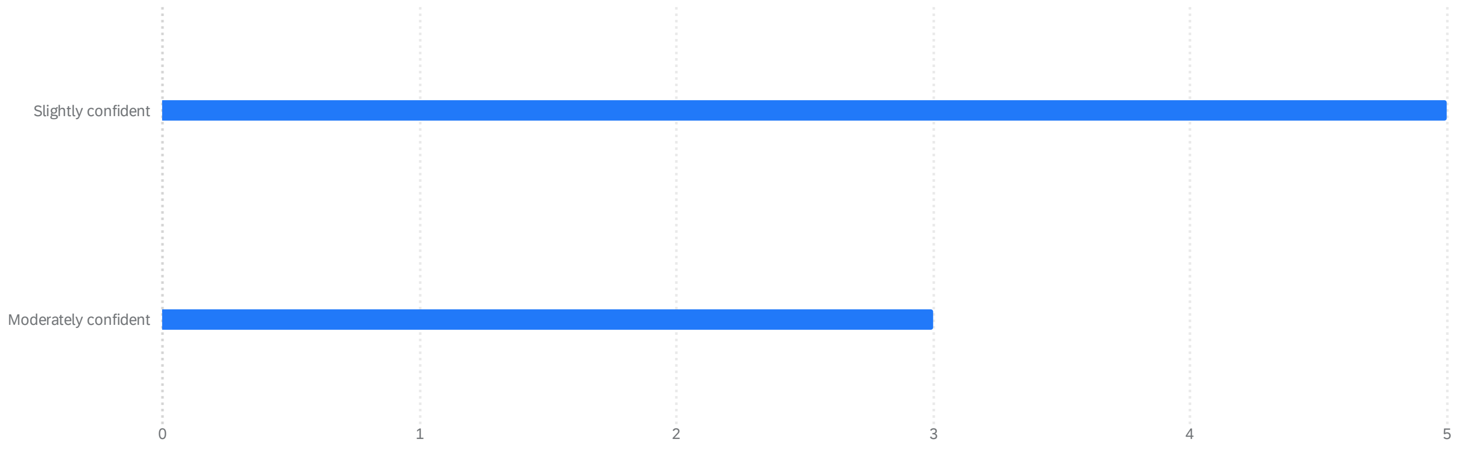

How confident are you in performing ambulatory preoperative assessment? 8

Q7 - How confident are you in performing ambulatory preoperative assessment?	Count	Count
Slightly confident	63%	5
Moderately confident	38%	3

How confident are you in performing ambulatory preoperative assessment? 8

Q7 - How confident are you in performing ambulatory preoperative assessment?	Average (Q7 - How confident are you in performing ambulatory preoperative assessment?)	Minimum (Q7 - How confident are you in performing ambulatory preoperative assessment?)	Maximum (Q7 - How confident are you in performing ambulatory preoperative assessment?)	Count
Moderately confident	3.00	3.00	3.00	3
Slightly confident	2.00	2.00	2.00	5

How confident are you in performing inpatient preoperative assessment? 8

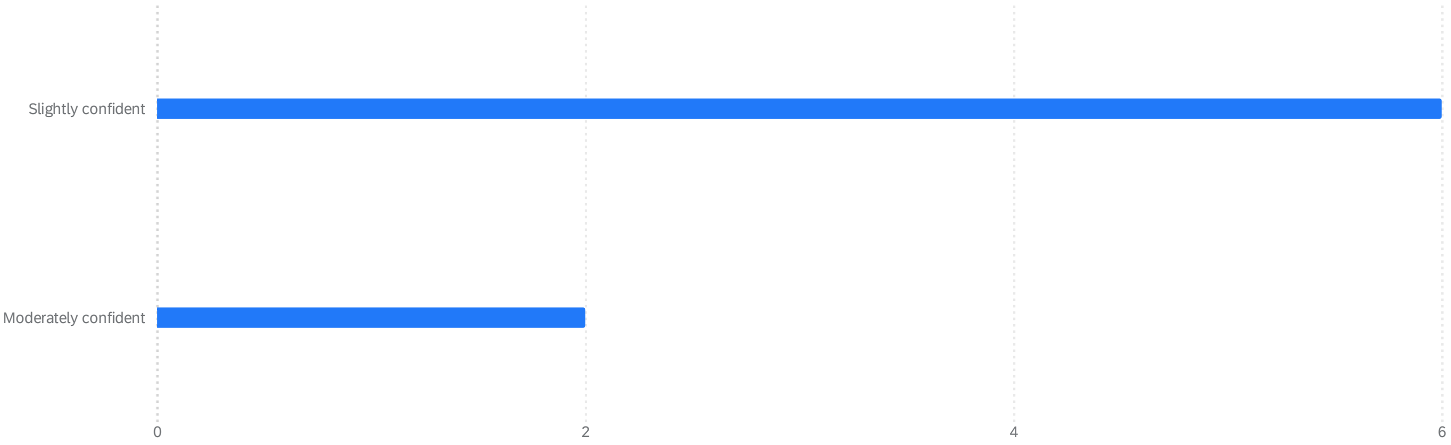

How confident are you in performing inpatient preoperative assessment? 8

Q8 - How confident are you in performing inpatient preoperative assessment?	Count	Count
Slightly confident	75%	6
Moderately confident	25%	2

How confident are you in performing inpatient preoperative assessment? 8

Q8 - How confident are you in performing inpatient preoperative assessment?	Average (Q8 - How confident are you in performing inpatient preoperative assessment?)	Minimum (Q8 - How confident are you in performing inpatient preoperative assessment?)	Maximum (Q8 - How confident are you in performing inpatient preoperative assessment?)	Count
Moderately confident	3.00	3.00	3.00	2
Slightly confident	2.00	2.00	2.00	6

How confident are you in applying the ACC/AHA guidelines for perioperative cardiovascular evaluation? 8

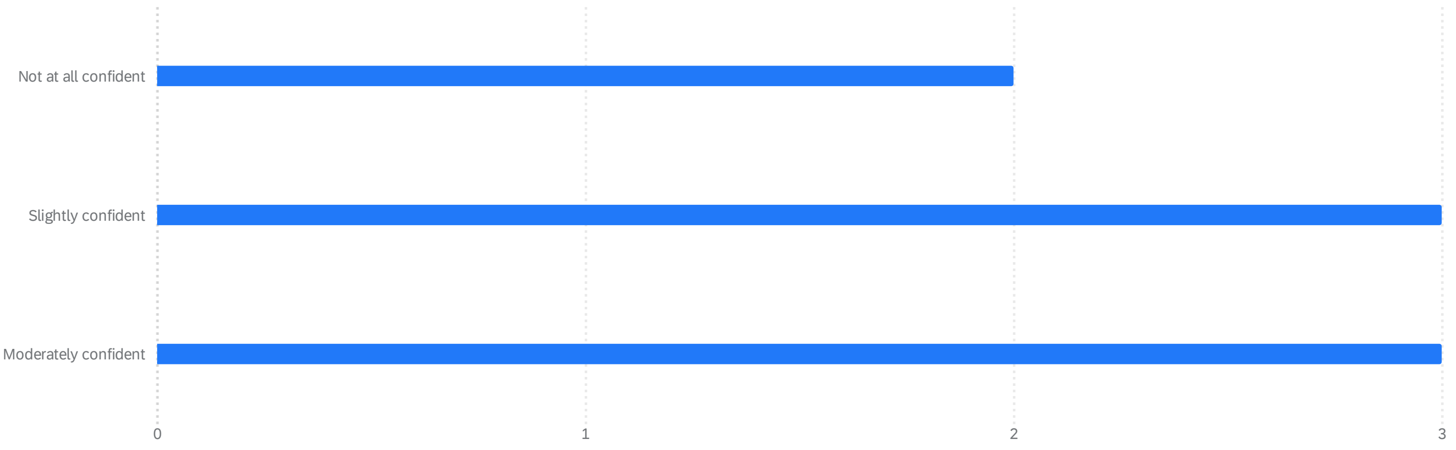

How confident are you in applying the ACC/AHA guidelines for perioperative cardiovascular evaluation? 8

Q9 - How confident are you in applying the ACC/AHA guidelines for perioperative cardiovascular evaluation?	Count	Count
Not at all confident	25%	2
Slightly confident	38%	3
Moderately confident	38%	3

How confident are you in applying the ACC/AHA guidelines for perioperative cardiovascular evaluation? 8

Q9 - How confident are you in applying the ACC/AHA guidelines for perioperative cardiovascular evaluation?	Average (Q9 - How confident are you in applying the ACC/AHA guidelines for perioperative cardiovascular evaluation?)	Minimum (Q9 - How confident are you in applying the ACC/AHA guidelines for perioperative cardiovascular evaluation?)	Maximum (Q9 - How confident are you in applying the ACC/AHA guidelines for perioperative cardiovascular evaluation?)	Count
Moderately confident	3.00	3.00	3.00	3
Not at all confident	1.00	1.00	1.00	2
Slightly confident	2.00	2.00	2.00	3

How confident are you in managing patients undergoing bariatric surgery preoperatively? 8

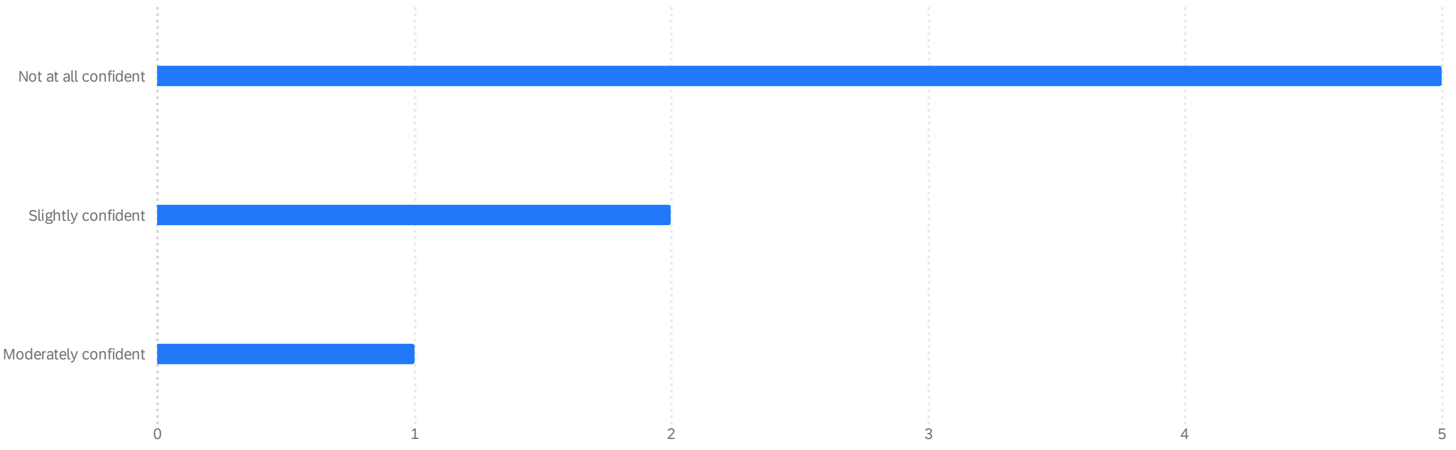

How confident are you in managing patients undergoing bariatric surgery preoperatively? 8

Q10 - How confident are you in managing patients undergoing bariatric surgery preoperatively?	Count	Count
Not at all confident	63%	5
Slightly confident	25%	2
Moderately confident	13%	1

How confident are you in managing patients undergoing bariatric surgery preoperatively? 8

Q10 - How confident are you in managing patients undergoing bariatric surgery preoperatively?	Average (Q10 - How confident are you in managing patients undergoing bariatric surgery preoperatively?)	Minimum (Q10 - How confident are you in managing patients undergoing bariatric surgery preoperatively?)	Maximum (Q10 - How confident are you in managing patients undergoing bariatric surgery preoperatively?)	Count
Moderately confident	3.00	3.00	3.00	1
Not at all confident	1.00	1.00	1.00	5
Slightly confident	2.00	2.00	2.00	2

How confident are you in managing patients with chronic kidney disease preoperatively? 8

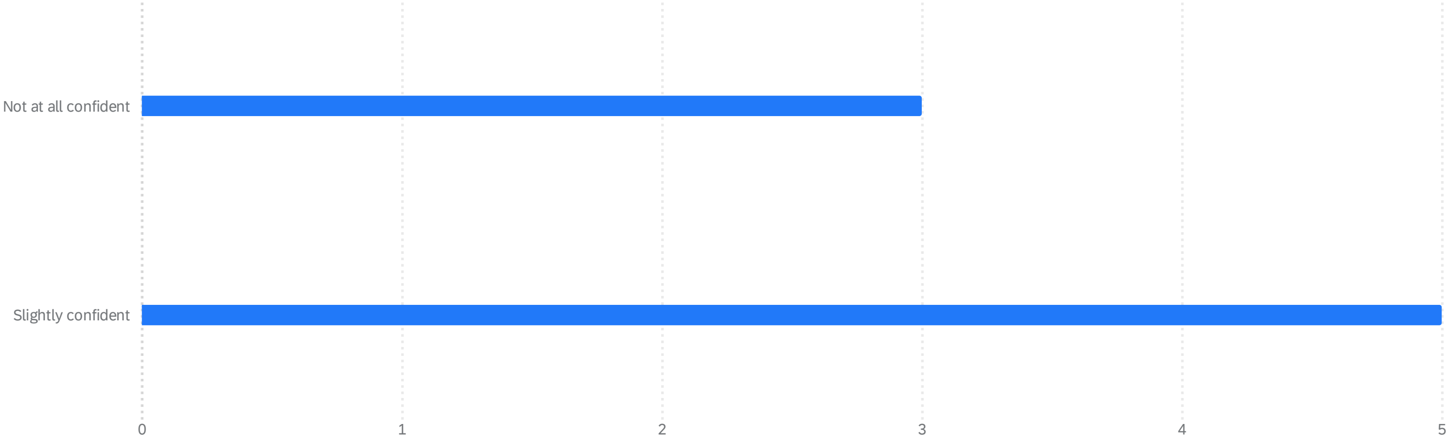

How confident are you in managing patients with chronic kidney disease preoperatively? 8

Q11 - How confident are you in managing patients with chronic kidney disease preoperatively?	Count	Count
Not at all confident	38%	3
Slightly confident	63%	5

How confident are you in managing patients with chronic kidney disease preoperatively? 8

Q11 - How confident are you in managing patients with chronic kidney disease preoperatively?	Average (Q11 - How confident are you in managing patients with chronic kidney disease preoperatively?)	Minimum (Q11 - How confident are you in managing patients with chronic kidney disease preoperatively?)	Maximum (Q11 - How confident are you in managing patients with chronic kidney disease preoperatively?)	Count
Not at all confident	1.00	1.00	1.00	3
Slightly confident	2.00	2.00	2.00	5

How confident are you in managing patients with liver disease preoperatively? 8

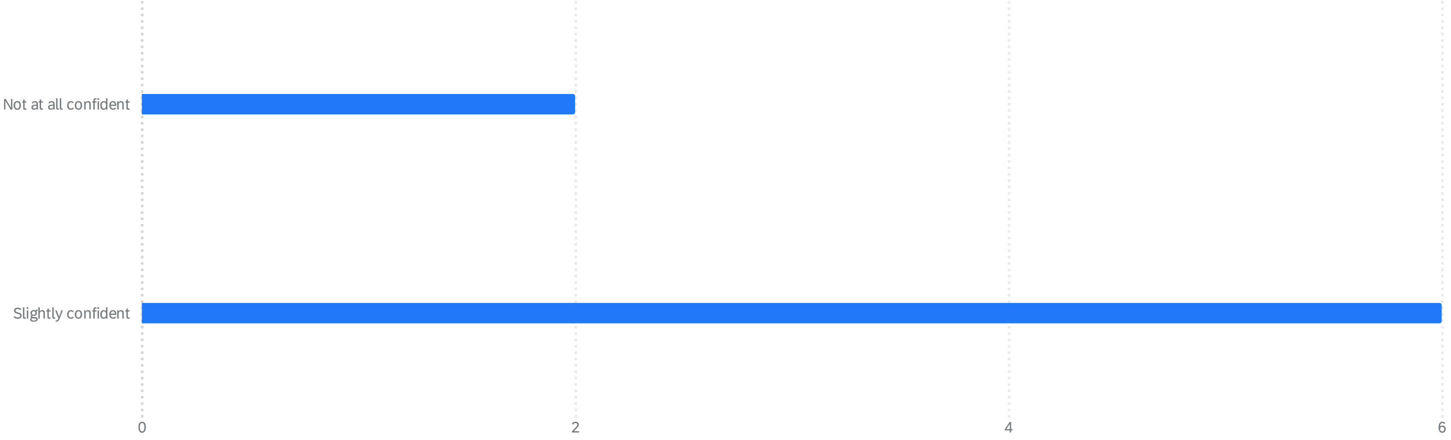

How confident are you in managing patients with liver disease preoperatively? 8

Q12 - How confident are you in managing patients with liver disease preoperatively?	Count	Count
Not at all confident	25%	2
Slightly confident	75%	6

How confident are you in managing patients with liver disease preoperatively? 8

Q12 - How confident are you in managing patients with liver disease preoperatively?	Average (Q12 - How confident are you in managing patients with liver disease preoperatively?)	Minimum (Q12 - How confident are you in managing patients with liver disease preoperatively?)	Maximum (Q12 - How confident are you in managing patients with liver disease preoperatively?)	Count
Not at all confident	1.00	1.00	1.00	2
Slightly confident	2.00	2.00	2.00	6

How confident are you in managing patients with rheumatologic conditions (e.g., RA) preoperatively? 8

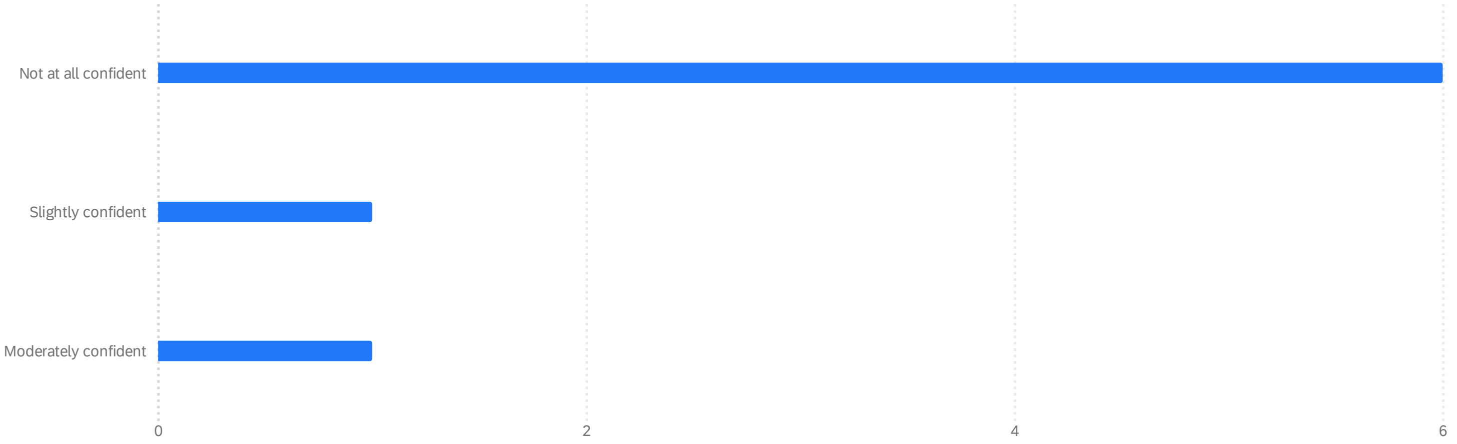

How confident are you in managing patients with rheumatologic conditions (e.g., RA) preoperatively? 8

Q13 - How confident are you in managing patients with rheumatologic conditions (e.g., RA) preoperatively?	Count	Count
Not at all confident	75%	6
Slightly confident	13%	1
Moderately confident	13%	1

How confident are you in managing patients with rheumatologic conditions (e.g., RA) preoperatively? 8

Q13 - How confident are you in managing patients with rheumatologic conditions (e.g., RA) preoperatively?	Average (Q13 - How confident are you in managing patients with rheumatologic conditions (e.g., RA) preoperatively?)	Minimum (Q13 - How confident are you in managing patients with rheumatologic conditions (e.g., RA) preoperatively?)	Maximum (Q13 - How confident are you in managing patients with rheumatologic conditions (e.g., RA) preoperatively?)	Count
Moderately confident	3.00	3.00	3.00	1

Q13 - How confident are you in managing patients with rheumatologic conditions (e.g., RA) preoperatively?	Average (Q13 - How confident are you in managing patients with rheumatologic conditions (e.g., RA) preoperatively?)	Minimum (Q13 - How confident are you in managing patients with rheumatologic conditions (e.g., RA) preoperatively?)	Maximum (Q13 - How confident are you in managing patients with rheumatologic conditions (e.g., RA) preoperatively?)	Count
Not at all confident	1.00	1.00	1.00	6
Slightly confident	2.00	2.00	2.00	1

Guidelines recommend utilizing risk calculators to estimate risk of major adverse cardiac events (MACE). What is the percentage cut off? 8

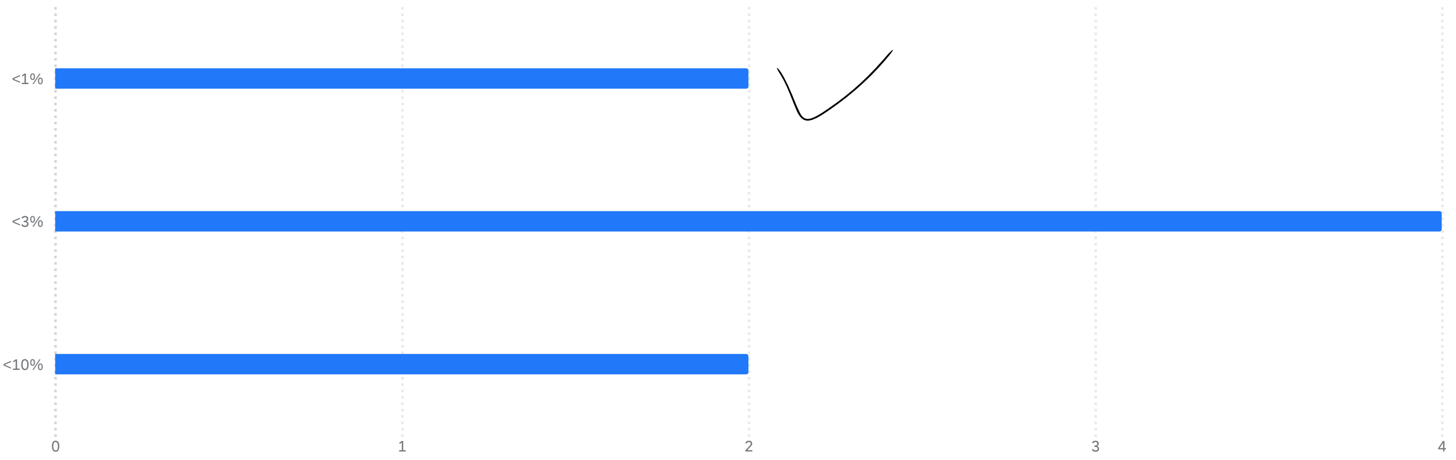

Guidelines recommend utilizing risk calculators to estimate risk of major adverse cardiac events (MACE). What is the percentage cut off? 8

Q14 - Guidelines recommend utilizing risk calculators to estimate risk of major adverse cardiac events (MACE). What is the percentage cut off?	Count	Count
<1%	25%	2
<3%	50%	4
<10%	25%	2

Guidelines recommend utilizing risk calculators to estimate risk of major adverse cardiac events (MACE). What is the percentage cut off? 8

Q14 - Guidelines recommend utilizing risk calculators to estimate risk of major adverse cardiac events (MACE). What is the percentage cut off?	Average (Q14 - Guidelines recommend utilizing risk calculators to estimate risk of major adverse cardiac events (MACE). What is the percentage cut off?)	Minimum (Q14 - Guidelines recommend utilizing risk calculators to estimate risk of major adverse cardiac events (MACE). What is the percentage cut off?)	Maximum (Q14 - Guidelines recommend utilizing risk calculators to estimate risk of major adverse cardiac events (MACE). What is the percentage cut off?)	Count
<1%	1.00	1.00	1.00	2
<10%	4.00	4.00	4.00	2
<3%	2.00	2.00	2.00	4

For patients who received bare metal stents, which of the following is a TRUE statement? 8

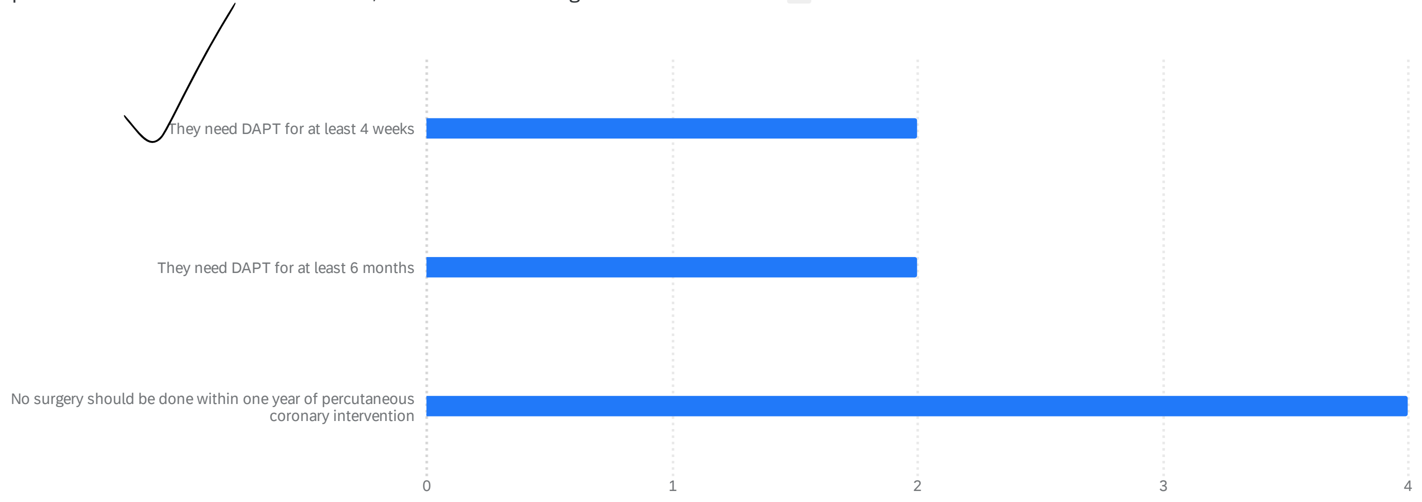

For patients who received bare metal stents, which of the following is a TRUE statement? 8

Q15 - For patients who received bare metal stents, which of the following is a TRUE statement?	Count	Count
They need DAPT for at least 4 weeks	25%	2
They need DAPT for at least 6 months	25%	2
No surgery should be done within one year of percutaneous coronary intervention	50%	4

For patients who received bare metal stents, which of the following is a TRUE statement? 8

Q15 - For patients who received bare metal stents, which of the following is a TRUE statement?	Average (Q15 - For patients who received bare metal stents, which of the following is a TRUE statement?)	Minimum (Q15 - For patients who received bare metal stents, which of the following is a TRUE statement?)	Maximum (Q15 - For patients who received bare metal stents, which of the following is a TRUE statement?)	Count
No surgery should be done within one year of percutaneous coronary intervention	3.00	3.00	3.00	4
They need DAPT for at least 4 weeks	1.00	1.00	1.00	2
They need DAPT for at least 6 months	2.00	2.00	2.00	2

For patients who received drug-eluting stents, what is the recommended duration for dual anti-platelet therapy? 8

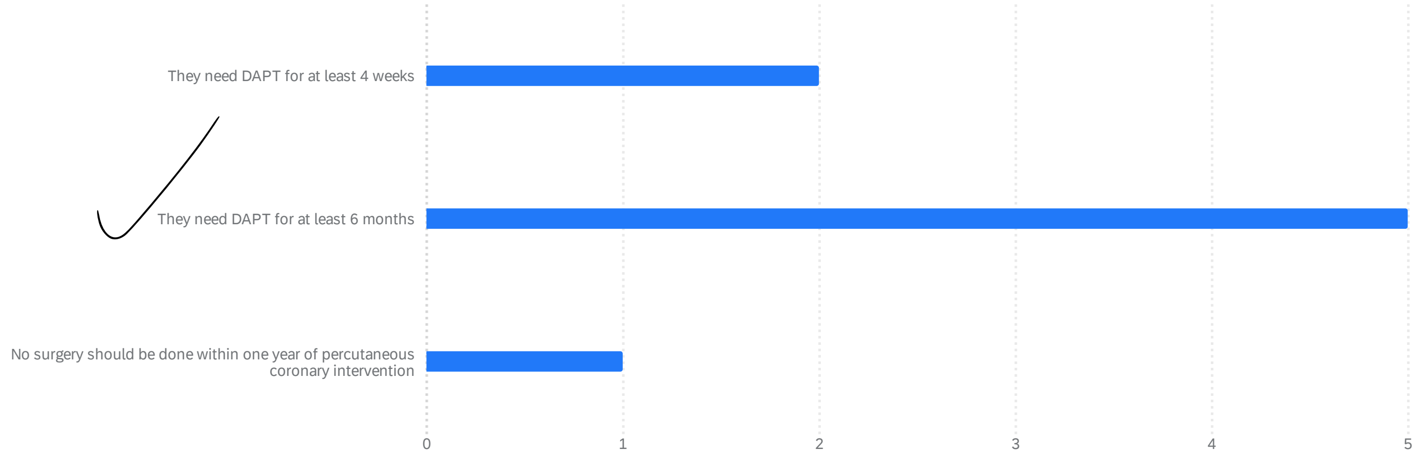

For patients who received drug-eluting stents, what is the recommended duration for dual anti-platelet therapy? 8

Q16 - For patients who received drug-eluting stents, what is the recommended duration for dual anti-platelet therapy?	Count	Count
They need DAPT for at least 4 weeks	25%	2
They need DAPT for at least 6 months	63%	5
No surgery should be done within one year of percutaneous coronary intervention	13%	1

For patients who received drug-eluting stents, what is the recommended duration for dual anti-platelet therapy? 8

Q16 - For patients who received drug-eluting stents, what is the recommended duration for dual anti-platelet therapy?	Average (Q16 - For patients who received drug-eluting stents, what is the recommended duration for dual anti-platelet therapy?)	Minimum (Q16 - For patients who received drug-eluting stents, what is the recommended duration for dual anti-platelet therapy?)	Maximum (Q16 - For patients who received drug-eluting stents, what is the recommended duration for dual anti-platelet therapy?)	Count
No surgery should be done within one year of percutaneous coronary intervention	3.00	3.00	3.00	1
They need DAPT for at least 4 weeks	1.00	1.00	1.00	2
They need DAPT for at least 6 months	2.00	2.00	2.00	5

Which statement is true? 8

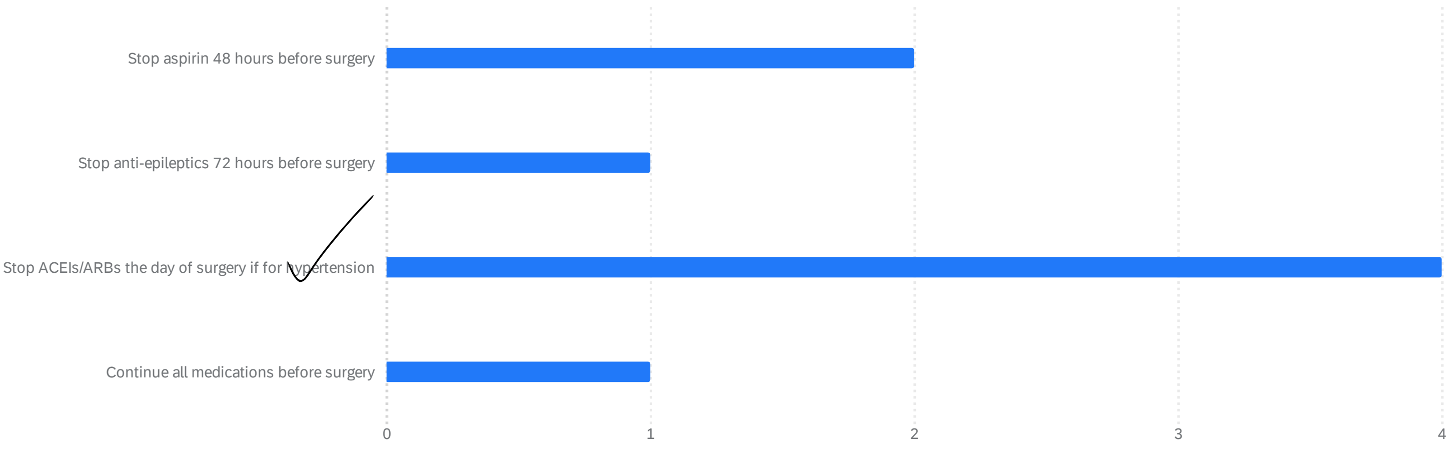

Which statement is true? 8

Q17 - Which statement is true?	Count	Count
Stop aspirin 48 hours before surgery	25%	2
Stop anti-epileptics 72 hours before surgery	13%	1
Stop ACEIs/ARBs the day of surgery if for hypertension	50%	4
Continue all medications before surgery	13%	1

Which statement is true? 8

Q17 - Which statement is true?	Average (Q17 - Which statement is true?)	Minimum (Q17 - Which statement is true?)	Maximum (Q17 - Which statement is true?)	Count
Continue all medications before surgery	4.00	4.00	4.00	1
Stop ACEIs/ARBs the day of surgery if for hypertension	3.00	3.00	3.00	4
Stop anti-epileptics 72 hours before surgery	2.00	2.00	2.00	1
Stop aspirin 48 hours before surgery	1.00	1.00	1.00	2

Which set of patients require stress dose steroids to prevent adrenal crisis during surgery? 8

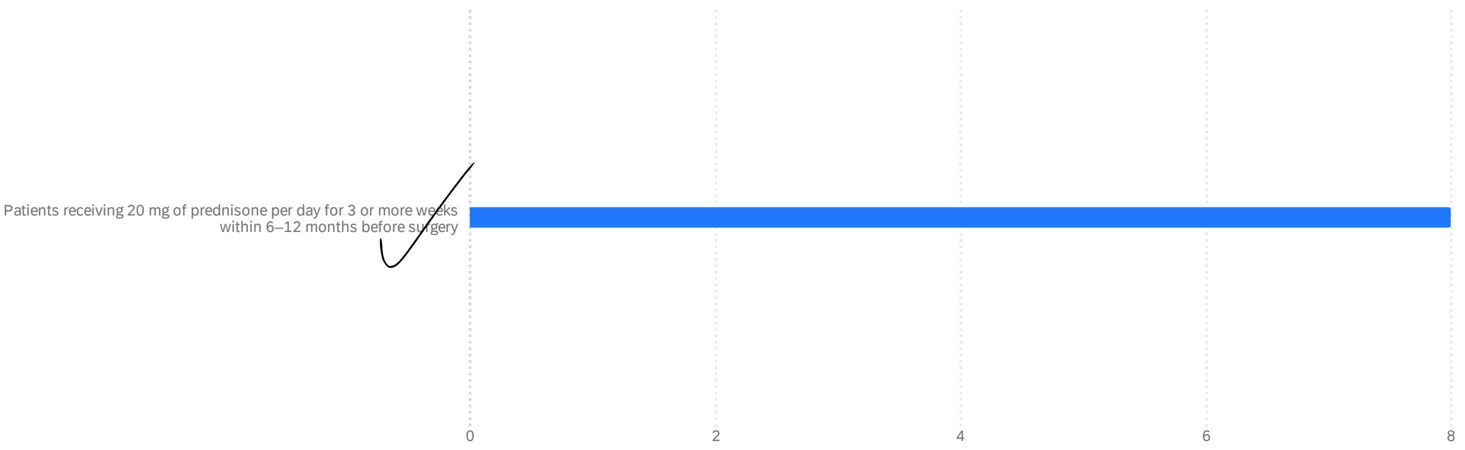

Which set of patients require stress dose steroids to prevent adrenal crisis during surgery? 8

Q18 - Which set of patients require stress dose steroids to prevent adrenal crisis during surgery?	Count	Count
Patients receiving 20 mg of prednisone per day for 3 or more weeks within 6–12 months before surgery	100%	8

Which set of patients require stress dose steroids to prevent adrenal crisis during surgery? 8

Q18 - Which set of patients require stress dose steroids to prevent adrenal crisis during surgery?	Average (Q18 - Which set of patients require stress dose steroids to prevent adrenal crisis during surgery?)	Minimum (Q18 - Which set of patients require stress dose steroids to prevent adrenal crisis during surgery?)	Maximum (Q18 - Which set of patients require stress dose steroids to prevent adrenal crisis during surgery?)	Count
Patients receiving 20 mg of prednisone per day for 3 or more weeks within 6–12 months before surgery	2.00	2.00	2.00	8

Which of the following statements are true regarding insulin management prior to surgery for patients with type I diabetes? (Select all that apply) 8

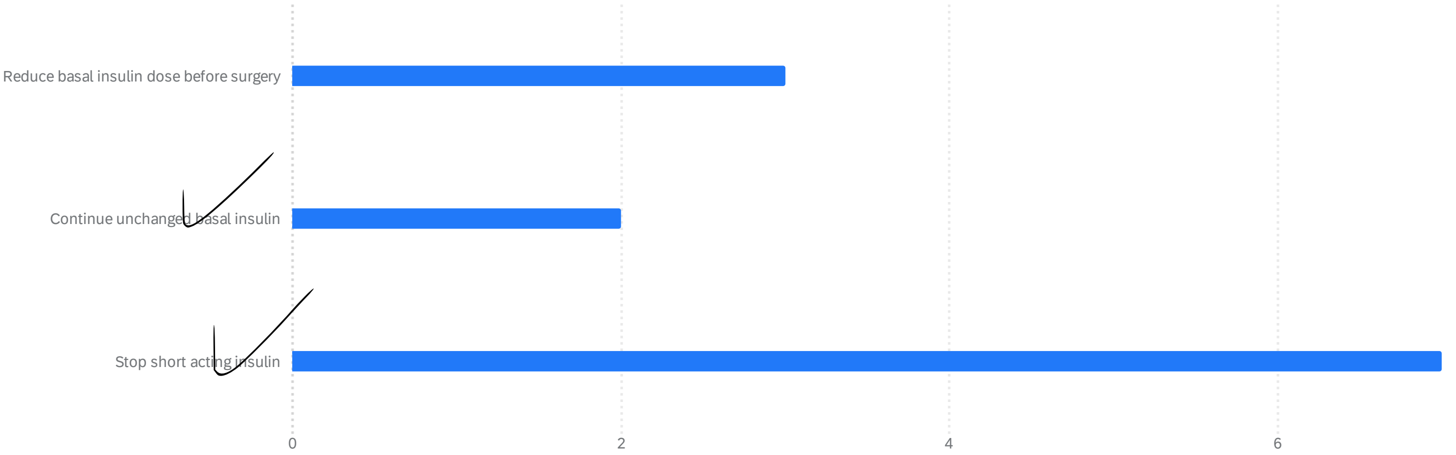

Which of the following statements are true regarding insulin management prior to surgery for patients with type I diabetes? (Select all that apply) 8

Q19 - Which of the following statements are true regarding insulin management prior to surgery for patients with type I diabetes? (Select all that apply)	Count	Count
Reduce basal insulin dose before surgery	38%	3
Continue unchanged basal insulin	25%	2
Stop short acting insulin	88%	7

Which statement regarding SGLT2 inhibitors is true? (Select all that apply) 8

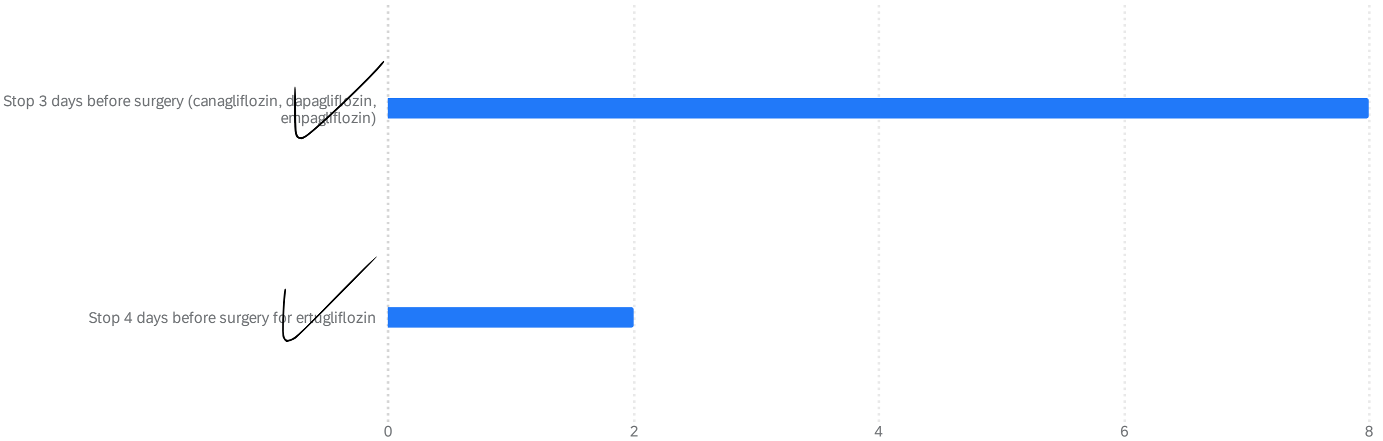

Which statement regarding SGLT2 inhibitors is true? (Select all that apply) 8

Q20 - Which statement regarding SGLT2 inhibitors is true? (Select all that apply)	Count	Count
Stop 3 days before surgery (canagliflozin, dapagliflozin, empagliflozin)	100%	8
Stop 4 days before surgery for ertugliflozin	25%	2

Which rheumatologic agents are safe to continue before surgery? (Select all that apply) 7

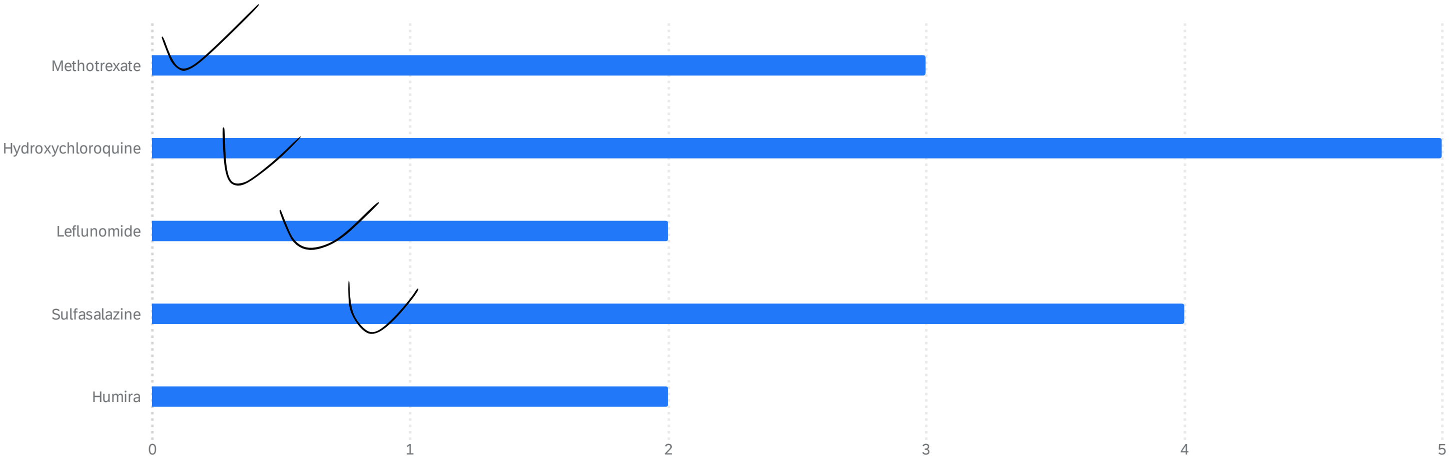

Which rheumatologic agents are safe to continue before surgery? (Select all that apply) 7

Q21 - Which rheumatologic agents are safe to continue before surgery? (Select all that apply)	Count	Count
Methotrexate	43%	3
Hydroxychloroquine	71%	5
Leflunomide	29%	2
Sulfasalazine	57%	4
Humira	29%	2

Which statement regarding bridging is true? (Select all that apply) 8

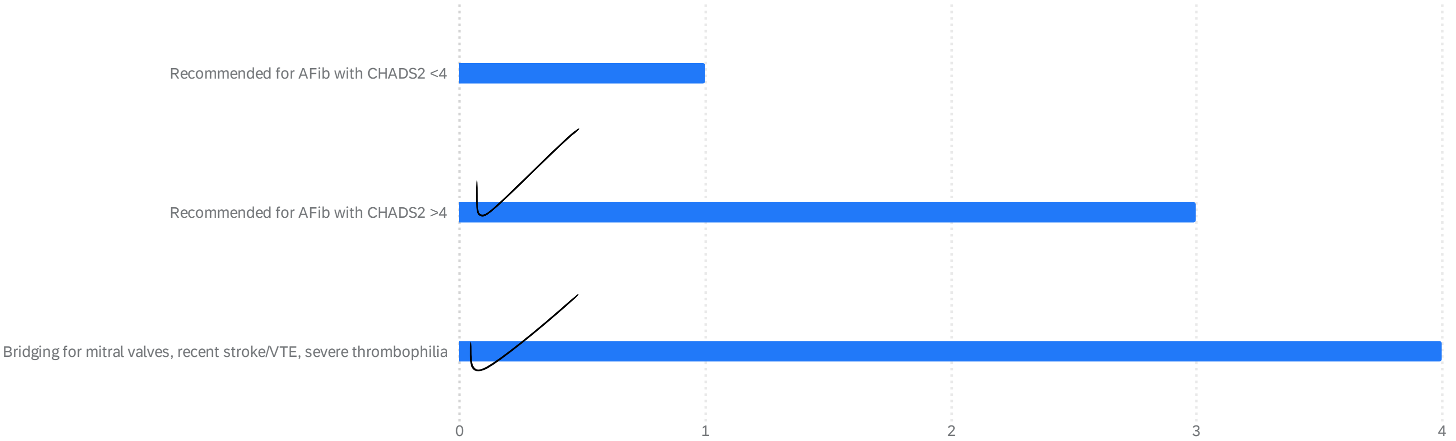

Which statement regarding bridging is true? (Select all that apply) 8

Q22 - Which statement regarding bridging is true? (Select all that apply)	Count	Count
Recommended for AFib with CHADS2 <4	13%	1
Recommended for AFib with CHADS2 >4	38%	3
Bridging for mitral valves, recent stroke/VTE, severe thrombophilia	50%	4

Which statement regarding bridging is true? (Select all that apply) 8

Q22 - Which statement regarding bridging is true? (Select all that apply)	Average (Q22 - Which statement regarding bridging is true? (Select all that apply))	Minimum (Q22 - Which statement regarding bridging is true? (Select all that apply))	Maximum (Q22 - Which statement regarding bridging is true? (Select all that apply))	Count
Bridging for mitral valves, recent stroke/VTE, severe thrombophilia	5.00	5.00	5.00	4
Recommended for AFib with CHADS2 <4	1.00	1.00	1.00	1
Recommended for AFib with CHADS2 >4	2.00	2.00	2.00	3

For patients on warfarin, you would check INR 7–10 days before the procedure. Which of the following is TRUE? (Select all that apply) 8

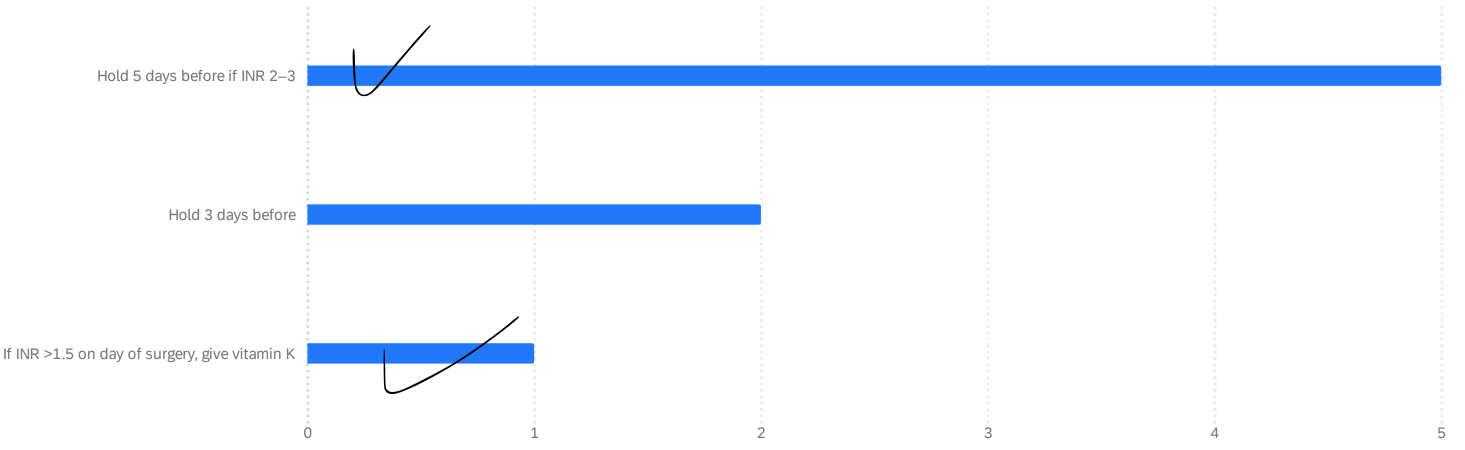

For patients on warfarin, you would check INR 7–10 days before the procedure. Which of the following is TRUE? (Select all that apply) 8

Q23 - For patients on warfarin, you would check INR 7–10 days before the procedure. Which of the following is TRUE? (Select all that apply)	Count	Count
Hold 5 days before if INR 2–3	63%	5
Hold 3 days before	25%	2
If INR >1.5 on day of surgery, give vitamin K	13%	1

For patients on warfarin, you would check INR 7–10 days before the procedure. Which of the following is TRUE? (Select all that apply) 8

Q23 - For patients on warfarin, you would check INR 7–10 days before the procedure. Which of the following is TRUE? (Select all that apply)	Average (Q23 - For patients on warfarin, you would check INR 7–10 days before the procedure. Which of the following is TRUE? (Select all that apply))	Minimum (Q23 - For patients on warfarin, you would check INR 7–10 days before the procedure. Which of the following is TRUE? (Select all that apply))	Maximum (Q23 - For patients on warfarin, you would check INR 7–10 days before the procedure. Which of the following is TRUE? (Select all that apply))	Count
Hold 3 days before	2.00	2.00	2.00	2
Hold 5 days before if INR 2–3	1.00	1.00	1.00	5
If INR >1.5 on day of surgery, give vitamin K	5.00	5.00	5.00	1

How do you restart warfarin after low-moderate bleeding risk procedure? 8

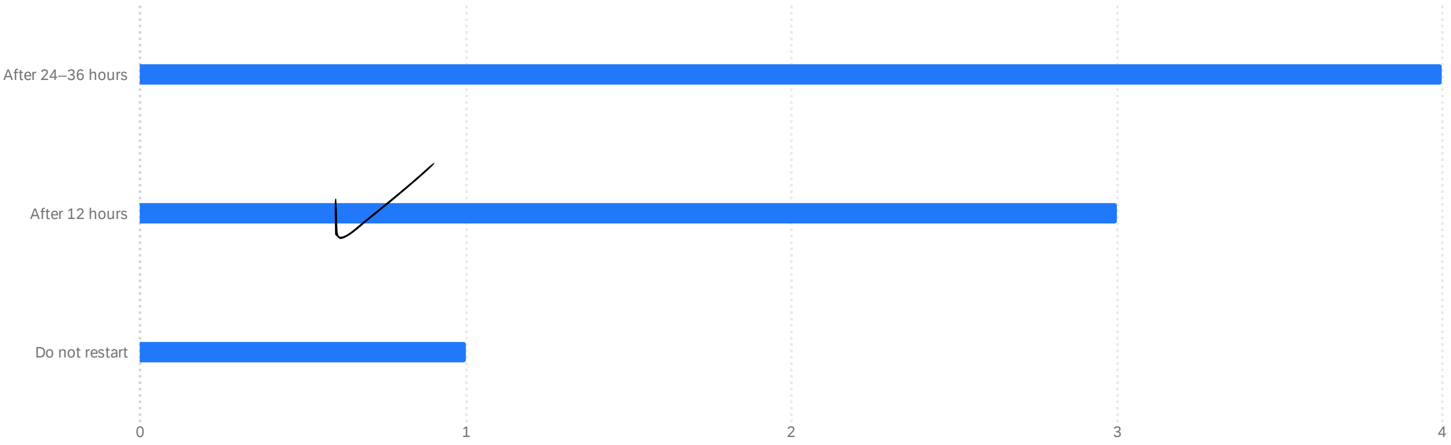

How do you restart warfarin after low-moderate bleeding risk procedure? 8

Q24 - How do you restart warfarin after low-moderate bleeding risk procedure?	Count	Count
After 24–36 hours	50%	4
After 12 hours	38%	3
Do not restart	13%	1

How do you restart warfarin after low-moderate bleeding risk procedure? 8

Q24 - How do you restart warfarin after low-moderate bleeding risk procedure?	Average (Q24 - How do you restart warfarin after low-moderate bleeding risk procedure?)	Minimum (Q24 - How do you restart warfarin after low-moderate bleeding risk procedure?)	Maximum (Q24 - How do you restart warfarin after low-moderate bleeding risk procedure?)	Count
After 12 hours	2.00	2.00	2.00	3
After 24–36 hours	1.00	1.00	1.00	4
Do not restart	3.00	3.00	3.00	1

Which is true regarding DOACs and normal CrCl? 7

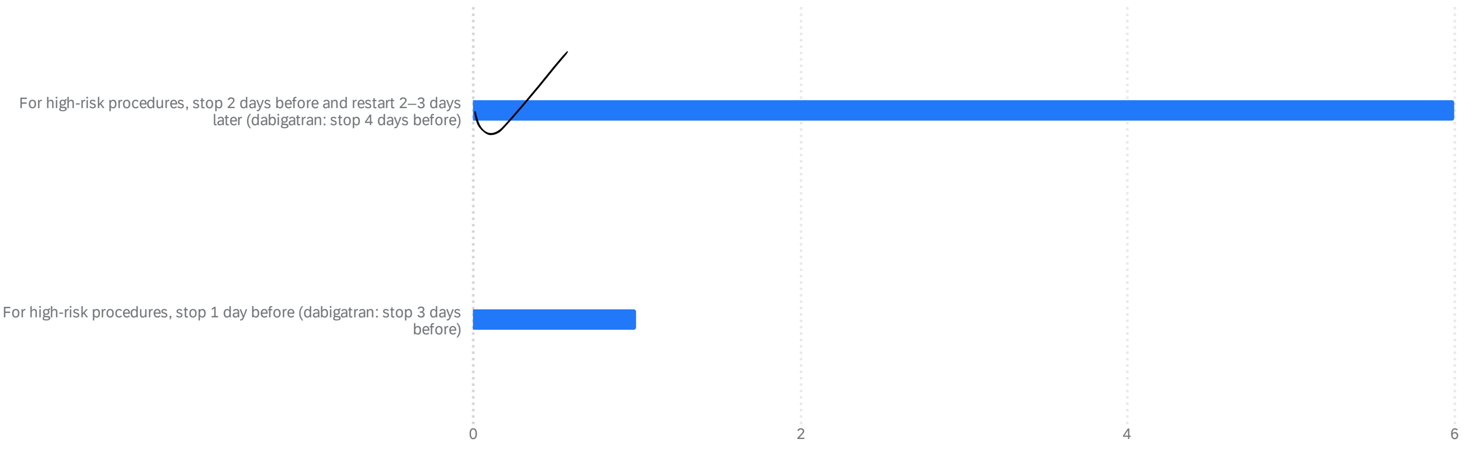

Which is true regarding DOACs and normal CrCl? 7

Q25 - Which is true regarding DOACs and normal CrCl?	Count	Count
For high-risk procedures, stop 2 days before and restart 2–3 days later (dabigatran: stop 4 days before)	86%	6
For high-risk procedures, stop 1 day before (dabigatran: stop 3 days before)	14%	1

Which is true regarding DOACs and normal CrCl? 7

Q25 - Which is true regarding DOACs and normal CrCl?	Average (Q25 - Which is true regarding DOACs and normal CrCl?)	Minimum (Q25 - Which is true regarding DOACs and normal CrCl?)	Maximum (Q25 - Which is true regarding DOACs and normal CrCl?)	Count
For high-risk procedures, stop 1 day before (dabigatran: stop 3 days before)	2.00	2.00	2.00	1
For high-risk procedures, stop 2 days before and restart 2–3 days later (dabigatran: stop 4 days before)	1.00	1.00	1.00	6

What calculator(s) can be used to assess VTE risk in bariatric surgery patients? (Select all that apply) 7

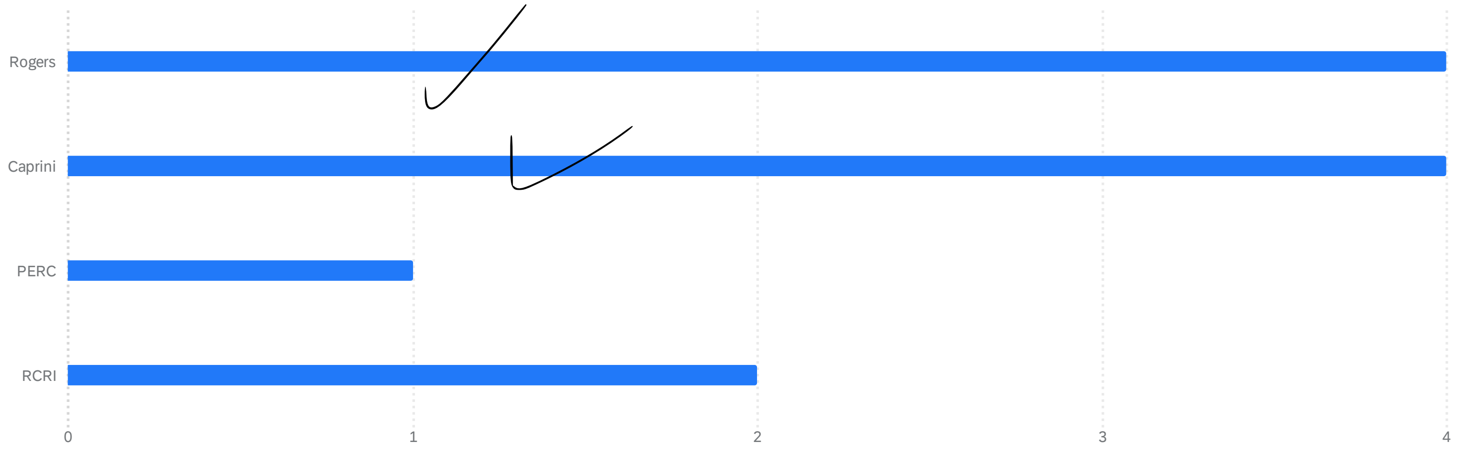

What calculator(s) can be used to assess VTE risk in bariatric surgery patients? (Select all that apply) 7

Q26 - What calculator(s) can be used to assess VTE risk in bariatric surgery patients? (Select all that apply)	Count	Count
Rogers	57%	4
Caprini	57%	4
PERC	14%	1
RCRI	29%	2
