# Supplementary material for: Escaping the OR: a pilot study of a Jigsaw-based workshop to teach preoperative assessment in internal medicine residency
Source: BMC Med Educ. 2026 May 16;26:1097. doi: 10.1186/s12909-026-09419-w (PMC13348809; doi:10.1186/s12909-026-09419-w)
Supplement: Supplementary file 1 — Supplementary Material 1. [file 12909_2026_9419_MOESM1_ESM.zip › Pre Op Needs Assessment Final.docx]

# Preoperative Medicine Needs Assessment (Inpatient vs. Outpatient)

Internal Medicine Residency Program

## What is your current PGY level?

☐ PGY-1

☐ PGY-2

☐ PGY-3

## How confident do you feel in performing a preoperative cardiac risk assessment in the inpatient setting?

☐ Not at all confident

☐ Slightly confident

☐ Moderately confident

☐ Very confident

☐ Extremely confident

## How confident do you feel in performing a preoperative cardiac risk assessment in the outpatient setting?

☐ Not at all confident

☐ Slightly confident

☐ Moderately confident

☐ Very confident

☐ Extremely confident

**How confident are you in applying the ACC/AHA guidelines for perioperative cardiovascular evaluation in clinical practice?**

☐ Not at all confident

☐ Slightly confident

☐ Moderately confident

☐ Very confident

☐ Extremely confident

## How comfortable are you with inpatient perioperative medication management?

☐ Not at all confident

☐ Slightly confident

☐ Moderately confident

☐ Very confident

☐ Extremely confident

## How comfortable are you with outpatient perioperative medication management?

☐ Not at all confident

☐ Slightly confident

☐ Moderately confident

☐ Very confident

☐ Extremely confident

## How confident are you in managing patients on anticoagulants preoperatively in the inpatient setting?

☐ Not at all confident

☐ Slightly confident

☐ Moderately confident

☐ Very confident

☐ Extremely confident

## How confident are you in managing patients on anticoagulants preoperatively in the outpatient setting?

☐ Not at all confident

☐ Slightly confident

☐ Moderately confident

☐ Very confident

☐ Extremely confident

## Have you had any formal education on perioperative medicine during residency in inpatient setting?

☐ Yes

☐ No

## Have you had any formal education on perioperative medicine during residency in outpatient setting?

☐ Yes

☐ No

## How confident are you in managing patients undergoing bariatric surgery preoperatively?

☐ Not at all confident

☐ Slightly confident

☐ Moderately confident

☐ Very confident

☐ Extremely confident

## How confident are you in managing patients with chronic kidney disease preoperatively?

☐ Not at all confident

☐ Slightly confident

☐ Moderately confident

☐ Very confident

☐ Extremely confident

## How confident are you in managing patients with liver disease preoperatively?

☐ Not at all confident

☐ Slightly confident

☐ Moderately confident

☐ Very confident

☐ Extremely confident

## How confident are you in managing patients with obstructive sleep apnea (OSA) preoperatively?

☐ Not at all confident

☐ Slightly confident

☐ Moderately confident

☐ Very confident

☐ Extremely confident

## How confident are you in managing patients with rheumatologic conditions (e.g., RA) preoperatively?

☐ Not at all confident

☐ Slightly confident

☐ Moderately confident

☐ Very confident

☐ Extremely confident

## What do you perceive as the most challenging topics in perioperative medicine? (Select all that apply)

☐ Cardiac risk stratification

☐ Medication management

☐ Anticoagulation management

☐ Optimization of patients with complex comorbidities

☐ Communication with surgical/anesthesia teams

☐ Other: __________
