# Supplementary material for: Escaping the OR: a pilot study of a Jigsaw-based workshop to teach preoperative assessment in internal medicine residency
Source: BMC Med Educ. 2026 May 16;26:1097. doi: 10.1186/s12909-026-09419-w (PMC13348809; doi:10.1186/s12909-026-09419-w)
Supplement: Supplementary file 1 — Supplementary Material 1. [file 12909_2026_9419_MOESM1_ESM.zip › Supplement 1.pdf]

Pre- and Post-Intervention Survey

Internal Medicine Residency Program

Demographics

Age: _____

Gender:

☐ Male

☐ Female

☐ Other

☐ Prefer not to say

Ethnicity (Check all that apply):

☐ Asian/Pacific Islander

☐ Caucasian

☐ Black/African American

☐ Hispanic/Latino

☐ Native American

☐ Other: ______

☐ Prefer not to answer

Please indicate your PGY year:

☐ PGY1

☐ PGY2

☐ PGY3

Prior Experience

Have you received training in ambulatory preoperative assessment in the following settings?

☐ Undergraduate coursework

☐ Medical school

☐ Graduate school

☐ Residency

☐ Self-study

☐ None

☐ Other: __________

Have you received training in inpatient preoperative assessment in the following settings?

☐ Undergraduate coursework

☐ Medical school

☐ Graduate school

☐ Residency

☐ Self-study

☐ None

☐ Other: __________

Confidence Ratings (Use the same scale for all):

How confident are you in performing ambulatory preoperative assessment?

☐ Not at all confident

☐ Slightly confident

☐ Moderately confident

☐ Very confident

☐ Extremely confident

How confident are you in performing inpatient preoperative assessment?

☐ Not at all confident

☐ Slightly confident

☐ Moderately confident

☐ Very confident

☐ Extremely confident

How confident are you in applying the ACC/AHA guidelines for perioperative cardiovascular evaluation?

☐ Not at all confident

☐ Slightly confident

☐ Moderately confident

☐ Very confident

☐ Extremely confident

How confident are you in managing patients undergoing bariatric surgery preoperatively?

☐ Not at all confident

☐ Slightly confident

☐ Moderately confident

☐ Very confident

☐ Extremely confident

How confident are you in managing patients with chronic kidney disease preoperatively?

☐ Not at all confident

☐ Slightly confident

☐ Moderately confident

☐ Very confident

☐ Extremely confident

How confident are you in managing patients with liver disease preoperatively?

☐ Not at all confident

☐ Slightly confident

☐ Moderately confident

☐ Very confident

☐ Extremely confident

How confident are you in managing patients with obstructive sleep apnea (OSA) preoperatively?

☐ Not at all confident

☐ Slightly confident

☐ Moderately confident

☐ Very confident

☐ Extremely confident

How confident are you in managing patients with rheumatologic conditions (e.g., RA) preoperatively?

☐ Not at all confident

☐ Slightly confident

☐ Moderately confident

☐ Very confident

☐ Extremely confident

Knowledge Questions

Guidelines recommend utilizing risk calculators to estimate risk of major adverse cardiac events (MACE). What is the percentage cut off?

x <1%
☐ <3%
☐ <5%
☐ <10%

For patients who received bare metal stents, which of the following is a TRUE statement?

x They need DAPT for at least 4 weeks
☐ They need DAPT for at least 6 months
☐ No surgery should be done within one year of percutaneous coronary intervention

For patients who received drug-eluting stents, what is the recommended duration for dual anti-platelet therapy?

☐ They need DAPT for at least 4 weeks
x They need DAPT for at least 6 months
☐ No surgery should be done within one year of percutaneous coronary intervention

Which statement is true?

☐ Stop aspirin 48 hours before surgery
☐ Stop anti-epileptics 72 hours before surgery
x Stop ACEIs/ARBs the day of surgery if for hypertension
☐ Continue all medications before surgery

Which set of patients require stress dose steroids to prevent adrenal crisis during surgery?

☐ Patients who had chronic COPD/asthma with bursts of prednisone one year ago
x Patients receiving 20 mg of prednisone per day for 3 or more weeks within 6–12 months before surgery
☐ Patients with diabetes
☐ Patients with hyperthyroidism

Which of the following statements are true regarding insulin management prior to surgery for patients with type I diabetes? (Select all that apply)

☐ Reduce basal insulin dose before surgery

x Continue unchanged basal insulin

☐ Stop basal insulin completely

☐ Continue short acting insulin

x Stop short acting insulin

Which statement regarding SGLT2 inhibitors is true? (Select all that apply)

x Stop 3 days before surgery (canagliflozin, dapagliflozin, empagliflozin)

x Stop 4 days before surgery for ertugliflozin

☐ Do not stop these medications

Which rheumatologic agents are safe to continue before surgery? (Select all that apply)

x Methotrexate

x Hydroxychloroquine

x Leflunomide

x Sulfasalazine

☐ Humira

Which statement regarding bridging is true? (Select all that apply)

☐ Recommended for AFib with CHADS2 <4

x Recommended for AFib with CHADS2 >4

☐ Bridging regardless of CHADS2

☐ Bridging not recommended due to bleeding risk

x Bridging for mitral valves, recent stroke/VTE, severe thrombophilia

For patients on warfarin, you would check INR 7–10 days before the procedure. Which of the following is TRUE? (Select all that apply)

x Hold 5 days before if INR 2–3

☐ Hold 3 days before

☐ Hold 1 day before

☐ Do not hold warfarin

x If INR >1.5 on day of surgery, give vitamin K

How do you restart warfarin after low-moderate bleeding risk procedure?

☐ After 24–36 hours

x After 12 hours

☐ Do not restart

Which is true regarding DOACs and normal CrCl?

x For high-risk procedures, stop 2 days before and restart 2–3 days later (dabigatran: stop 4 days before)

☐ For high-risk procedures, stop 1 day before (dabigatran: stop 3 days before)

☐ DOACs do not require interruption

What calculator(s) can be used to assess VTE risk in bariatric surgery patients? (Select all that apply)

x Rogers

x Caprini

☐ Well's

☐ PERC

☐ RCRI

☐ GUPTA

Open Feedback

Any feedback about the curriculum or preoperative medicine project?

____

____

____
