# Supplementary material for: Escaping the OR: a pilot study of a Jigsaw-based workshop to teach preoperative assessment in internal medicine residency
Source: BMC Med Educ. 2026 May 16;26:1097. doi: 10.1186/s12909-026-09419-w (PMC13348809; doi:10.1186/s12909-026-09419-w)
Supplement: Supplementary file 1 — Supplementary Material 1. [file 12909_2026_9419_MOESM1_ESM.zip › Response to Reviewers 01232026.docx]

**Response to Reviewers**

We sincerely thank the reviewer for their thoughtful and constructive comments. We appreciate the opportunity to clarify the intent, scope, and contribution of this work and have revised the manuscript accordingly. Below, we respond point-by-point to the major comments.

**Reviewer Comment 1: Low Response Rate / Limited Sample Size**

*The very small number of participants significantly limits statistical power and the confidence with which conclusions can be drawn. It is currently unclear whether the absence of statistically significant findings reflects true similarity between groups or simply insufficient sample size. Please explicitly discuss how these limitations affect interpretation of the results, what meaningful insights can still be drawn, why collecting additional data was not feasible, and why it is reasonable to draw preliminary conclusions.*

**Response:**

We agree with the reviewer that the limited sample size restricts statistical power and limits definitive inference. We have revised the Discussion to explicitly address how this limitation affects interpretation of the findings, emphasizing that the absence of statistically significant differences should not be interpreted as evidence of equivalence between instructional approaches, but rather as a consequence of limited power in a pilot study.

We have also clarified the rationale for cohort selection and sample size. This study represents the original pilot implementation of the curriculum within a primary care residency track, which was intentionally selected because it offered a controlled educational environment with protected didactic time and curricular flexibility. This structure allowed for mid-year integration of the intervention, whereas the categorical internal medicine residency curriculum was fully scheduled for the academic year and did not permit insertion of a new educational intervention during the study period. We have now explicitly stated this rationale in the Methods and Discussion.

Regarding feasibility of additional data collection, we have clarified that expansion beyond this cohort was not possible within the study timeframe without restructuring the residency curriculum or introducing contamination across training groups. As such, the study was designed to prioritize feasibility, acceptability, and preliminary signal detection rather than definitive comparative effectiveness.

Finally, we have strengthened the justification for drawing preliminary conclusions by framing this work as an early-phase educational pilot. We now explicitly state that pilot studies serve an essential role in curricular development by assessing feasibility, learner engagement, and potential educational impact prior to broader implementation. The findings from this study informed subsequent refinement and adaptation of the curriculum for larger and more heterogeneous learner populations.

**Reviewer Comment 2: Effect Size Reporting**

*Given the limited sample size, reporting effect sizes for all relevant analyses is essential.*

**Response:**

We agree and have revised the Results to include effect size estimates for all primary analyses. Given the pilot nature of the study and limited power to detect statistically significant differences, effect sizes provide important information regarding the magnitude and potential educational relevance of observed changes. We have also expanded the Discussion to contextualize these effect sizes appropriately, emphasizing their role in hypothesis generation and future study planning rather than definitive inference.

**Reviewer Comment 3: Applicability to an International Audience**

*Please expand the Discussion to address the relevance of the findings to international clinical education contexts and clearly articulate what clinical educators can reasonably learn from this work.*

**Response:**

We appreciate this suggestion and have expanded the Discussion to address the broader relevance of the educational model. While this pilot was conducted within a U.S.-based primary care residency program, we now explicitly discuss how the Jigsaw teaching method is grounded in adult learning theory and is adaptable across healthcare systems and international training environments.

We emphasize that preoperative medical assessment is a globally shared competency for general internists and that the curricular domains addressed in this study are relevant across diverse clinical contexts. Importantly, we clarify that the primary contribution of this work is the demonstration of feasibility and transferability of the instructional model rather than the generalizability of outcome magnitudes. We now clearly articulate what educators can reasonably conclude from this study, given its methodological constraints.

We thank the reviewer again for their insightful feedback, which has strengthened the clarity, rigor, and framing of this manuscript. We believe the revisions more clearly position this work as a feasibility-focused pilot study that contributes meaningfully to the educational literature and informs future curricular development.
